# Identification of *IL7R* as a key genetic risk locus in childhood steroid-sensitive nephrotic syndrome and IgA nephropathy

**DOI:** 10.3389/fimmu.2026.1806680

**Published:** 2026-05-29

**Authors:** Cong Wang, Yue Jiang, Yingchao Song, Minle Tian, Xiaoyuan Wang, Jing Wang, Qian Li, Ruixian Zang, Zhenle Yang, Lichun Yu, Suwen Liu, Li Wang, Xiujun Yao, Aihua Zhou, Hongwei Yu, Kalim Ullah, Joseph Glessner, Hakon Hakonarson, Shuzhen Sun, Xiao Chang

**Affiliations:** 1 Department of Pediatric Nephrology and Rheumatism and Immunology, Shandong Provincial Hospital Affiliated to Shandong First Medical University, Jinan, China; 2 College of Medical Information and Artificial Intelligence, Shandong First Medical University, Jinan, China; 3 Department of Neurology, Shandong Provincial Hospital affiliated to Shandong First Medical University, Jinan, China; 4 The Center for Applied Genomics, Children’s Hospital of Philadelphia, PA, United States

**Keywords:** genome-wide association studies, IgA nephropathy, IL7R, pediatric steroid-sensitive nephrotic syndrome, shared genetic architecture

## Abstract

**Introduction:**

Pediatric steroid-sensitive nephrotic syndrome (pSSNS) is a common childhood glomerular disorder characterized by corticosteroid responsiveness, yet frequent relapses and steroid dependence lead to long-term complications. While GWAS have identified genetic risk loci for pSSNS, its shared genetic architecture with immune-mediated glomerulopathies like IgA nephropathy (IgAN) remains unclear.

**Methods:**

We performed integrative genetic analyses combining GWAS data from pSSNS (2,440 cases/36,023 controls) and IgAN (10,146 cases/28,751 controls) through meta-analysis and conjunctional false discovery rate (conjFDR) approaches. Findings were replicated in an independent Chinese pSSNS cohort (501 cases/2,506 controls). Transcriptomic profiling of peripheral blood and renal tissues, supplemented by single-cell RNA sequencing, elucidated molecular mechanisms.

**Results:**

Meta-analysis identified nine genome-wide significant loci (P < 5×10^-8^), including five novel regions at 1q23.1, 1p36.13, 5p13.2, 10q21.3, and 10q24.1. ConjFDR analysis revealed 19 pleiotropic loci, with the 5p13.2 (*IL7R*) locus confirmed by both methods. This signal was replicated in an independent Chinese pSSNS cohort. Transcriptomic analyses using bulk RNA sequencing further revealed IL7R dysregulation associated with steroid response in pSSNS and with disease status in IgAN. Moreover, single-cell RNA sequencing highlighted IL7R dysregulation in CD4^+^ and CD8^+^ T cells and NK cells in nephrotic syndrome patients.

**Discussion:**

Our study establishes IL7R as a key shared genetic risk locus in pSSNS and IgAN, supported by multi-omics evidence of immune cell-specific dysregulation. These findings implicate IL7R-mediated T cell homeostasis in the pathogenesis of both disorders, nominating this pathway for targeted therapeutic development.

## Introduction

Pediatric steroid-sensitive nephrotic syndrome (pSSNS) is a common glomerular disorder in children, characterized by the rapid onset of nephrotic syndrome that responds to corticosteroid therapy. It typically presents with heavy proteinuria, hypoalbuminemia, and edema. The majority of children with pSSNS show significant improvement within the first four weeks of corticosteroid treatment, distinguishing it from steroid-resistant nephrotic syndrome (SRNS), which presents more complex treatment challenges (1). Although pSSNS is generally considered a benign disease, relapses are frequent, and recurrent or steroid-dependent relapses increase the risk of long-term renal complications (2–5). The precise pathogenesis of pSSNS remains unclear, but it is widely believed to involve immune system dysregulation, with aberrant immune responses playing a crucial role in disease progression (6, 7). Genetic, environmental, and immune factors are thought to contribute to its multifactorial etiology. Due to its clinical significance and the importance of early intervention, pSSNS remains a central focus of research in pediatric nephrology, aimed at improving treatment strategies and long-term outcomes for affected children.

Recent genome-wide association studies (GWAS) have provided valuable insights into the genetic basis of pSSNS. The largest GWAS of pSSNS to date, which included 2,440 cases and 36,023 controls, identified eight significant risk loci through a multi-population meta-analysis, indicating the polygenic nature of the disease (8). Despite these advances, many aspects of the genetic architecture remain unexplored, and additional loci are still to be identified. Larger cohorts and advanced analytical methods are expected to yield further insights into the disease’s genetic complexity. Interestingly, while clinical studies generally do not recognize a direct link between pSSNS and IgA nephropathy (IgAN), which is the most common form of primary glomerulonephritis and is characterized by the deposition of IgAN in the mesangium (9–11), we found that three of the eight pSSNS risk loci identified in the GWAS have also been reported in IgAN, including *CD28*, *TNFSF15*, and the MHC region (12). This suggests a potential genetic connection between pSSNS and IgAN, implying the possible shared immune-mediated pathways underlying both diseases.

In this study, we conducted a joint GWAS analysis to identify novel pleiotropic loci shared between pSSNS and IgAN. To validate these findings, we leveraged an independent Chinese pSSNS cohort and performed an additional GWAS to replicate the identified loci. Furthermore, we analyzed bulk RNA sequencing data from pSSNS and IgAN patients, complemented by single-cell RNA sequencing (scRNA-seq) data from nephrotic syndrome patients, to investigate the potential functional roles of candidate genes. Together, these integrative approaches aim to advance our understanding of the genetic architecture of pSSNS, with a particular focus on the shared immunogenetic mechanisms underlying its overlap with IgAN.

## Materials and methods

### Study design overview

The overall study design and analytical workflow are summarized in a schematic flow diagram (**Supplementary Figure 1**), which outlines the sequential study pipeline including GWAS dataset collection for pSSNS and IgAN, cross-trait genetic association analyses (inverse-variance weighted meta-analysis and conjunctional false discovery rate analysis), identification of shared risk loci, replication in an independent Chinese cohort, and downstream functional characterization using bulk RNA-seq and single-cell RNA-seq data.

### GWAS data of pSSNS and IgAN

The GWAS summary statistics for pSSNS were derived from a multi-population GWAS meta-analysis comprising 2,440 cases and 36,023 controls from populations of Admixed American, African, East Asian, European, Maghrebian, and South Asian ancestries (8). Similarly, the GWAS summary statistics for IgAN were obtained from a GWAS meta-analysis that included 10,146 kidney-biopsy-diagnosed IgAN cases and 28,751 controls from 17 international cohorts (12). Among these cohorts, 11 were of European ancestry, and six were of East Asian ancestry.

### Meta-analysis between pSSNS and IgAN

Inverse-variance weighted meta-analysis was performed on pSSNS and IgAN using the basic meta-analysis function in PLINK (v1.90b7) and the fixed-effect meta-analysis *P* value and fixed-effect ORs were estimated. We prioritized significant SNPs that reached genome-wide significance (*P* < 5×10^–8^) in the meta-analysis and suggestive significance (*P* < 0.05) in the original single-trait GWAS.

### Conjunctional FDR analysis between pSSNS and IgAN

To systematically evaluate shared genetic architecture between pSSNS and IgAN, we applied conjunctional false discovery rate (conjFDR) analysis (13, 14) as a complementary approach to conventional meta-analysis. Prior to this analysis, we assessed the genomic concordance between the pSSNS and IgAN GWAS datasets by evaluating the overlap of SNPs used in the analysis. A total of 8,553,764 SNPs were shared between the two datasets, accounting for approximately 85.4% of the pSSNS SNPs and 47.3% of the IgAN SNPs. This high degree of genomic coverage overlap ensures the validity of subsequent cross-trait analyses. First, conditional quantile-quantile (Q-Q) plots were generated to assess cross-trait polygenic enrichment by stratifying SNPs associated with one disorder (primary trait) based on their association strength with the other disorder (conditional trait) at progressively stringent *P*-value thresholds (*P* < 0.1, *P* < 0.01, *P* < 0.001). Distinct upward shifts in the conditional Q-Q curves of the primary trait with increasing significance levels of the conditional trait (i.e., progressive leftward displacement of the observed vs. expected –log10[*P*] distribution) provided evidence of genome-wide polygenic overlap.

The conjFDR framework extended this analysis by reciprocally conditioning the association statistics of pSSNS on IgAN and vice versa. For each SNP, the conjFDR metric was conservatively defined as the maximum of the two mutually conditioned FDR values derived from these bidirectional analyses. Significance thresholds were set at 0.01 for conjFDR. Pleiotropic loci exhibiting an LD r^2^ less than 0.1 with previously reported loci associated with pSSNS were categorized as novel, while others were considered known.

### GWAS analysis of Chinese individuals

A total of 501 pSSNS patients were recruited from Shandong Provincial Hospital Affiliated to Shandong First Medical University. The diagnosis of pSSNS was determined according to the criteria outlined in the KDIGO 2021 Clinical Practice Guideline for the Management of Glomerular Diseases (15). A total of 2,506 control subjects were selected from the CAS cohort based on questionnaire information indicating no history of pSSNS or other kidney diseases. The CAS cohort is a prospective multi-omics cohort comprising 3,197 employees (49.0%) from various institutes or offices of the Chinese Academy of Sciences in Beijing, China (16–19). This study was approved by the Institutional Review Boards of The Shandong Provincial Hospital of Shandong First Medical University, Beijing Institute of Genomics (Chinese Academy of Sciences).

Genotyping was conducted using the Infinium Asian Screening Array. Individuals with low genotype call rate (< 95%, n = 0), gender mismatch (n = 0), possible contamination (n = 0) or departure from Chinese Han population (n = 0) were removed before association test. SNPs were excluded if they were not on autosomal chromosomes, had a missing call rate ≥ 5%, had a minor allele frequency ≤ 1%, or had a Hardy-Weinberg equilibrium *P* value ≤ 1×10^-5^. After quality control, a total of 3,007 samples is left for further analyses. Imputation was done by Minimac3 using 1000 Genomes Project Phase 3 version 5 genotype data as reference. Multivariable logistic regression was employed to examine the association between genetic variants and the status of pSSNS diagnosis, utilizing PLINK 1.9 (20). The covariates included in the regression model were sex and the first five principal components (PCs).

### Differential expression analysis of candidate genes in pSSNS and IgAN

To investigate the differential expression of genes associated with novel loci identified in the meta-analysis and conjFDR analysis, we analyzed gene expression using the GSE145969 (21) and GSE93798 (22) datasets. For pSSNS, the GSE145969 dataset was utilized, which includes four groups: steroid-sensitive pre-treatment (n = 16), steroid-sensitive post-treatment (n = 16), steroid-resistant pre-treatment (n = 12), and steroid-resistant post-treatment (n = 12), with gene expression measured in peripheral blood leukocytes. Given the focus of this study on pSSNS, we specifically analyzed the differences in gene expression between pre-treatment and post-treatment groups within steroid-sensitive nephrotic syndrome patients. For IgAN, gene expression data were derived from the GSE93798 dataset, which includes 20 patients and 22 healthy controls. This dataset provides global transcriptomic data from the glomerular compartment of renal biopsies. Gene expression was compared between IgAN patients and healthy controls to identify differentially expressed genes near novel loci. Differential expression for all analyses was assessed using Wilcoxon rank-sum tests for candidate genes, with a significance threshold set at *P* < 0.01.

### Single-cell analysis of candidate genes in nephrotic syndrome

To investigate cell-type-specific dysregulation of novel genes prioritized by meta-analysis and conjFDR analysis, we analyzed a scRNA-seq dataset (GSE233277) comprising peripheral blood mononuclear cells (PBMCs) from four idiopathic nephrotic syndrome (INS) patients and four age/sex-matched healthy controls. Raw sequencing data preprocessed by the original study (23) were aligned to the GRCh38 reference genome and subjected to stringent quality control. Using the preprocessed count matrices, we applied the Seurat v5.2.1 pipeline with reciprocal PCA integration to correct batch effects, followed by UMAP-based clustering (35 principal components, resolution = 0.5) to identify major immune cell populations. We systematically evaluated cell-type-specific differential expression of the novel genes between INS patients and healthy controls using Wilcoxon rank-sum tests, analyzing both detection rate (proportion of expressing cells) and mean expression levels across all annotated immune populations. Given the exploratory nature of single-cell analysis across multiple cell types, statistical significance was initially defined as a nominal *P* < 0.05 without adjustment for multiple comparisons across the 18 identified cell clusters, to avoid masking potential biological signals in specific subpopulations.

## Results

### Meta-analysis between pSSNS and IgAN

**Figure 1:**
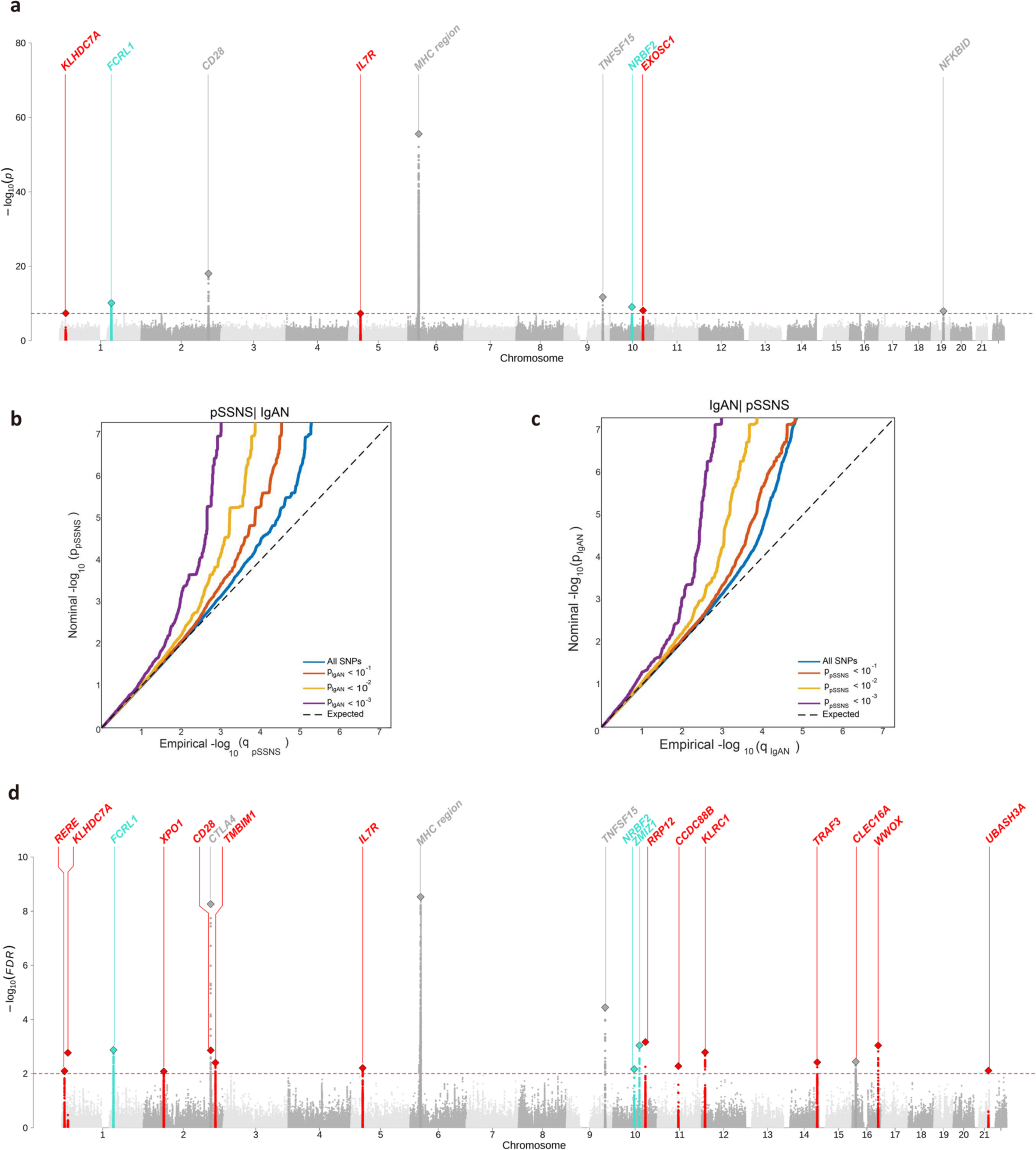
Manhattan plot and conditional quantile-quantile (Q-Q) plots of the meta-analysis and conjunctional FDR analysis between pSSNS and IgAN. **(a)** Manhattan plot of the meta-analysis; **(b)** The conditional quantile-quantile plot of nominal vs empirical −log10 p values for pSSNS as a function of significant association with IgAN at *P* value < 0.1, *P* value < 0.01, and *P* value < 0.001; **(c)** Reciprocal conditional Q-Q plot for IgAN, conditioned on pSSNS associations at matching *P*-value thresholds.; **(d)** Manhattan plot of the conjunctional FDR analysis. Newly identified loci with no prior GWAS associations in pSSNS or IgAN are shown in red, while loci that were previously reported in IgAN GWAS but newly identified in pSSNS are highlighted in blue.

We conducted a conventional meta-analysis by combining summary statistics derived from the GWAS of pSSNS and IgAN. Our analysis identified nine loci surpassing the significance threshold of *P* < 5 × 10^-8^ (**Table 1**, **Figure 1A**). Of these, rs75873622 (1p36.13), rs10213865 (5p13.2), and rs7895695 (10q24.1) are newly identified loci with no prior GWAS associations in pSSNS or IgAN. Two additional loci, rs3818813 (1q23.1) and rs57943165 (10q21.3) were newly genome-wide significant in pSSNS despite being previously reported in IgAN. The single-tissue eQTLs, single-cell eQTLs (sc-eQTLs), and DNA methylation QTLs (mQTLs) for these novel pleiotropic loci are presented in **Supplementary Tables 1**-**3**, respectively, derived from the Genotype-Tissue Expression (GTEx) Analysis Release V8, scQTLbase (24), and recent mQTL studies (25–27).

**Table 1:** Meta-analysis between pSSNS and IgAN.

CHR	SNP	BP	A1	A2	*P*-value (pSSNS)	*P*-value (IgAN)	*P*-value (meta)	Genes	Locus	Annotation
1	rs75873622	18766556	G	A	1.85E-04	6.33E-05	4.38E-08	*KLHDC7A*	1p36.13	new
1	rs3818813	157718325	G	T	4.61E-04	3.72E-08	7.53E-11	*FCRL1/FCRL2/* *FCRL3*	1q23.1	new*
2	rs3769684	204584759	C	T	2.77E-10	5.14E-11	9.33E-19	*CD28*	2q33.2	known
5	rs10213865	35857850	C	A	1.66E-03	6.57E-06	4.92E-08	*IL7R*	5p13.2	new
6	rs9275596	32681631	C	T	1.03E-26	3.39E-36	2.91E-56	MHC region	6p21.32-6p21.33	known
9	rs6478108	117558703	C	T	2.10E-10	3.47E-06	1.89E-12	*TNFSF15*	9q32	known
10	rs57943165	65362966	G	T	4.32E-03	1.10E-08	8.97E-10	*NRBF2*	10q21.3	new*
10	rs7895695	99167255	G	A	1.89E-06	7.72E-05	7.80E-09	*EXOSC1/FAT/* *RRP12/ARHGAP19*	10q24.1	new
19	rs8113704	36387881	G	A	5.38E-11	0.047	1.15E-08	*NFKBID*	19q13.12	known

CHR, Chromosome number.

SNP, Reference SNP identifier (rsID).

BP, Base pair position (GRCh37).

A1, Effect allele.

A2, Non-effect allele.

*P*-value (pSSNS), Association p-value in pediatric steroid-sensitive nephrotic syndrome (pSSNS) GWAS.

*P*-value (IgAN), Association p-value in IgA nephropathy (IgAN) GWAS.

*P*-value (meta), Meta-analysis p-value combining pSSNS and IgAN results.

Genes, Nearest or implicated gene(s).

Locus, Cytogenetic band location.

Annotation:.

- new, newly identified loci with no prior GWAS associations in pSSNS or IgAN.

- new*, previously reported in IgAN GWAS but newly identified in pSSNS.

- known, established loci reported in both pSSNS and IgAN GWAS.

Notably, rs3818813 (1q23.1) and rs10213865 (5p13.2) exhibited convergent multi-omics regulatory mechanisms for nearby genes. Specifically, rs3818813 functioned as an eQTL for *FCRL1* and *FCRL3* in whole blood and demonstrated cell-type-specific expression regulation (sc-eQTLs) for *FCRL1*, *FCRL2* and *FCRL3* in CD4^+^ T cells, CD8^+^ T cells, natural killer cells, and B cells. This locus further displayed multiple mQTL associations, with the strongest signal observed at probe cg21721331 near *FCRL3* (*P* = 3.66 ×10^-116^). Similarly, rs10213865 (5p13.2) emerged as an sc-eQTL for *IL7R* in CD4^+^ T cells, CD8^+^ naive T cells, and monocytes, with complementary mQTL evidence linking it to two *IL7R*-proximal CpG sites (cg27582180: *P* = 1.08 × 10^-8^; cg04312209: *P* = 1.71 × 10^-9^).

### Conjunctional FDR analysis between pSSNS and IgAN

We observed enrichment in genetic association with pSSNS as a function of the significance of association with IgAN, as indicated by the successive leftward deflection in the pSSNS Q-Q plot as the P value for IgAN decreases (**Figure 1B**). This pattern suggests that variants strongly associated with IgAN are more likely to show association signals in pSSNS, reflecting a shared genetic basis between the two diseases. A similar pattern was observed in the reverse direction: SNPs passing more stringent *P* value thresholds for pSSNS generally demonstrated stronger associations with IgAN (**Figure 1C**).

We next implemented conjFDR analysis to further investigate the shared genetic architecture between pSSNS and IgAN. At a conjFDR threshold of <0.01, we identified 19 loci exhibiting pleiotropic associations with both diseases (**Table 2**, **Figure 1D**). Consistent with the conventional inverse variance-weighted meta-analysis, five novel loci—located at 1p36.13, 1q23.1, 5p13.2, 10q21.3, and 10q24.1—were also detected in the conjFDR analysis. In addition to these, conjFDR identified ten further loci, including nine novel associations with no prior GWAS evidence for either disease and one locus, rs1250564 (10q22.3), previously reported in IgAN, that achieved genome-wide significance in pSSNS for the first time. Notably, the lead SNPs at the significant loci (rs1763839, rs3771258, rs13429408, rs694739, rs12826560, rs6575931 and rs1893592) were identified as eQTLs for nearby genes across multiple tissues (**Supplementary Table 1**). For example, rs12826560 functions as an eQTL for the nearby genes *KLRC1*, *KLRC2* and *KLRC3* in whole blood, spleen and thyroid (**Supplementary Table 1**). Furthermore, rs12826560 was found to influence the expression of nearby genes in various immune cells. Specifically, rs12826560 modulates the expression of *KLRC1*, *KLRC2* and *KLRC3* in several immune cell types, including natural killer (NK) cells, naive B cells, memory B cells, CD4^+^ T cells, and CD8^+^ T cells (**Supplementary Table 2**). In addition to the eQTL evidence, mQTL results further reveal that rs12826560 is also associated with DNA methylation levels, suggesting that the regulation of nearby gene expression by these SNPs may be influenced by epigenetic modifications. For example, rs12826560 showed associations with probe cg08041188 near genes *KLRK1*, *KLRC1*, *KLRC2*, *KLRC3*, *KLRC4* (*P* = 1.40×10^-59^) (**Supplementary Table 3**).

**Table 2:** Conjunctional FDR analysis between pSSNS and IgAN.

CHR	SNP	BP	A1	A2	*P*-value (pSSNS)	*P*-value (IgAN)	*P*-value (conjFDR)	Gene	Locus	Annotation
1	rs1763839	8473336	G	A	1.03E-03	7.70E-04	0.008	*RERE*	1p36.23	new
1	rs75873622	18766556	G	A	1.85E-04	6.33E-05	0.002	*KLHDC7A/IGSF21*	1p36.13	new
1	rs7524764	157685739	G	A	1.40E-04	2.72E-07	0.001	*FCRL1/FCRL2/FCRL3*	1q23.1	new*
2	rs3771258	61764140	C	T	5.41E-04	1.40E-03	0.008	*XPO1*	2p15	new
2	rs3769684	204584759	C	T	2.77E-10	5.14E-11	5.48E-09	*CD28*	2q33.2	known
2	rs231805	204708749	G	A	1.45E-04	5.01E-05	0.001	*CTLA4*	2q33.2	new
2	rs13429408	219142860	C	A	4.67E-04	2.07E-04	0.004	*TMBIM1/PNKD*	2q35	new
5	rs34463936	35850149	C	T	7.63E-04	5.94E-05	0.006	*IL7R*	5p13.2	new
6	rs2857697	31585219	C	T	5.73E-17	1.59E-11	2.97E-09	*MHC region*	6p21.3	known
9	rs3810936	117552885	C	T	1.80E-08	2.94E-06	3.58E-05	*TNFSF15*	9q32	known
10	rs77973332	64881009	C	A	8.47E-04	4.32E-05	0.007	*NRBF2/JMJD1C*	10q21.3	new*
10	rs1250564	81047342	C	A	8.98E-05	1.16E-08	9.10E-04	*ZMIZ1/PPIF*	10q22.3	new*
10	rs7895695	99167255	G	A	1.89E-06	7.72E-05	6.82E-04	*RRP12/EXOSC1*	10q24.1	new
11	rs694739	64097233	G	A	4.31E-04	8.43E-04	0.005	*CCDC88B*	11q13.1	new
12	rs12826560	10532965	C	T	1.20E-04	2.15E-04	0.002	*KLRC1/KLRC2/* *KLRC3/KLRC4/KLRK1*	12p13.2	new
14	rs6575931	103301574	G	A	4.47E-04	1.36E-05	0.004	*TRAF3*	14q32.32	new
16	rs7206753	11068844	C	T	3.12E-04	5.55E-04	0.004	*CLEC16A*	16p13.13	known
16	rs36001636	79349806	C	T	9.04E-05	8.09E-05	9.14E-04	*MAF/WWOX*	16q23.2	new
21	rs1893592	43855067	C	A	1.95E-04	1.30E-03	0.008	*UBASH3A*	21q22.3	new

CHR, Chromosome number.

SNP, Reference SNP identifier (rsID).

BP, Base pair position (GRCh37).

A1, Effect allele.

A2, Non-effect allele.

*P*-value (pSSNS), Association p-value in pediatric steroid-sensitive nephrotic syndrome (pSSNS) GWAS.

*P*-value (IgAN), Association p-value in IgA nephropathy (IgAN) GWAS.

*P*-value (meta), Meta-analysis p-value combining pSSNS and IgAN results.

*P*-value (conj FDR), Conjunctional false discovery rate p-value across pSSNS and IgAN.

Genes, Nearest or implicated gene(s).

Locus, Cytogenetic band location.

Annotation:.

- new, Newly identified loci with no prior GWAS associations in pSSNS or IgAN.

- new*, Previously reported in IgAN GWAS but newly identified in pSSNS.

- known, Established loci reported in both pSSNS and IgAN GWAS.

### Replication of pleiotropic loci in the Chinese population

**Figure 2:**
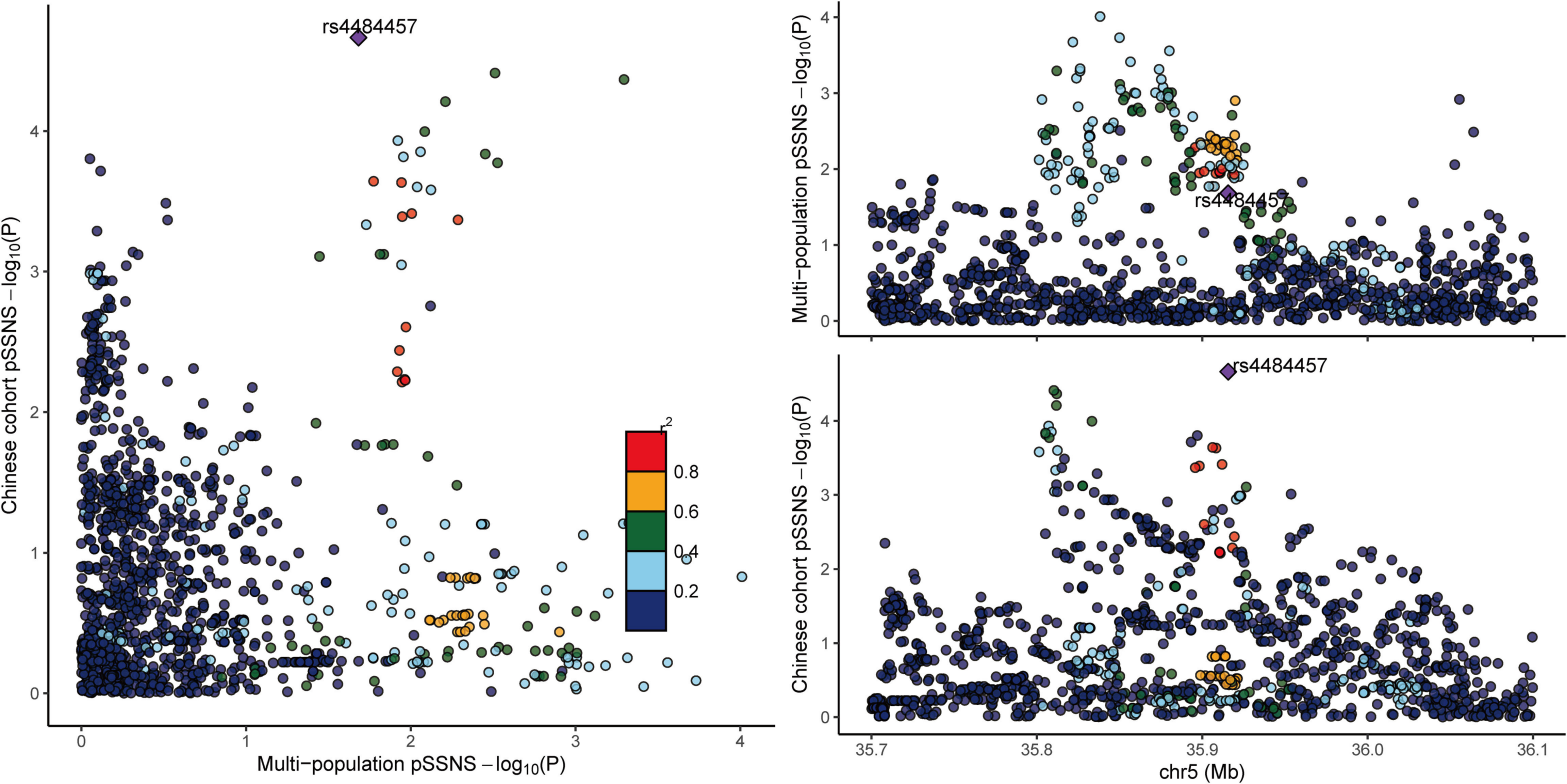
Co-localization between publicly available multi-population pSSNS GWAS and the Chinese-cohort pSSNS GWAS at the 5p13.2 locus. The left panel displays the merged association plot for the multi-population pSSNS GWAS and the Chinese cohort pSSNS GWAS. The right panel shows the regional plots for both the multi-population and Chinese pSSNS GWAS, with points colored by LD with respect to rs4484457, which is labeled with a purple diamond.

To validate the novel loci identified in the prior meta-analysis and conjFDR analysis, we performed a GWAS study in a Chinese cohort comprising 501 pSSNS patients and 2,506 healthy controls, the clinical characteristics of the study cohort are summarized in **Supplementary Table 4**. We first assessed the significance of the eight previously reported risk loci. As shown in **Supplementary Table 5**, seven of the eight loci were significantly associated with pSSNS (*P* < 0.05), supporting the reliability of our GWAS analysis in the Chinese population. Regarding the pleiotropic loci identified in the cross-trait analysis between pSSNS and IgAN, this Chinese cohort GWAS successfully replicated three previously established loci and one novel pleiotropic locus at 16q23.2 (rs12596357, *P* = 0.002), which were implicated in the conjFDR analysis (**Supplementary Table 6**; *P* < 0.05). Notably, the 5p13.2 locus around *IL7R*, marked by rs10213865, was detected in both the meta-analysis and conjFDR analysis. Although rs10213865 did not achieve significance in the Chinese cohort, we identified a nearby proxy variant, rs4484457 in moderate linkage disequilibrium (r² = 0.42) with rs10213865, which showed significant association in the Chinese population (*P* = 2.16×10^-5^). This suggests that while the exact lead variant may differ across populations due to allele frequency differences and linkage disequilibrium patterns, the genetic signal at this locus is reproducible (**Figure 2**).

### Differential gene expression analysis of *IL7R* in pSSNS and IgAN

**Figure 3:**
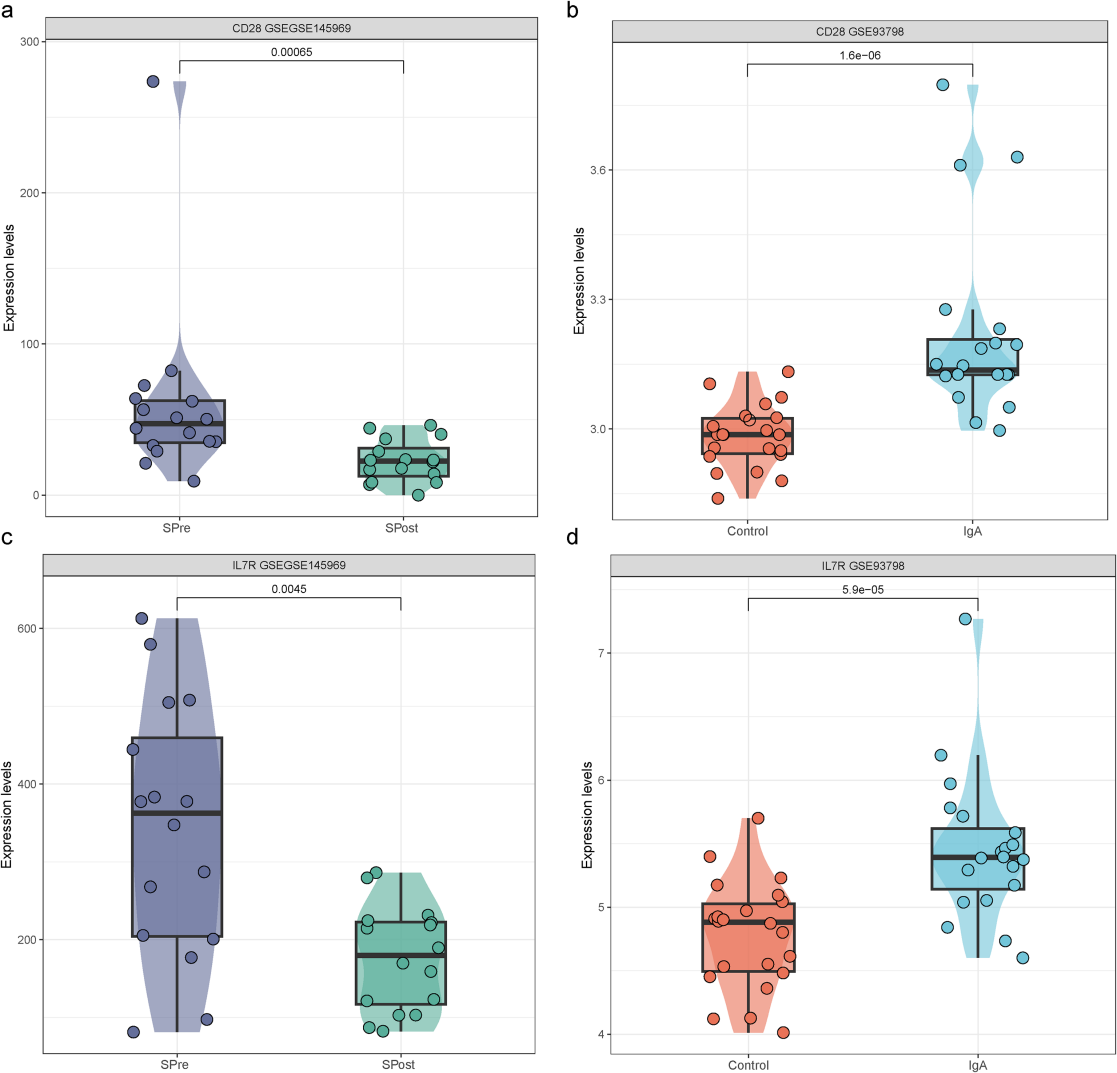
Differential Gene Expression of Pleiotropic Loci in pSSNS and IgAN. Violin and box plots show gene expression levels from RNA sequencing data. Blue represents the IgAN patient group, red represents the control group, purple represents the pSSNS group before steroid treatment (SPre), and green represents the pSSNS group after steroid treatment (SPost). **(a)** CD28 expression in pSSNS (SPre vs SPost); **(b)** CD28 expression in IgAN (vs control); **(c)** IL7R expression in pSSNS (SPre vs SPost); **(d)** IL7R expression in IgAN (vs control). Statistical significance (*P*-values) for group comparisons are shown above the plots.

We analyzed RNA sequencing data from publicly available datasets to explore the differential expression of genes associated with pSSNS and IgAN. The datasets included gene expression profiles from peripheral blood leukocytes and renal biopsy samples, which were downloaded from the GEO database. We first investigated the expression patterns of candidate genes at previously reported loci associated with both pSSNS (8) and IgAN (12). Among them, *CD28* was significantly differentially expressed in steroid-resistant patients before vs after treatment (**Figure 3A**, *P* = 0.00065) and in patients with IgAN vs healthy controls (**Figure 3B**, *P* = 1.60×10^-6^). These findings further support the role of *CD28* in the pathogenesis of pSSNS and IgAN. Next, we analyzed genes located near the novel loci identified in our study. Remarkably, the *IL7R* gene at the 5p13.2 locus exhibited a differential expression profile resembling that of *CD28*, showing significant downregulation in the steroid-sensitive group after treatment compared to before treatment (**Figure 3C**, *P* = 0.0045) and significant upregulation in IgAN patients compared to healthy controls (**Figure 3D**, *P* = 5.90×10^-5^), suggesting its potential critical role in both pSSNS and IgAN. Additionally, several genes near other novel loci demonstrated significant differential expression in IgAN patients. For example, *FCRL3* at the 1q23.1 locus (*P* = 0.0.002) and *KLRC3* at the 12p13.2 locus (*P* = 0.0064) were significantly upregulated, whereas *JMJD1C* at the 10q21.3 locus (*P* = 8.48×10^-7^) and *WWOX* at the 16q23.2 locus (*P* = 0.00033) were significantly downregulated. These findings suggest the potential involvement of these genes in the pathogenesis of IgAN (**Supplementary Table 7**).

### Comparative single-cell analysis of *IL7R* dysregulation in nephrotic syndrome

**Figure 4:**
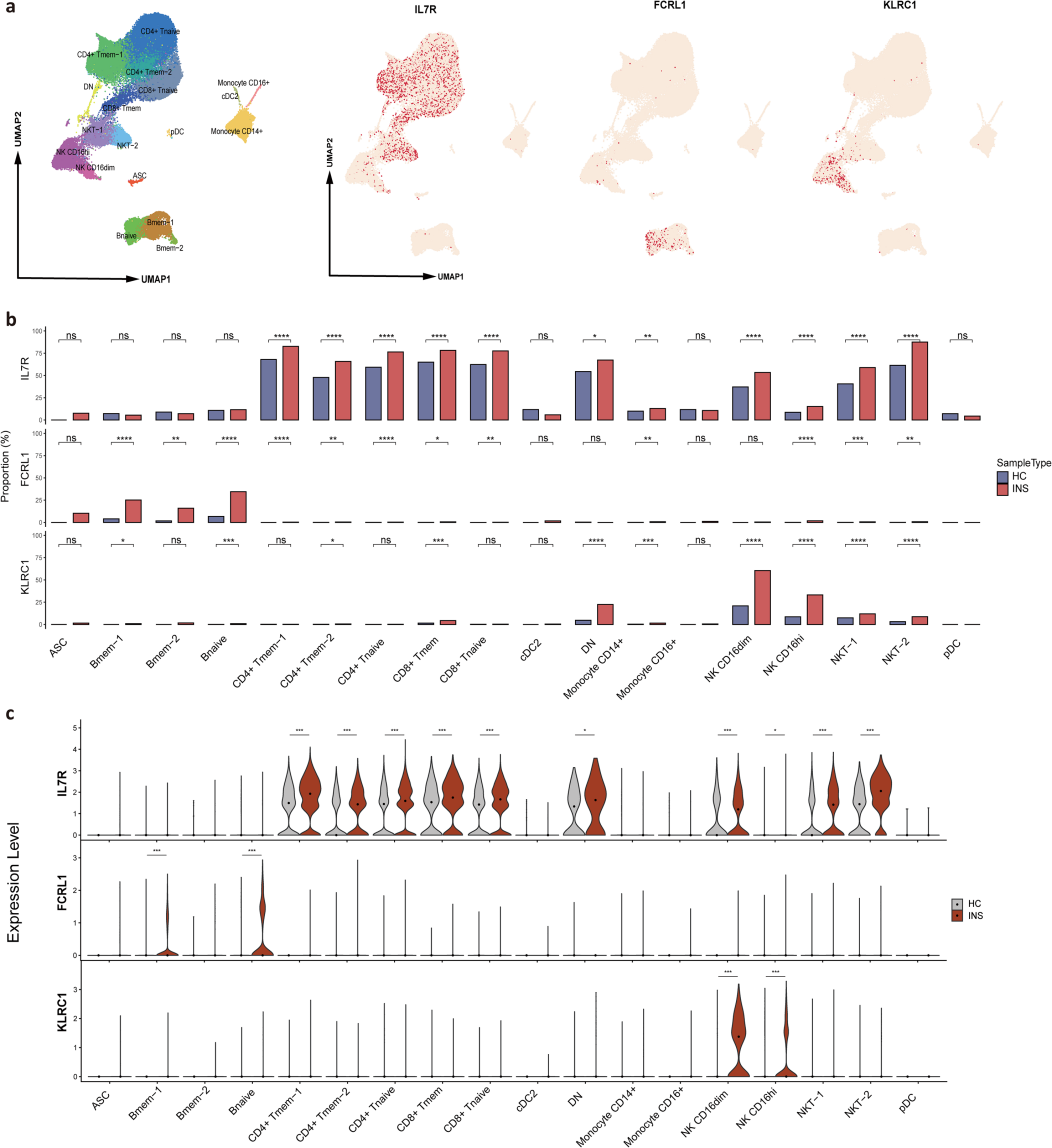
Single-nuclei transcriptomes of novel candidate genes in nephrotic syndrome pathogenesis. **(a)** Left: Uniform Manifold Approximation and Projection (UMAP) plot showing discrete cell-specific clusters based on single-nucleus transcriptomes; Right: expression patterns of *IL7R*, *FCRL1*, and *KLRC1* across cell clusters, with red indicating higher expression levels. **(b)** Proportions of *IL7R*-, *FCRL1*-, and *KLRC1*-expressing cells within immune subclusters in healthy children (HC, blue) and idiopathic nephrotic syndrome patients (INS, red). **(c)** Expression levels of *IL7R*, *FCRL1*, and *KLRC1* within immune subclusters in HC (gray) and INS (red). *P*-values were calculated using the Wilcoxon rank-sum test: ****P < 1e-4; ***P < 0.001; **P < 0.01; * *P* < 0.05; ns, not significant.

To investigate cell-type-specific expression patterns of *IL7R* and other detected candidate genes in nephrotic syndrome pathogenesis, we profiled a single-cell RNA sequencing (scRNA-seq) dataset (GSE233277) encompassing peripheral blood mononuclear cells (PBMCs) from 4 INS patients and 4 healthy controls. After stringent quality filtering, 69,994 high-confidence immune cells were subjected to unsupervised clustering, resolving 18 transcriptionally distinct immune cell clusters (**Figure 4A**). Single-cell resolution analysis validated the critical involvement of *IL7R* in INS pathology, demonstrating conserved overexpression patterns across adaptive immune lineages (CD4^+^ T, CD8^+^ T, NK cells) (**Figure 4A**) that aligned with prior bulk tissue observations. Specifically, in CD4^+^ Tmem-1 cells, *IL7R* was detected in 82.64% of patient cells versus 67.96% controls (*P* = 8.44×10^−58^) (**Figure 4B**), with concomitant expression elevation (*P* = 4.95×10^−99^) (**Figure 4C**). The CD8^+^ Tnaive compartment recapitulated this dysregulation, showing 77.57% *IL7R* detection in patients compared to 62.37% controls (*P* = 2.29×10^−54^) (**Figure 4B**) and heightened transcriptional activity (P = 3.07×10^−50^) (**Figure 4C**). Among NK populations, this conserved pattern peaked in NKT-2 cells, where 87.54% of patient cells expressed *IL7R* (vs. 61.42% controls; *P* = 1.18×10^−41^) alongside robust upregulation (P = 5.73×10^−63^).

In addition to the *IL7R* locus, the 1q23.1 locus encompasses multiple Fc Receptor-like (FCRL) genes, including *FCRL1*, *FCRL2*, and *FCRL3*, which are predominantly expressed in B cells. For example, FCRL1 exhibited a B cell-restricted expression profile, with striking enrichment in B naive subsets from INS patients (34.52% vs. 6.70% controls; *P* = 3.51×10^−45^). Similarly, the 12p13.2 locus harbors several NK cell–related genes, including *KLRC1*, *KLRC2*, *KLRC3*, *KLRC4*, and *KLRK1*, all of which show predominant expression in NK cells. Notably, *KLRC1* demonstrated strict NK cell specificity, localizing mainly to CD16^dim and CD16^hi subpopulations. *KLRC1*-expressing cells were significantly elevated in patients, reaching 22.43% in CD16^dim NK cells (vs. 4.72% in controls; *P* = 1.38×10^−8^) and 33.15% in CD16^hi subsets (vs. 8.64% in controls; *P* = 7.32×10^−107^). Single-cell gene expression results for additional candidate genes are provided in **Supplementary Figures 2**–**S4**.

## Discussion

In this study, we conducted a cross-trait genome-wide association analysis to investigate the genetic architecture of pSSNS and its potential overlap with IgAN. Using meta-analysis and conjFDR methods, we identified novel genetic loci associated with pSSNS, providing new insights into its underlying susceptibility factors. Several of these loci were further validated in an independent Chinese cohort, supporting their relevance in pSSNS across populations. Additionally, transcriptomic analyses suggested potential regulatory roles for these loci, offering functional evidence for their involvement in disease pathogenesis.

The potential genetic overlap pSSNS and IgAN is of particular interest given the immune-mediated nature of both diseases. While pSSNS is primarily characterized by podocyte dysfunction and a T-cell–driven immune response, IgAN involves aberrant mucosal immunity and immune complex deposition in the glomeruli. Previous GWASs have suggested that certain immune-related loci, particularly within the HLA region and cytokine signaling pathways, may contribute to susceptibility to both conditions (8, 12). Given these shared immunogenetic features, a cross-trait analysis can provide insight into common genetic risk factors that may underlie immune dysregulation in both diseases.

One notable finding in this study is the association of the *IL7R* locus at 5p13.2 with pSSNS, identified through both meta-analysis and conjFDR analysis. *IL7R* encodes the interleukin-7 receptor, a key regulator of T-cell development and homeostasis, which plays a crucial role in adaptive immunity. Dysregulation of *IL7R* signaling has been implicated in various autoimmune and inflammatory conditions, suggesting a potential mechanism through which this locus may contribute to pSSNS susceptibility (28–30). Although the lead SNP rs10213865 did not reach statistical significance in the Chinese replication cohort, we identified a nearby proxy variant, rs4484457, which is in moderate linkage disequilibrium (r² = 0.42) with rs10213865 and demonstrated a significant association (*P* = 2.16 × 10^-5^) in the Chinese population. This suggests that while the specific risk variant may differ across populations, the genetic signal at this locus is reproducible. Such discrepancies could be attributed to differences in allele frequencies, linkage disequilibrium structure, or statistical power, suggesting the importance of population-specific fine-mapping. Moreover, rs10213865 has been identified as an eQTL, sc-eQTL, and mQTL, indicating that it influences *IL7R* expression across multiple tissues and immune cell types. Single-cell eQTL (sc-eQTL) data suggest that this variant modulates *IL7R* expression in key immune cell populations, while mQTL findings indicate potential epigenetic regulation at this locus. These multi-layered regulatory effects strengthen the biological plausibility of *IL7R* involvement in pSSNS pathogenesis. Our transcriptomic analysis further demonstrated that *IL7R* expression was significantly altered in pSSNS patients before and after corticosteroid treatment, reinforcing its potential relevance in disease pathogenesis. The observed changes suggest that *IL7R* may play a role in the immunomodulatory response to steroid therapy, potentially influencing disease progression and treatment outcomes. Given the immune-mediated nature of both pSSNS and IgAN, broader adaptive immune activation may contribute not only to disease susceptibility but also to the persistence of inflammatory activity. Immunosuppressive therapy, particularly corticosteroids, may partially attenuate these processes through modulation of T-cell activation and cytokine signaling pathways.

Importantly, our scRNA-seq analysis provided additional resolution into the cellular context of *IL7R* dysregulation, complementing the bulk RNA-seq findings. We observed *IL7R* overexpression specifically in CD4^+^ T cells, CD8^+^ T cells, and NK cells, suggesting its broad involvement across immune compartments in nephrotic syndrome. While the previous study by Al-Aubodah et al. (23) primarily focused on B cell alterations due to their significant abundance differences between cases and controls, our reanalysis of the same scRNA-seq dataset, guided by cross-trait GWAS findings, unveiled novel insights into T cell biology. Although the overall proportion of T cells did not significantly differ between cases and controls, we observed a notable increase in both the proportion of *IL7R*-expressing T cells and the *IL7R* expression levels within these cells in cases. This pattern suggests a layer of regulatory complexity beyond simple shifts in cell composition and highlights potential functional dysregulation within T cell populations. Notably, given the exploratory nature of single-cell analysis across multiple cell types, these differential expression findings were evaluated using nominal P-value thresholds without adjustment for multiple comparisons across the 18 identified cell clusters; however, the remarkably low *P*-values observed for key candidates such as *IL7R* indicate that these results are robust and unlikely to be confounded by multiple testing.​ These findings further support *IL7R* as a strong candidate gene for pSSNS and indicate the importance of integrating genetic association data with single-cell transcriptomics to uncover subtle yet significant alterations in immune cell function that may contribute to disease pathogenesis. Future studies integrating single-cell and spatial transcriptomic approaches with renal histopathology may further elucidate the spatial localization and functional states of infiltrating immune cells in affected kidney tissues.

Beyond disease susceptibility, the identified loci may also have potential clinical utility as biomarkers for disease monitoring and risk stratification. In particular, *IL7R* showed convergent evidence across GWAS, eQTL, sc-eQTL, mQTL, and transcriptomic analyses, highlighting its potential as a robust molecular candidate reflecting immune dysregulation in pSSNS. These results suggest that immune-related molecular signatures may facilitate improved longitudinal monitoring of immune-mediated renal diseases. Future studies integrating genetic, transcriptomic, and immune cell–specific biomarkers may further improve individualized disease assessment and therapeutic monitoring.

This study has several limitations. First, while the cross-trait GWAS analysis identified novel susceptibility loci, additional functional validation is required to establish their causal roles in pSSNS pathogenesis. Second, although transcriptomic and single-cell analyses provided preliminary insights into the functional relevance of candidate genes, these datasets were primarily derived from peripheral blood rather than kidney tissue. While peripheral immune profiling captures systemic immune dysregulation associated with pSSNS and IgAN, it may not fully reflect organ-specific transcriptional alterations within the renal microenvironment. In addition, further experimental studies are required to confirm the biological significance of the identified candidate genes and their functional roles in renal pathology. Future studies integrating renal biopsy-derived bulk and single-cell transcriptomic datasets, together with spatial transcriptomic approaches, will be important to further define the tissue-specific expression patterns and cellular localization of candidate genes such as *IL7R.* Third, the modest sample size of the Chinese replication cohort limited statistical power to detect loci with smaller effect sizes. This limitation may partly explain why some discovery-phase signals, such as the *IL7R* lead SNP (rs10213865), showed consistent directionality but did not reach genome-wide significance in the replication analysis. Therefore, these non-significant findings should be interpreted with caution, as they may reflect limited statistical power rather than true absence of association. Future studies with larger, more diverse cohorts will be essential to further validate these findings and refine population-specific genetic risk factors.

Taken together, this study provides new insights into the genetic architecture of pSSNS, identifying novel susceptibility loci and their potential regulatory roles. The replication of previously reported loci in the Chinese population supports the robustness of these findings. Future studies with multi-ancestry cohorts and functional analyses are needed to refine causal.

variants and conclusively inform potential therapeutic strategies.

## Data Availability

The original contributions presented in the study are included in the article/**Supplementary Material**, further inquiries can be directed to the corresponding authors.
