# Supplementary material for: Identification of IL7R as a key genetic risk locus in childhood steroid-sensitive nephrotic syndrome and IgA nephropathy
Source: Front Immunol. 2026 May 29;17:1806680. doi: 10.3389/fimmu.2026.1806680 (PMC13260247; doi:10.3389/fimmu.2026.1806680)

Supplementary Figure 1. PRISMA-style flowchart illustrating the multi-stage integration of GWAS, cross-trait analyses, and multi-omics validation in childhood SSNS and IgA nephropathy.


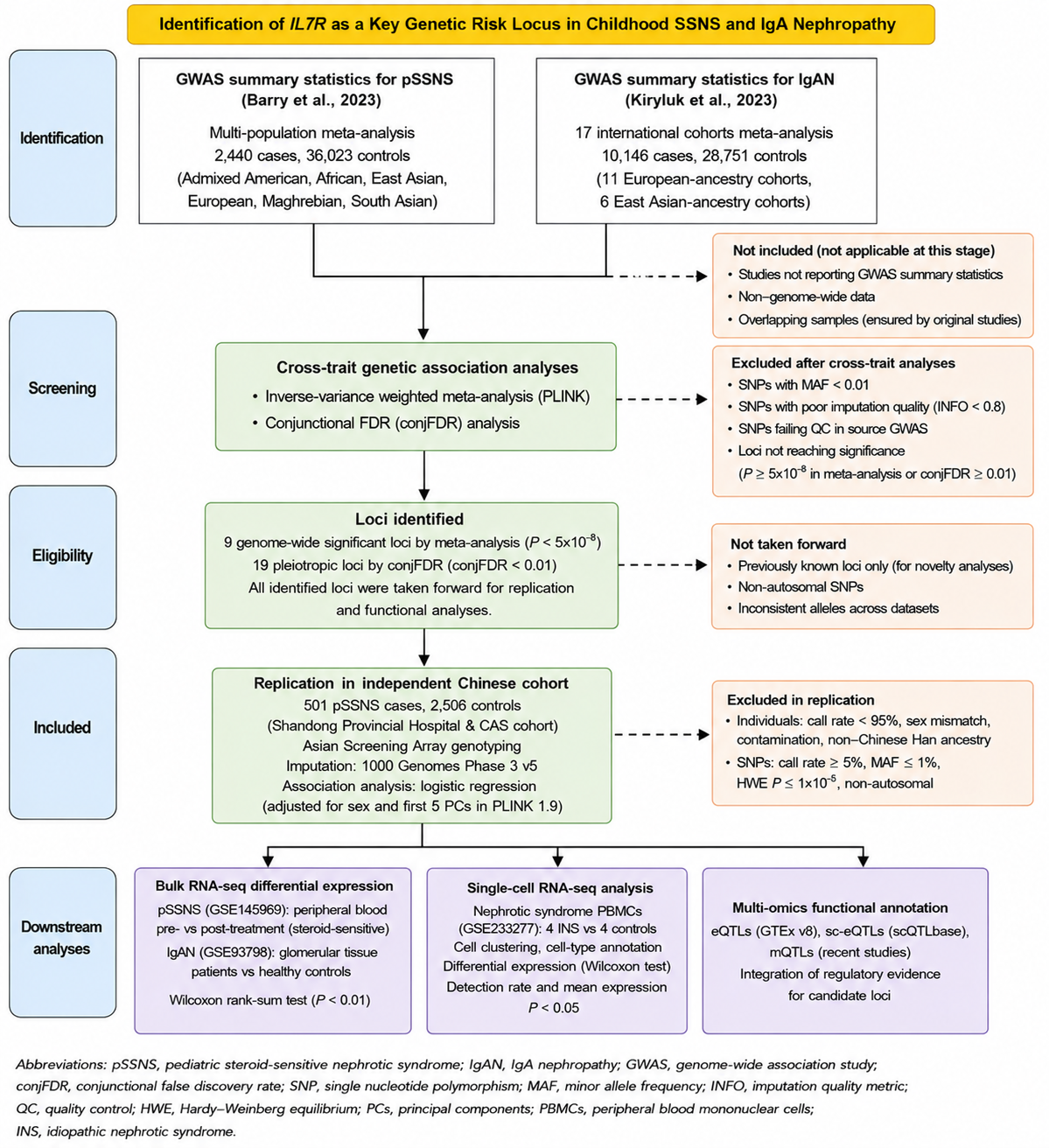


Supplementary Figure 2. Expression of candidate genes across cell-specific clusters from UMAP, grouped by annotation type. Red indicates higher expression. A, genes newly identified in this study with no prior GWAS associations in pSSNS or IgAN; B, genes previously reported in IgAN GWAS but newly associated with pSSNS; C, known genes reported in both pSSNS and IgAN GWAS.

A)


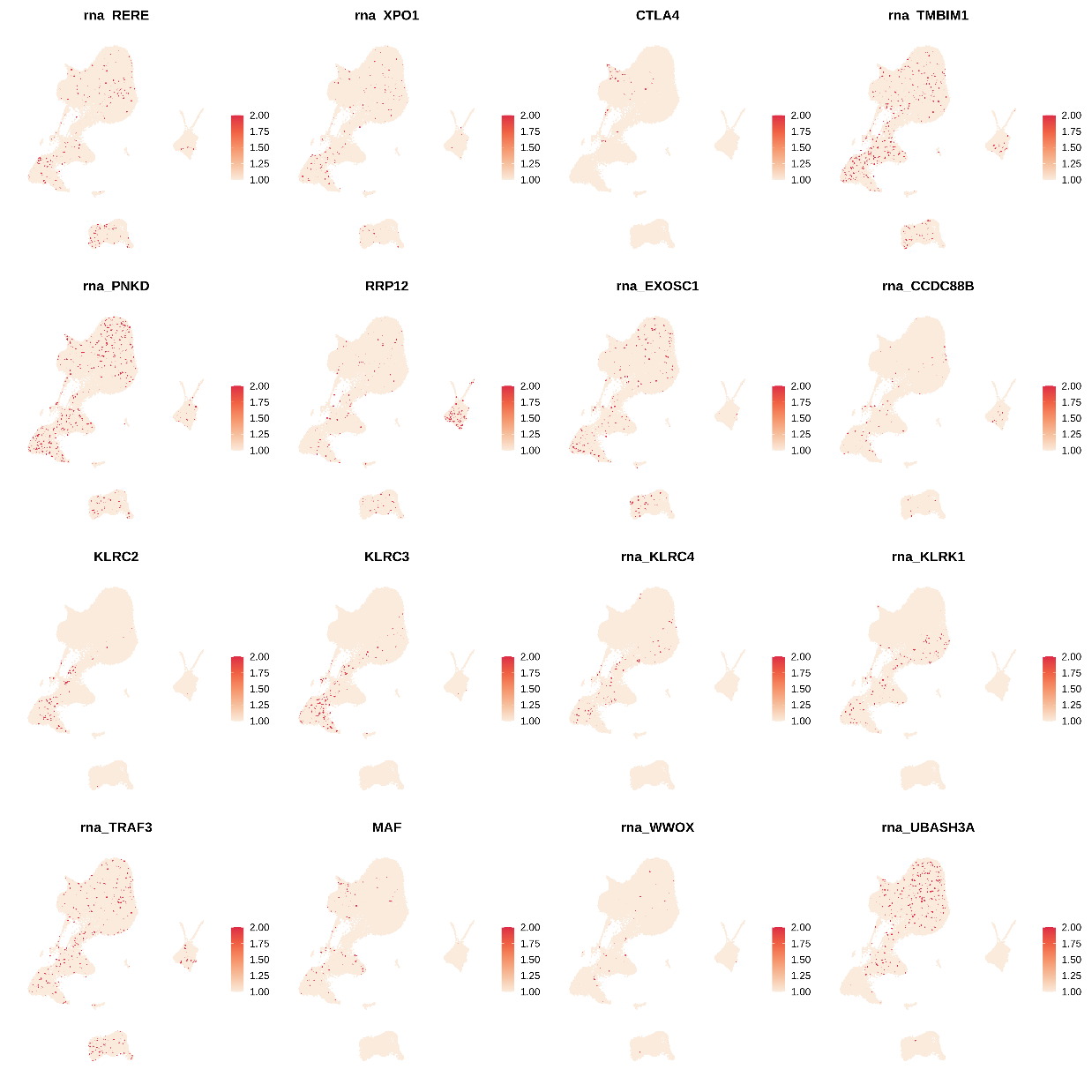


B)


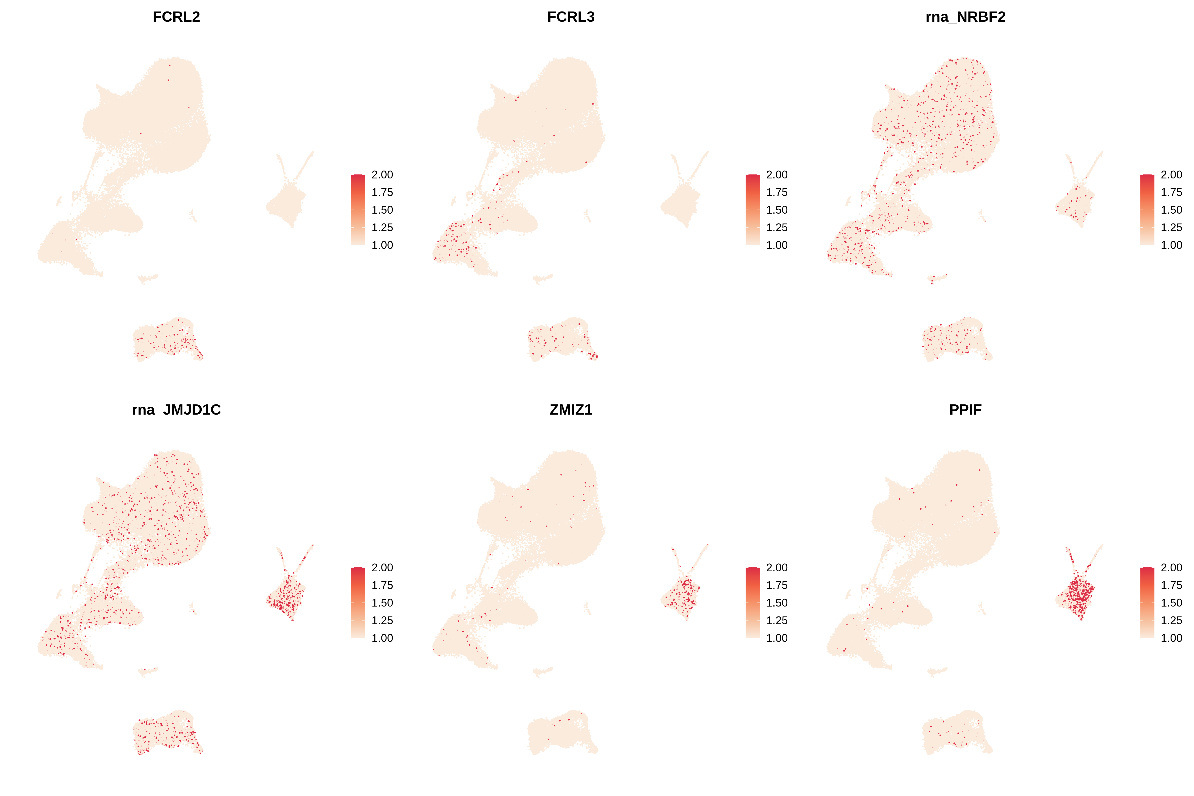


C)


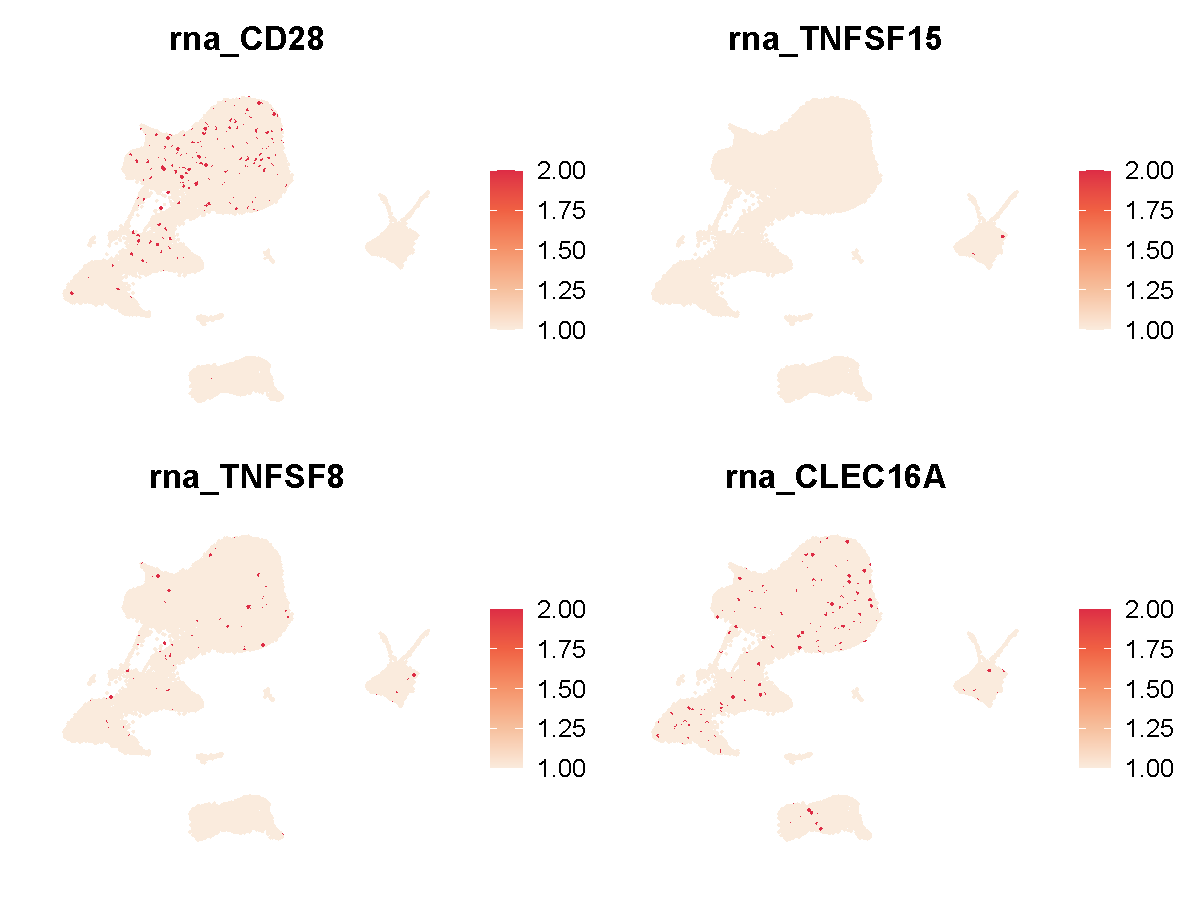


Supplementary Figure 3. Proportions of candidate genes-expressing cells within respective immune subclusters in healthy children (HC, blue) and idiopathic nephrotic syndrome patients (INS, red), grouped by annotation type. A, genes newly identified in this study with no prior GWAS associations in pSSNS or IgAN; B, genes previously reported in IgAN GWAS but newly associated with pSSNS; C, known genes reported in both pSSNS and IgAN GWAS. P-values were determined using Wilcoxon rank-sum tests: ****, p < 1e-4; ***, p<0.001; **, p<0.01; *, p<0.05; ns, not significant.

A)


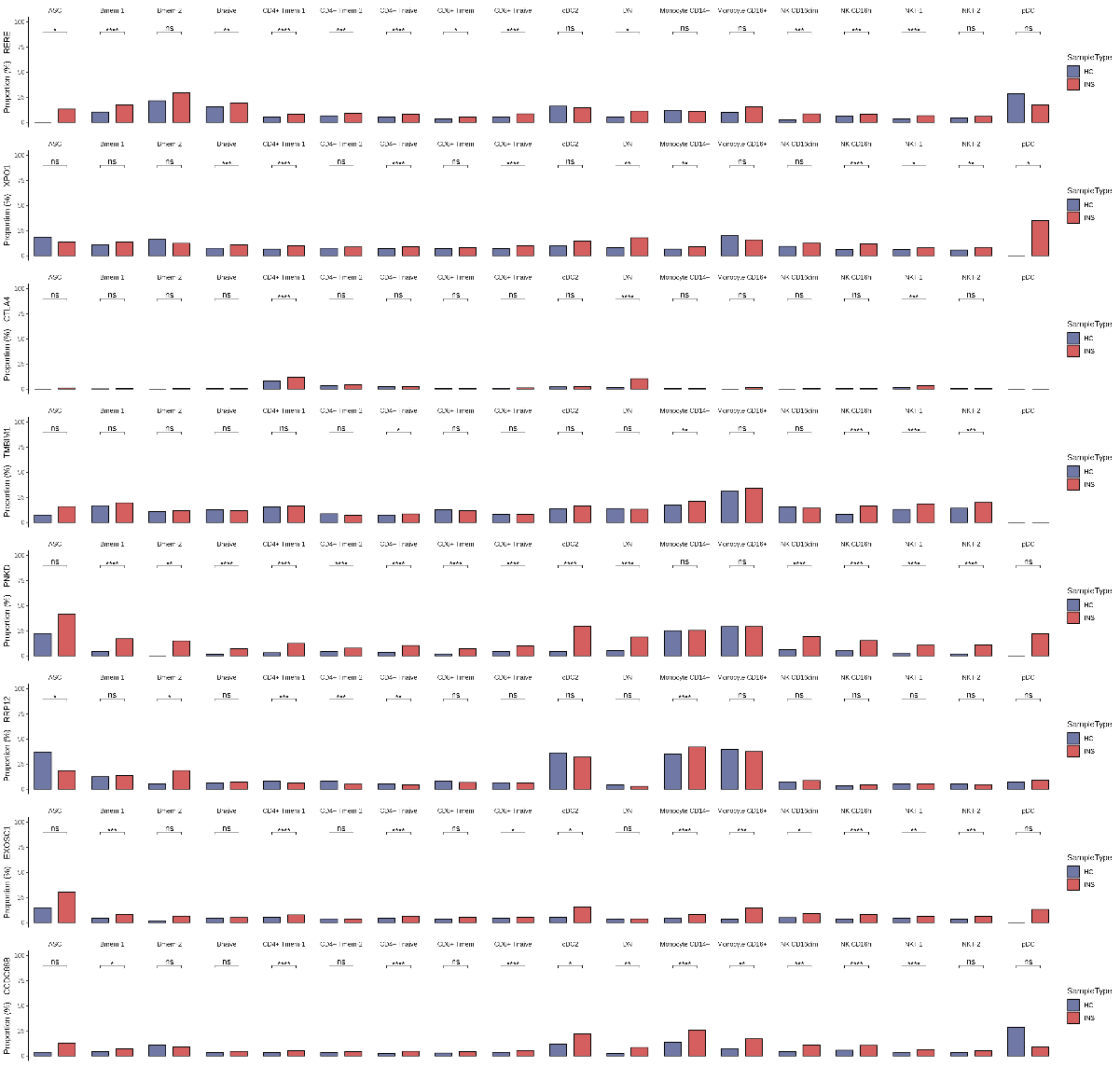


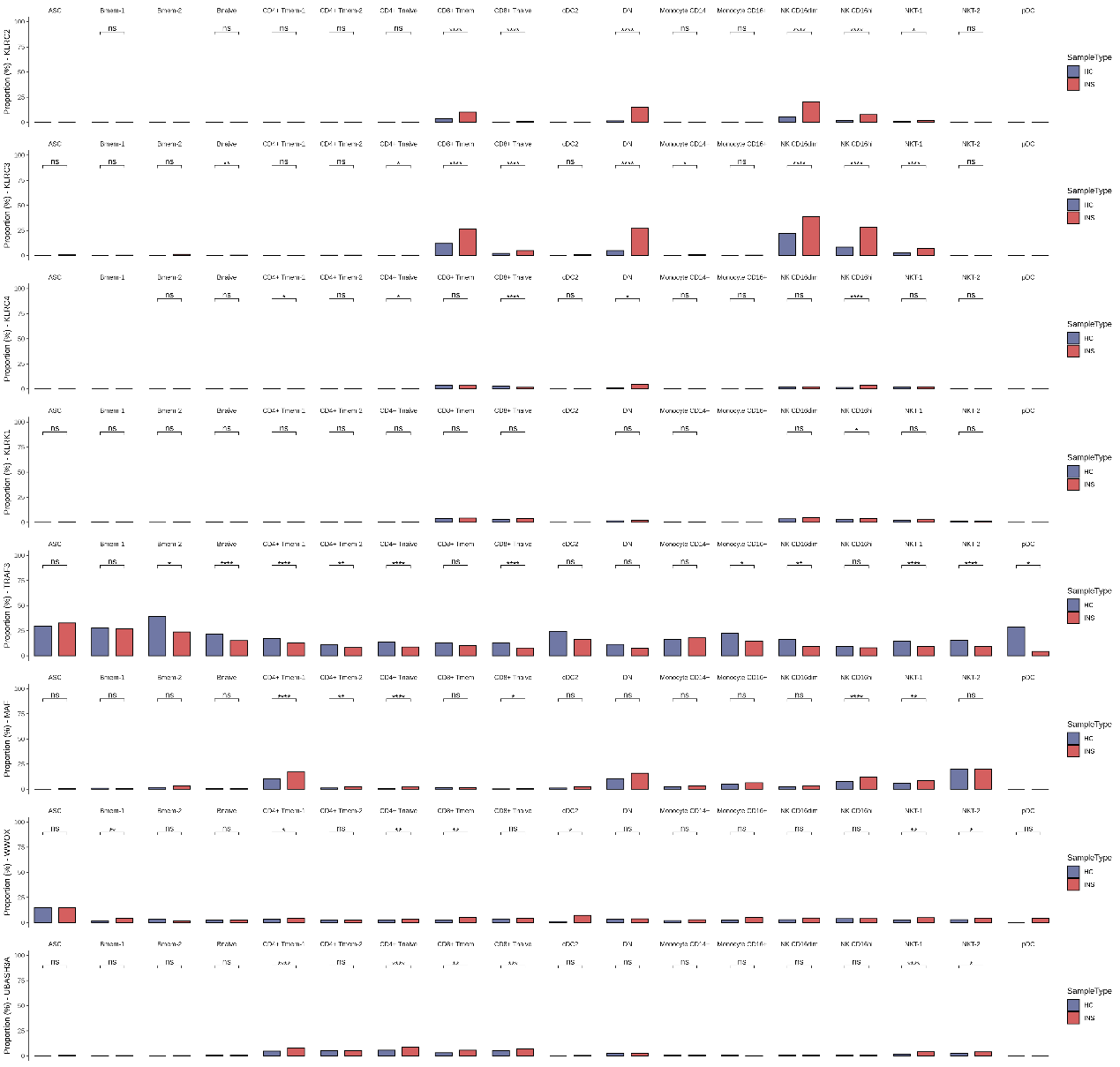


B)


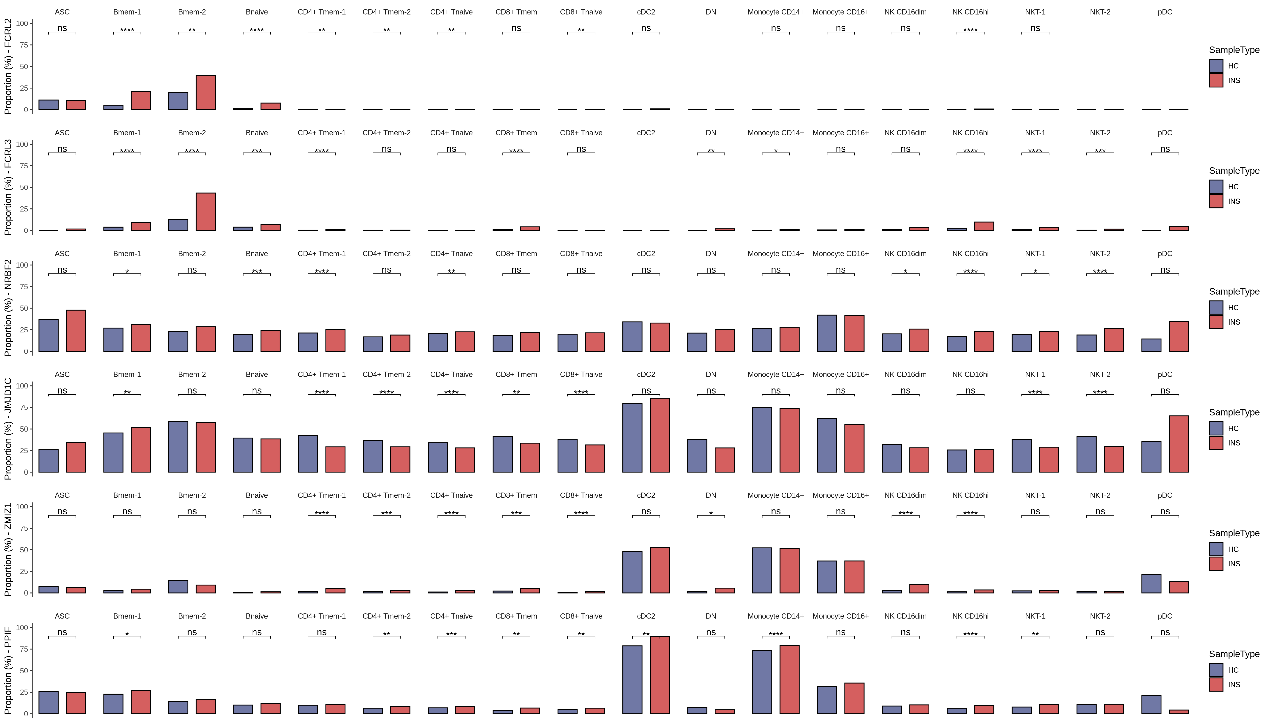


C)
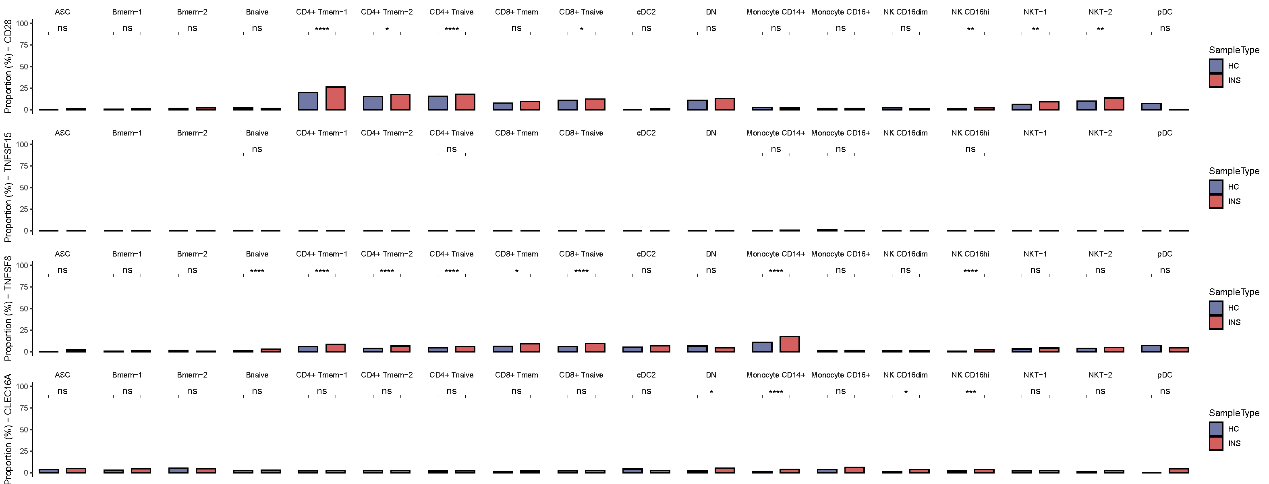


Supplementary Figure 4. Expression levels of candidate genes within respective immune subclusters in healthy children (HC, gray) and idiopathic nephrotic syndrome patients (INS, red), grouped by annotation type. A, genes newly identified in this study with no prior GWAS associations in pSSNS or IgAN; B, genes previously reported in IgAN GWAS but newly associated with pSSNS; C, known genes reported in both pSSNS and IgAN GWAS. Expression levels are calculated based on normalized transcript counts from single-nucleus RNA-seq data. P-values were determined using Wilcoxon rank-sum tests: ****, p < 1e-4; ***, p<0.001; **, p<0.01; *, p<0.05; ns, not significant.

A)


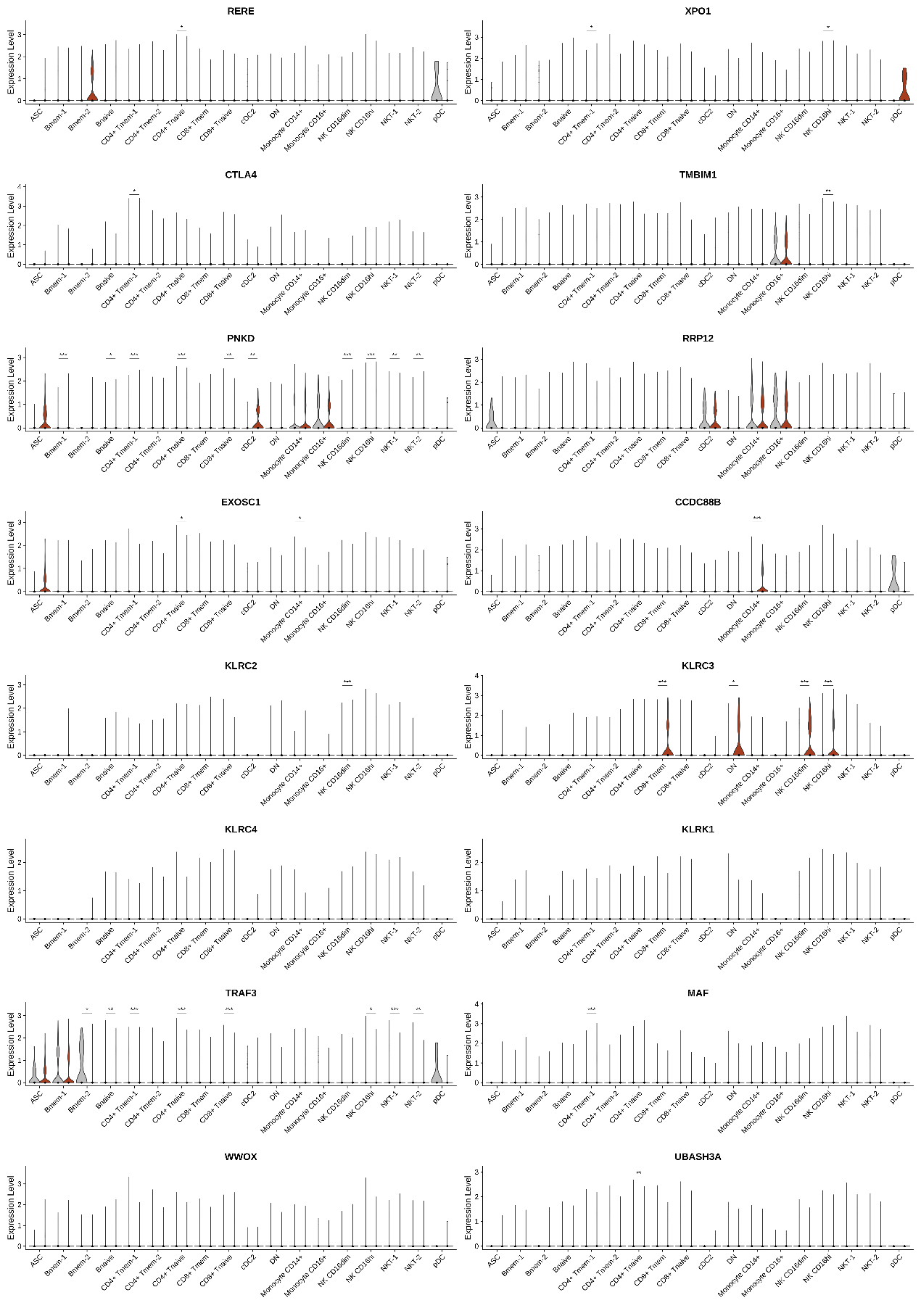


B)


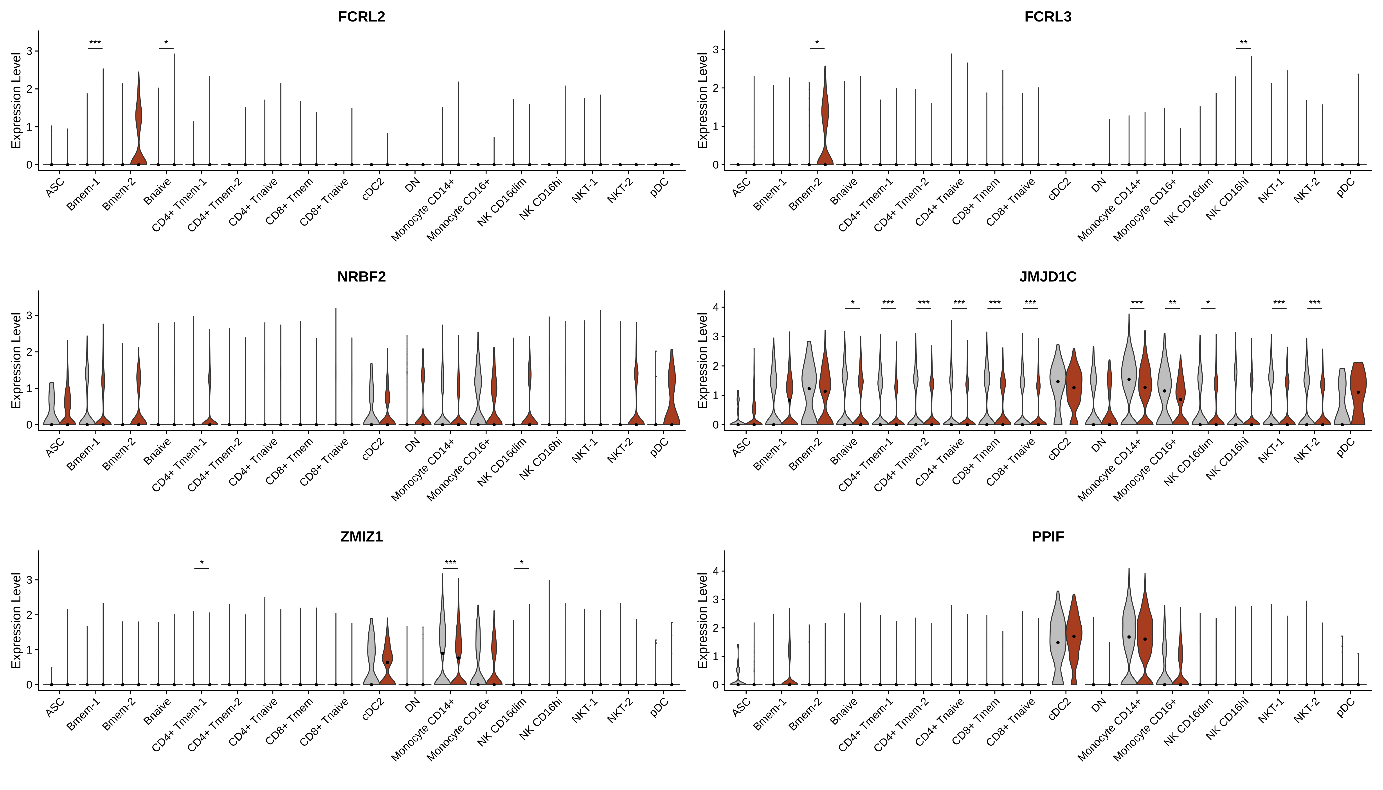


C)


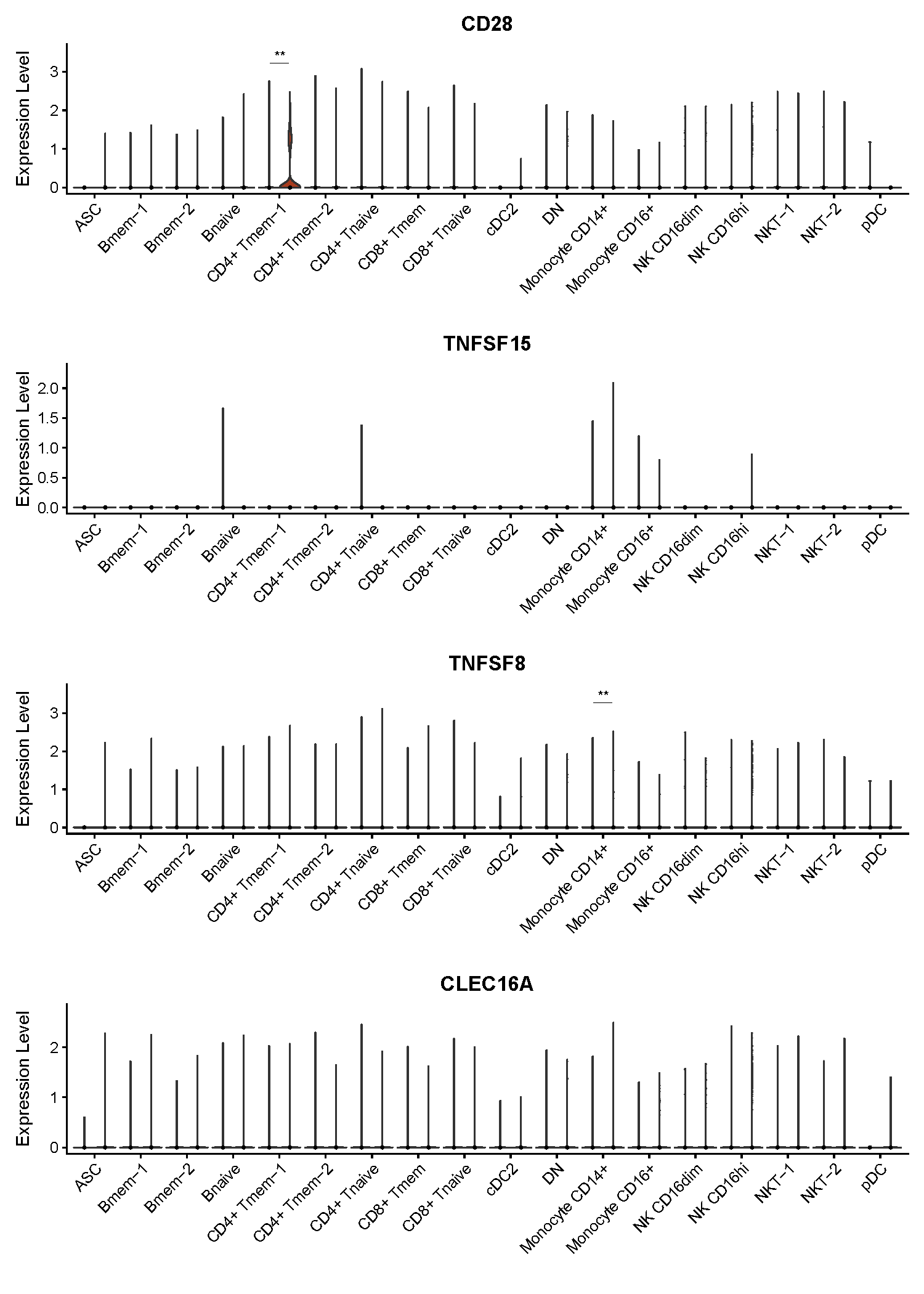

Supplement: Supplementary file 1 [file DataSheet1.docx]
