# Supplementary material for: Identification of IL7R as a key genetic risk locus in childhood steroid-sensitive nephrotic syndrome and IgA nephropathy
Source: Front Immunol. 2026 May 29;17:1806680. doi: 10.3389/fimmu.2026.1806680 (PMC13260247; doi:10.3389/fimmu.2026.1806680)
Supplement: Supplementary file 2 [file DataSheet2.docx]

Table S1. Single-Tissue eQTLs for novel loci identified in the meta-analysis and conjunctional FDR analysis

| **SNP** | **Cytogenetic band** | **CHR** | **BP** | **Gene** | ***P*-value** | **NES** | **Tissue** |
| --- | --- | --- | --- | --- | --- | --- | --- |
| rs1763839 | 1p36.23 | 1 | 8473336 | ENO1 | 0.000038 | -0.097 | Heart - Atrial Appendage |
|  |  |  |  |  | 0.00016 | -0.052 | Nerve - Tibial |
|  |  |  |  | RERE | 4.00E-43 | 0.32 | Adipose - Subcutaneous |
|  |  |  |  |  | 5.90E-22 | 0.13 | Whole Blood |
|  |  |  |  |  | 9.50E-22 | 0.25 | Adipose - Visceral (Omentum) |
|  |  |  |  |  | 1.40E-21 | 0.38 | Brain - Caudate (basal ganglia) |
|  |  |  |  |  | 1.30E-19 | 0.2 | Nerve - Tibial |
|  |  |  |  |  | 9.00E-17 | 0.2 | Breast - Mammary Tissue |
|  |  |  |  |  | 3.10E-15 | 0.18 | Lung |
|  |  |  |  |  | 5.20E-15 | 0.32 | Brain - Cortex |
|  |  |  |  |  | 2.80E-14 | 0.31 | Brain - Anterior qcingulate cortex (BA24) |
|  |  |  |  |  | 1.80E-13 | 0.37 | Brain - Putamen (basal ganglia) |
|  |  |  |  |  | 1.30E-12 | 0.18 | Cells - Cultured fibroblasts |
|  |  |  |  |  | 1.00E-11 | 0.26 | Brain - Hippocampus |
|  |  |  |  |  | 7.70E-11 | 0.23 | Brain - Frontal Cortex (BA9) |
|  |  |  |  |  | 2.40E-10 | 0.16 | Esophagus - Mucosa |
|  |  |  |  |  | 3.10E-10 | 0.27 | Brain - Nucleus accumbens (basal ganglia) |
|  |  |  |  |  | 1.60E-08 | 0.36 | Brain - Amygdala |
|  |  |  |  |  | 1.70E-08 | 0.092 | Skin - Not Sun Exposed (Suprapubic) |
|  |  |  |  |  | 1.50E-07 | 0.087 | Skin - Sun Exposed (Lower leg) |
|  |  |  |  |  | 3.10E-07 | 0.2 | Pancreas |
|  |  |  |  |  | 0.0000064 | 0.081 | Heart - Left Ventricle |
|  |  |  |  |  | 0.000011 | 0.1 | Thyroid |
|  |  |  |  |  | 0.000011 | 0.22 | Vagina |
|  |  |  |  | RERE-AS1 | 5.00E-25 | 0.4 | Adipose - Subcutaneous |
|  |  |  |  |  | 1.70E-14 | 0.27 | Thyroid |
|  |  |  |  |  | 2.70E-10 | 0.25 | Lung |
|  |  |  |  |  | 3.00E-10 | 0.27 | Breast - Mammary Tissue |
|  |  |  |  |  | 4.90E-09 | 0.25 | Nerve - Tibial |
|  |  |  |  |  | 6.30E-09 | 0.26 | Esophagus - Mucosa |
|  |  |  |  |  | 1.70E-08 | 0.22 | Skin - Sun Exposed (Lower leg) |
|  |  |  |  |  | 3.80E-08 | 0.17 | Muscle - Skeletal |
|  |  |  |  |  | 4.10E-08 | 0.23 | Adipose - Visceral (Omentum) |
|  |  |  |  |  | 5.20E-08 | 0.24 | Skin - Not Sun Exposed (Suprapubic) |
|  |  |  |  |  | 0.0000013 | 0.18 | Artery - Tibial |
|  |  |  |  |  | 0.000002 | 0.29 | Brain - Cerebellar Hemisphere |
|  |  |  |  |  | 0.0000025 | 0.41 | Minor Salivary Gland |
|  |  |  |  |  | 0.0000068 | 0.38 | Brain - Putamen (basal ganglia) |
|  |  |  |  |  | 0.0000082 | 0.24 | Heart - Atrial Appendage |
|  |  |  |  |  | 0.000013 | 0.18 | Esophagus - Muscularis |
|  |  |  |  |  | 0.000015 | 0.34 | Small Intestine - Terminal Ileum |
|  |  |  |  |  | 0.000015 | 0.073 | Whole Blood |
|  |  |  |  |  | 0.000046 | 0.17 | Colon - Transverse |
|  |  |  |  |  | 0.000072 | 0.21 | Esophagus - Gastroesophageal Junction |
| rs75873622 | 1p36.13 | 1 | 18766556 | KLHDC7A | 0.0000012 | -0.33 | Esophagus - Mucosa |
|  |  |  |  |  | 0.000022 | -0.14 | Thyroid |
|  |  |  |  |  | 0.000061 | -0.27 | Adipose - Subcutaneous |
| rs7524764 | 1q23.1 | 1 | 157685739 | FCRL1 | 1.50E-15 | -0.31 | Whole Blood |
|  |  |  |  | FCRL2 | 3.40E-07 | 0.39 | Minor Salivary Gland |
|  |  |  |  |  | 0.00011 | 0.11 | Colon - Transverse |
|  |  |  |  | FCRL3 | 4.40E-07 | 0.27 | Lung |
|  |  |  |  |  | 6.80E-07 | 0.15 | Whole Blood |
|  |  |  |  |  | 0.0000029 | 0.22 | Esophagus - Mucosa |
|  |  |  |  |  | 0.000041 | 0.23 | Adipose - Subcutaneous |
| rs3818813 | 1q23.1 | 1 | 157718325 | FCRL1 | 8.10E-14 | -0.27 | Whole Blood |
|  |  |  |  | FCRL2 | 4.90E-10 | 0.43 | Minor Salivary Gland |
|  |  |  |  |  | 6.00E-08 | 0.15 | Colon - Transverse |
|  |  |  |  | FCRL3 | 9.20E-08 | 0.15 | Whole Blood |
|  |  |  |  |  | 0.0000017 | 0.25 | Lung |
|  |  |  |  |  | 0.0000056 | 0.2 | Esophagus - Mucosa |
|  |  |  |  |  | 0.000013 | 0.24 | Adipose - Subcutaneous |
| rs3771258 | 2p15 | 2 | 61764140 | AHSA2P | 6.00E-50 | -0.36 | Whole Blood |
|  |  |  |  |  | 9.40E-20 | -0.32 | Nerve - Tibial |
|  |  |  |  |  | 2.90E-19 | -0.18 | Muscle - Skeletal |
|  |  |  |  |  | 3.70E-19 | -0.28 | Adipose - Subcutaneous |
|  |  |  |  |  | 7.20E-16 | -0.15 | Skin - Not Sun Exposed (Suprapubic) |
|  |  |  |  |  | 6.70E-15 | -0.14 | Skin - Sun Exposed (Lower leg) |
|  |  |  |  |  | 1.20E-12 | -0.15 | Esophagus - Mucosa |
|  |  |  |  |  | 2.70E-12 | -0.2 | Adipose - Visceral (Omentum) |
|  |  |  |  |  | 3.60E-12 | -0.22 | Breast - Mammary Tissue |
|  |  |  |  |  | 5.50E-12 | -0.4 | Brain - Hippocampus |
|  |  |  |  |  | 1.30E-11 | -0.2 | Esophagus - Muscularis |
|  |  |  |  |  | 1.50E-11 | -0.24 | Liver |
|  |  |  |  |  | 1.70E-11 | -0.42 | Brain - Putamen (basal ganglia) |
|  |  |  |  |  | 4.60E-11 | -0.32 | Brain - Caudate (basal ganglia) |
|  |  |  |  |  | 6.50E-11 | -0.13 | Cells - Cultured fibroblasts |
|  |  |  |  |  | 9.50E-11 | -0.17 | Thyroid |
|  |  |  |  |  | 9.60E-11 | -0.32 | Testis |
|  |  |  |  |  | 1.50E-10 | -0.35 | Brain - Cortex |
|  |  |  |  |  | 1.60E-10 | -0.38 | Pituitary |
|  |  |  |  |  | 2.30E-10 | -0.46 | Brain - Cerebellum |
|  |  |  |  |  | 5.80E-10 | -0.34 | Brain - Nucleus accumbens (basal ganglia) |
|  |  |  |  |  | 8.40E-10 | -0.22 | Colon - Transverse |
|  |  |  |  |  | 1.80E-09 | -0.15 | Lung |
|  |  |  |  |  | 2.40E-09 | -0.29 | Brain - Frontal Cortex (BA9) |
|  |  |  |  |  | 2.70E-09 | -0.23 | Colon - Sigmoid |
|  |  |  |  |  | 3.80E-09 | -0.19 | Heart - Atrial Appendage |
|  |  |  |  |  | 1.40E-08 | -0.32 | Brain - Anterior qcingulate cortex (BA24) |
|  |  |  |  |  | 1.40E-08 | -0.42 | Brain - Spinal cord (cervical c-1) |
|  |  |  |  |  | 6.50E-08 | -0.31 | Brain - Cerebellar Hemisphere |
|  |  |  |  |  | 2.90E-07 | -0.26 | Prostate |
|  |  |  |  |  | 3.70E-07 | -0.31 | Brain - Hypothalamus |
|  |  |  |  |  | 8.90E-07 | -0.31 | Brain - Substantia nigra |
|  |  |  |  |  | 0.0000019 | -0.13 | Artery - Tibial |
|  |  |  |  |  | 0.0000037 | -0.17 | Pancreas |
|  |  |  |  |  | 0.0000048 | -0.35 | Brain - Amygdala |
|  |  |  |  |  | 0.0000053 | -0.29 | Ovary |
|  |  |  |  |  | 0.000018 | -0.11 | Heart - Left Ventricle |
|  |  |  |  |  | 0.000021 | -0.22 | Spleen |
|  |  |  |  |  | 0.000028 | -0.17 | Esophagus - Gastroesophageal Junction |
|  |  |  |  |  | 0.00006 | -0.14 | Stomach |
|  |  |  |  |  | 0.00015 | -0.12 | Artery - Aorta |
|  |  |  |  | C2orf74 | 4.60E-58 | 0.59 | Muscle - Skeletal |
|  |  |  |  |  | 1.00E-49 | 0.65 | Adipose - Subcutaneous |
|  |  |  |  |  | 2.70E-48 | 0.71 | Cells - Cultured fibroblasts |
|  |  |  |  |  | 1.70E-46 | 0.61 | Thyroid |
|  |  |  |  |  | 3.00E-44 | 0.68 | Esophagus - Mucosa |
|  |  |  |  |  | 6.60E-44 | 0.62 | Artery - Tibial |
|  |  |  |  |  | 2.40E-43 | 0.57 | Nerve - Tibial |
|  |  |  |  |  | 4.60E-42 | 0.55 | Skin - Sun Exposed (Lower leg) |
|  |  |  |  |  | 1.20E-41 | 0.66 | Esophagus - Muscularis |
|  |  |  |  |  | 1.40E-40 | 0.63 | Adipose - Visceral (Omentum) |
|  |  |  |  |  | 1.90E-36 | 0.51 | Lung |
|  |  |  |  |  | 1.30E-34 | 0.52 | Skin - Not Sun Exposed (Suprapubic) |
|  |  |  |  |  | 6.10E-34 | 0.58 | Breast - Mammary Tissue |
|  |  |  |  |  | 5.80E-29 | 0.35 | Colon - Transverse |
|  |  |  |  |  | 5.60E-27 | 0.63 | Artery - Aorta |
|  |  |  |  |  | 1.00E-26 | 0.55 | Colon - Sigmoid |
|  |  |  |  |  | 3.00E-26 | 0.61 | Stomach |
|  |  |  |  |  | 4.70E-25 | 0.51 | Heart - Atrial Appendage |
|  |  |  |  |  | 1.40E-23 | 0.45 | Heart - Left Ventricle |
|  |  |  |  |  | 1.50E-22 | 0.72 | Brain - Cortex |
|  |  |  |  |  | 1.50E-22 | 0.62 | Esophagus - Gastroesophageal Junction |
|  |  |  |  |  | 3.80E-22 | 0.72 | Cells - EBV-transformed lymphocytes |
|  |  |  |  |  | 6.60E-20 | 0.58 | Pituitary |
|  |  |  |  |  | 4.40E-19 | 0.71 | Spleen |
|  |  |  |  |  | 6.10E-19 | 0.55 | Liver |
|  |  |  |  |  | 2.00E-18 | 0.57 | Ovary |
|  |  |  |  |  | 1.50E-17 | 0.68 | Brain - Cerebellum |
|  |  |  |  |  | 1.70E-17 | 0.53 | Brain - Frontal Cortex (BA9) |
|  |  |  |  |  | 1.90E-17 | 0.55 | Adrenal Gland |
|  |  |  |  |  | 2.00E-17 | 0.58 | Pancreas |
|  |  |  |  |  | 5.60E-17 | 0.58 | Brain - Cerebellar Hemisphere |
|  |  |  |  |  | 6.80E-17 | 0.31 | Whole Blood |
|  |  |  |  |  | 7.00E-17 | 0.66 | Artery - Coronary |
|  |  |  |  |  | 8.00E-17 | 0.54 | Prostate |
|  |  |  |  |  | 3.10E-15 | 0.3 | Brain - Nucleus accumbens (basal ganglia) |
|  |  |  |  |  | 2.50E-14 | 0.33 | Brain - Putamen (basal ganglia) |
|  |  |  |  |  | 1.10E-13 | 0.44 | Brain - Anterior qcingulate cortex (BA24) |
|  |  |  |  |  | 7.30E-13 | 0.29 | Brain - Caudate (basal ganglia) |
|  |  |  |  |  | 2.30E-12 | 0.63 | Minor Salivary Gland |
|  |  |  |  |  | 2.50E-12 | 0.53 | Vagina |
|  |  |  |  |  | 6.30E-11 | 0.36 | Brain - Hypothalamus |
|  |  |  |  |  | 7.30E-11 | 0.14 | Testis |
|  |  |  |  |  | 5.40E-10 | 0.33 | Brain - Hippocampus |
|  |  |  |  |  | 2.20E-09 | 0.35 | Brain - Spinal cord (cervical c-1) |
|  |  |  |  |  | 3.30E-09 | 0.59 | Uterus |
|  |  |  |  |  | 6.00E-09 | 0.65 | Bladder |
|  |  |  |  |  | 3.20E-08 | 0.26 | Small Intestine - Terminal Ileum |
|  |  |  |  |  | 1.00E-07 | 0.33 | Brain - Amygdala |
|  |  |  |  |  | 1.70E-07 | 0.31 | Brain - Substantia nigra |
|  |  |  |  | ENSG00000212978 | 5.90E-66 | -0.87 | Testis |
|  |  |  |  |  | 7.80E-25 | -0.3 | Whole Blood |
|  |  |  |  |  | 1.00E-14 | -0.42 | Spleen |
|  |  |  |  |  | 1.20E-14 | -0.22 | Adipose - Visceral (Omentum) |
|  |  |  |  |  | 5.40E-14 | -0.35 | Heart - Left Ventricle |
|  |  |  |  |  | 7.20E-14 | -0.24 | Muscle - Skeletal |
|  |  |  |  |  | 6.80E-13 | -0.27 | Heart - Atrial Appendage |
|  |  |  |  |  | 5.00E-11 | -0.29 | Pituitary |
|  |  |  |  |  | 7.80E-07 | -0.16 | Adipose - Subcutaneous |
|  |  |  |  |  | 3.90E-06 | -0.15 | Breast - Mammary Tissue |
|  |  |  |  |  | 4.10E-06 | -0.1 | Lung |
|  |  |  |  |  | 5.40E-06 | -0.16 | Nerve - Tibial |
|  |  |  |  |  | 5.60E-06 | -0.18 | Esophagus - Muscularis |
|  |  |  |  |  | 4.00E-05 | -0.21 | Pancreas |
|  |  |  |  |  | 6.00E-05 | -0.11 | Thyroid |
|  |  |  |  | ENSG00000271889 | 1.70E-21 | 0.45 | Cells - Cultured fibroblasts |
|  |  |  |  |  | 8.10E-15 | 0.21 | Testis |
|  |  |  |  |  | 6.50E-14 | 0.35 | Thyroid |
|  |  |  |  |  | 4.90E-06 | 0.39 | Brain - Cerebellum |
|  |  |  |  |  | 5.00E-06 | 0.23 | Esophagus - Mucosa |
|  |  |  |  |  | 7.10E-06 | 0.25 | Esophagus - Muscularis |
|  |  |  |  |  | 2.30E-05 | 0.28 | Adrenal Gland |
|  |  |  |  |  | 2.50E-05 | 0.2 | Lung |
|  |  |  |  |  | 3.20E-05 | 0.32 | Ovary |
|  |  |  |  |  | 3.60E-05 | 0.23 | Breast - Mammary Tissue |
|  |  |  |  |  | 3.80E-05 | 0.33 | Pancreas |
|  |  |  |  |  | 4.90E-05 | 0.25 | Colon - Transverse |
|  |  |  |  |  | 5.30E-05 | 0.24 | Stomach |
|  |  |  |  |  | 6.40E-05 | 0.24 | Pituitary |
|  |  |  |  |  | 3.90E-04 | 0.17 | Nerve - Tibial |
|  |  |  |  | ENSG00000273302 | 6.20E-19 | 0.36 | Nerve - Tibial |
|  |  |  |  |  | 6.20E-15 | 0.36 | Esophagus - Muscularis |
|  |  |  |  |  | 1.60E-11 | 0.3 | Adipose - Subcutaneous |
|  |  |  |  |  | 2.60E-11 | 0.45 | Brain - Hippocampus |
|  |  |  |  |  | 1.40E-10 | 0.51 | Prostate |
|  |  |  |  |  | 5.00E-10 | 0.24 | Thyroid |
|  |  |  |  |  | 2.00E-09 | 0.35 | Colon - Transverse |
|  |  |  |  |  | 8.00E-09 | 0.3 | Colon - Sigmoid |
|  |  |  |  |  | 1.60E-08 | 0.16 | Whole Blood |
|  |  |  |  |  | 1.30E-07 | 0.38 | Ovary |
|  |  |  |  |  | 3.30E-07 | 0.31 | Testis |
|  |  |  |  |  | 4.20E-07 | 0.33 | Brain - Nucleus accumbens (basal ganglia) |
|  |  |  |  |  | 5.50E-07 | 0.24 | Lung |
|  |  |  |  |  | 1.10E-06 | 0.26 | Artery - Aorta |
|  |  |  |  |  | 1.90E-06 | 0.24 | Esophagus - Mucosa |
|  |  |  |  |  | 3.10E-06 | 0.2 | Artery - Tibial |
|  |  |  |  |  | 3.50E-06 | 0.21 | Muscle - Skeletal |
|  |  |  |  |  | 5.10E-06 | 0.38 | Brain - Spinal cord (cervical c-1) |
|  |  |  |  |  | 6.20E-06 | 0.27 | Stomach |
|  |  |  |  |  | 6.80E-06 | 0.23 | Breast - Mammary Tissue |
|  |  |  |  |  | 9.50E-06 | 0.25 | Esophagus - Gastroesophageal Junction |
|  |  |  |  |  | 1.50E-05 | 0.35 | Brain - Cortex |
|  |  |  |  |  | 2.30E-05 | 0.32 | Brain - Cerebellum |
|  |  |  |  |  | 2.60E-05 | 0.35 | Brain - Anterior qcingulate cortex (BA24) |
|  |  |  |  |  | 2.70E-05 | 0.29 | Adrenal Gland |
|  |  |  |  |  | 3.50E-05 | 0.29 | Brain - Caudate (basal ganglia) |
|  |  |  |  |  | 1.20E-04 | 0.28 | Spleen |
|  |  |  |  |  | 1.40E-04 | 0.24 | Pituitary |
|  |  |  |  |  | 1.60E-04 | 0.25 | Cells - EBV-transformed lymphocytes |
|  |  |  |  | ENSG00000289410 | 8.30E-06 | 0.15 | Muscle - Skeletal |
|  |  |  |  | NONOP2 | 3.10E-05 | -0.14 | Nerve - Tibial |
|  |  |  |  | PUS10 | 1.90E-07 | -0.15 | Nerve - Tibial |
|  |  |  |  |  | 2.70E-05 | -0.16 | Colon - Sigmoid |
|  |  |  |  |  | 1.10E-04 | -0.13 | Testis |
|  |  |  |  |  | 1.50E-04 | -0.11 | Esophagus - Mucosa |
|  |  |  |  |  | 2.10E-04 | -0.12 | Artery - Aorta |
|  |  |  |  | SANBR | 8.60E-05 | -0.17 | Brain - Cerebellum |
|  |  |  |  | USP34 | 1.40E-06 | -0.095 | Cells - Cultured fibroblasts |
|  |  |  |  |  | 2.00E-05 | -0.075 | Lung |
|  |  |  |  |  | 5.30E-05 | -0.057 | Skin - Sun Exposed (Lower leg) |
|  |  |  |  | USP34-DT | 3.80E-22 | 0.23 | Thyroid |
|  |  |  |  |  | 5.10E-16 | 0.19 | Whole Blood |
|  |  |  |  |  | 9.60E-14 | -0.22 | Muscle - Skeletal |
|  |  |  |  |  | 1.40E-13 | 0.17 | Nerve - Tibial |
|  |  |  |  |  | 1.40E-12 | 0.22 | Skin - Sun Exposed (Lower leg) |
|  |  |  |  |  | 3.40E-12 | 0.3 | Testis |
|  |  |  |  |  | 4.30E-11 | 0.19 | Skin - Not Sun Exposed (Suprapubic) |
|  |  |  |  |  | 5.20E-09 | 0.21 | Pituitary |
|  |  |  |  |  | 3.00E-08 | 0.26 | Spleen |
|  |  |  |  |  | 6.10E-06 | 0.12 | Adipose - Subcutaneous |
|  |  |  |  |  | 2.00E-05 | 0.24 | Brain - Hypothalamus |
|  |  |  |  |  | 6.70E-05 | 0.17 | Brain - Cerebellar Hemisphere |
|  |  |  |  | XPO1 | 4.10E-08 | 0.2 | Brain - Cerebellum |
|  |  |  |  |  | 5.60E-07 | 0.19 | Brain - Cerebellar Hemisphere |
|  |  |  |  |  | 6.00E-05 | -0.067 | Testis |
| rs231805 | 2q33.2 | 2 | 204708749 | CD28 | 1.10E-04 | -0.18 | Breast - Mammary Tissue |
| rs13429408 | 2q35 | 2 | 219142860 | AAMP | 1.30E-08 | 0.087 | Nerve - Tibial |
|  |  |  |  |  | 2.60E-08 | 0.087 | Adipose - Subcutaneous |
|  |  |  |  |  | 3.90E-08 | 0.098 | Skin - Not Sun Exposed (Suprapubic) |
|  |  |  |  |  | 5.30E-08 | 0.14 | Colon - Sigmoid |
|  |  |  |  |  | 7.20E-08 | 0.15 | Heart - Atrial Appendage |
|  |  |  |  |  | 1.70E-06 | 0.077 | Adipose - Visceral (Omentum) |
|  |  |  |  |  | 6.20E-06 | 0.063 | Skin - Sun Exposed (Lower leg) |
|  |  |  |  |  | 2.50E-05 | 0.1 | Stomach |
|  |  |  |  |  | 4.40E-05 | 0.083 | Breast - Mammary Tissue |
|  |  |  |  |  | 9.50E-05 | 0.072 | Artery - Tibial |
|  |  |  |  | ARPC2 | 7.20E-41 | 0.28 | Muscle - Skeletal |
|  |  |  |  |  | 1.80E-30 | 0.25 | Nerve - Tibial |
|  |  |  |  |  | 2.30E-26 | 0.19 | Lung |
|  |  |  |  |  | 5.90E-24 | 0.21 | Adipose - Subcutaneous |
|  |  |  |  |  | 1.80E-17 | 0.21 | Spleen |
|  |  |  |  |  | 1.40E-12 | 0.13 | Thyroid |
|  |  |  |  |  | 6.30E-12 | 0.22 | Pancreas |
|  |  |  |  |  | 3.20E-10 | 0.12 | Skin - Not Sun Exposed (Suprapubic) |
|  |  |  |  |  | 5.70E-09 | 0.087 | Skin - Sun Exposed (Lower leg) |
|  |  |  |  |  | 6.20E-09 | 0.093 | Breast - Mammary Tissue |
|  |  |  |  |  | 1.50E-08 | 0.069 | Whole Blood |
|  |  |  |  |  | 3.30E-08 | 0.13 | Heart - Atrial Appendage |
|  |  |  |  |  | 4.30E-08 | 0.13 | Heart - Left Ventricle |
|  |  |  |  |  | 8.00E-07 | 0.11 | Colon - Transverse |
|  |  |  |  |  | 1.80E-06 | 0.12 | Stomach |
|  |  |  |  |  | 1.80E-06 | 0.15 | Prostate |
|  |  |  |  |  | 3.20E-06 | 0.19 | Minor Salivary Gland |
|  |  |  |  |  | 5.30E-06 | 0.21 | Brain - Cerebellum |
|  |  |  |  |  | 6.10E-06 | 0.16 | Pituitary |
|  |  |  |  |  | 8.30E-06 | 0.23 | Small Intestine - Terminal Ileum |
|  |  |  |  |  | 1.70E-05 | 0.13 | Colon - Sigmoid |
|  |  |  |  |  | 3.20E-05 | 0.16 | Brain - Cerebellar Hemisphere |
|  |  |  |  | CXCR1 | 3.20E-07 | 0.054 | Whole Blood |
|  |  |  |  | CXCR2 | 1.90E-06 | 0.05 | Whole Blood |
|  |  |  |  | ENSG00000261338 | 6.10E-103 | 0.72 | Skin - Sun Exposed (Lower leg) |
|  |  |  |  |  | 1.20E-99 | 0.83 | Thyroid |
|  |  |  |  |  | 6.00E-97 | 0.74 | Muscle - Skeletal |
|  |  |  |  |  | 6.70E-84 | 0.7 | Adipose - Subcutaneous |
|  |  |  |  |  | 6.60E-83 | 0.78 | Esophagus - Muscularis |
|  |  |  |  |  | 2.00E-82 | 0.77 | Artery - Tibial |
|  |  |  |  |  | 2.40E-80 | 0.78 | Esophagus - Mucosa |
|  |  |  |  |  | 3.50E-80 | 0.64 | Lung |
|  |  |  |  |  | 4.10E-80 | 0.76 | Nerve - Tibial |
|  |  |  |  |  | 4.30E-78 | 0.69 | Skin - Not Sun Exposed (Suprapubic) |
|  |  |  |  |  | 2.50E-70 | 0.79 | Brain - Nucleus accumbens (basal ganglia) |
|  |  |  |  |  | 4.40E-68 | 0.73 | Colon - Sigmoid |
|  |  |  |  |  | 6.70E-68 | 0.93 | Brain - Cerebellar Hemisphere |
|  |  |  |  |  | 1.10E-66 | 0.94 | Pituitary |
|  |  |  |  |  | 3.80E-66 | 0.74 | Brain - Caudate (basal ganglia) |
|  |  |  |  |  | 1.50E-65 | 0.8 | Artery - Aorta |
|  |  |  |  |  | 3.00E-65 | 0.71 | Colon - Transverse |
|  |  |  |  |  | 3.40E-65 | 0.91 | Brain - Frontal Cortex (BA9) |
|  |  |  |  |  | 5.80E-63 | 0.64 | Cells - Cultured fibroblasts |
|  |  |  |  |  | 1.70E-62 | 0.97 | Brain - Cortex |
|  |  |  |  |  | 8.70E-61 | 0.68 | Adipose - Visceral (Omentum) |
|  |  |  |  |  | 1.00E-60 | 1 | Brain - Cerebellum |
|  |  |  |  |  | 2.80E-59 | 0.33 | Whole Blood |
|  |  |  |  |  | 9.30E-55 | 0.69 | Brain - Putamen (basal ganglia) |
|  |  |  |  |  | 9.90E-55 | 0.87 | Brain - Hypothalamus |
|  |  |  |  |  | 7.70E-54 | 0.84 | Stomach |
|  |  |  |  |  | 7.50E-53 | 0.75 | Esophagus - Gastroesophageal Junction |
|  |  |  |  |  | 8.20E-51 | 0.61 | Breast - Mammary Tissue |
|  |  |  |  |  | 1.60E-50 | 0.8 | Heart - Atrial Appendage |
|  |  |  |  |  | 3.90E-49 | 0.83 | Brain - Anterior qcingulate cortex (BA24) |
|  |  |  |  |  | 1.10E-44 | 0.8 | Heart - Left Ventricle |
|  |  |  |  |  | 5.00E-41 | 0.83 | Spleen |
|  |  |  |  |  | 1.40E-40 | 0.84 | Adrenal Gland |
|  |  |  |  |  | 2.70E-40 | 0.59 | Brain - Hippocampus |
|  |  |  |  |  | 5.90E-37 | 0.77 | Prostate |
|  |  |  |  |  | 1.40E-36 | 0.59 | Testis |
|  |  |  |  |  | 9.30E-31 | 0.79 | Brain - Amygdala |
|  |  |  |  |  | 9.90E-31 | 0.73 | Brain - Substantia nigra |
|  |  |  |  |  | 5.80E-30 | 0.84 | Brain - Spinal cord (cervical c-1) |
|  |  |  |  |  | 5.10E-29 | 0.76 | Artery - Coronary |
|  |  |  |  |  | 8.60E-25 | 0.63 | Small Intestine - Terminal Ileum |
|  |  |  |  |  | 9.20E-23 | 0.69 | Pancreas |
|  |  |  |  |  | 5.30E-20 | 0.45 | Cells - EBV-transformed lymphocytes |
|  |  |  |  |  | 4.10E-17 | 0.66 | Uterus |
|  |  |  |  |  | 2.70E-16 | 0.69 | Minor Salivary Gland |
|  |  |  |  |  | 6.20E-14 | 0.58 | Vagina |
|  |  |  |  |  | 8.80E-14 | 0.61 | Liver |
|  |  |  |  |  | 9.30E-13 | 0.55 | Ovary |
|  |  |  |  |  | 8.40E-12 | 0.73 | Bladder |
|  |  |  |  |  | 4.20E-09 | 0.75 | Kidney - Cortex |
|  |  |  |  | GPBAR1 | 8.00E-84 | -0.74 | Testis |
|  |  |  |  |  | 1.50E-08 | 0.24 | Heart - Atrial Appendage |
|  |  |  |  |  | 9.60E-08 | 0.32 | Pituitary |
|  |  |  |  |  | 2.10E-07 | 0.17 | Skin - Sun Exposed (Lower leg) |
|  |  |  |  |  | 3.10E-07 | 0.2 | Artery - Tibial |
|  |  |  |  |  | 0.000002 | 0.17 | Thyroid |
|  |  |  |  | PNKD | 3.30E-66 | 0.52 | Skin - Sun Exposed (Lower leg) |
|  |  |  |  |  | 4.00E-45 | 0.51 | Skin - Not Sun Exposed (Suprapubic) |
|  |  |  |  |  | 1.20E-29 | 0.26 | Thyroid |
|  |  |  |  |  | 2.60E-26 | 0.36 | Testis |
|  |  |  |  |  | 3.50E-20 | 0.22 | Whole Blood |
|  |  |  |  |  | 3.90E-20 | 0.43 | Adrenal Gland |
|  |  |  |  |  | 6.10E-19 | 0.38 | Pituitary |
|  |  |  |  |  | 3.00E-12 | 0.19 | Heart - Atrial Appendage |
|  |  |  |  |  | 2.10E-10 | 0.3 | Spleen |
|  |  |  |  |  | 3.00E-09 | 0.13 | Adipose - Visceral (Omentum) |
|  |  |  |  |  | 8.10E-09 | -0.18 | Brain - Nucleus accumbens (basal ganglia) |
|  |  |  |  |  | 1.30E-08 | 0.52 | Kidney - Cortex |
|  |  |  |  |  | 2.90E-08 | 0.13 | Adipose - Subcutaneous |
|  |  |  |  |  | 3.60E-08 | 0.1 | Lung |
|  |  |  |  |  | 6.70E-08 | 0.23 | Liver |
|  |  |  |  |  | 2.50E-07 | -0.15 | Brain - Caudate (basal ganglia) |
|  |  |  |  |  | 2.50E-07 | -0.17 | Brain - Putamen (basal ganglia) |
|  |  |  |  |  | 0.0000019 | -0.12 | Brain - Frontal Cortex (BA9) |
|  |  |  |  |  | 0.0000065 | -0.14 | Brain - Cortex |
|  |  |  |  |  | 0.000011 | 0.1 | Cells - Cultured fibroblasts |
|  |  |  |  |  | 0.000053 | 0.1 | Breast - Mammary Tissue |
|  |  |  |  | TMBIM1 | 2.10E-56 | 0.31 | Cells - Cultured fibroblasts |
|  |  |  |  |  | 2.50E-42 | 0.39 | Heart - Atrial Appendage |
|  |  |  |  |  | 2.10E-38 | 0.24 | Artery - Tibial |
|  |  |  |  |  | 3.70E-33 | 0.34 | Artery - Aorta |
|  |  |  |  |  | 1.40E-29 | 0.25 | Muscle - Skeletal |
|  |  |  |  |  | 1.10E-28 | 0.29 | Heart - Left Ventricle |
|  |  |  |  |  | 1.10E-23 | 0.34 | Pituitary |
|  |  |  |  |  | 3.10E-23 | 0.2 | Testis |
|  |  |  |  |  | 2.80E-21 | 0.15 | Nerve - Tibial |
|  |  |  |  |  | 1.50E-20 | 0.14 | Adipose - Subcutaneous |
|  |  |  |  |  | 3.80E-16 | 0.29 | Cells - EBV-transformed lymphocytes |
|  |  |  |  |  | 6.20E-16 | 0.17 | Esophagus - Muscularis |
|  |  |  |  |  | 1.10E-14 | 0.1 | Esophagus - Mucosa |
|  |  |  |  |  | 8.70E-14 | 0.12 | Adipose - Visceral (Omentum) |
|  |  |  |  |  | 9.70E-14 | 0.22 | Colon - Sigmoid |
|  |  |  |  |  | 1.90E-13 | 0.15 | Colon - Transverse |
|  |  |  |  |  | 5.10E-10 | 0.095 | Breast - Mammary Tissue |
|  |  |  |  |  | 7.10E-10 | 0.14 | Lung |
|  |  |  |  |  | 9.10E-09 | 0.15 | Esophagus - Gastroesophageal Junction |
|  |  |  |  |  | 1.60E-08 | 0.23 | Artery - Coronary |
|  |  |  |  |  | 0.0000025 | 0.19 | Prostate |
|  |  |  |  |  | 0.0000069 | 0.085 | Thyroid |
| rs34463936 | 5p13.2 | 5 | 35850149 | IL7R | 2.90E-08 | -0.28 | Testis |
|  |  |  |  | LMBRD2 | 3.10E-07 | -0.13 | Lung |
|  |  |  |  | SPEF2 | 0.00023 | 0.09 | Cells - Cultured fibroblasts |
| rs10213865 | 5p13.2 | 5 | 35857850 | IL7R | 5.60E-08 | -0.26 | Testis |
|  |  |  |  | LMBRD2 | 0.0000065 | -0.11 | Lung |
|  |  |  |  | SPEF2 | 0.00012 | 0.093 | Cells - Cultured fibroblasts |
|  |  |  |  |  | 0.00013 | 0.21 | Brain - Cortex |
|  |  |  |  | UGT3A1 | 0.000064 | 0.23 | Pituitary |
| rs77973332 | 10q21.3 | 10 | 64881009 | NRBF2 | 0.000022 | 0.15 | Whole Blood |
|  |  |  |  |  | 0.00011 | -0.41 | Thyroid |
| rs57943165 | 10q21.3 | 10 | 65362966 | NRBF2 | 0.000013 | 0.41 | Spleen |
|  |  |  |  |  | 0.000039 | -0.41 | Thyroid |
|  |  |  |  |  | 0.00014 | -0.23 | Cells - Cultured fibroblasts |
| rs1250564 | 10q22.3 | 10 | 81047342 | PPIF | 0.0000035 | 0.098 | Whole Blood |
|  |  |  |  | MBL1P | 0.0000076 | -0.17 | Skin - Sun Exposed (Lower leg) |
| rs7895695 | 10q24.1 | 10 | 99167255 | ARHGAP19 | 0.000034 | 0.13 | Adipose - Visceral (Omentum) |
|  |  |  |  |  | 0.000059 | 0.12 | Adipose - Subcutaneous |
|  |  |  |  | EXOSC1 | 1.70E-10 | 0.15 | Artery - Tibial |
|  |  |  |  |  | 7.60E-08 | 0.12 | Muscle - Skeletal |
|  |  |  |  |  | 4.90E-07 | 0.23 | Prostate |
|  |  |  |  |  | 6.80E-07 | 0.14 | Thyroid |
|  |  |  |  |  | 0.0000021 | 0.23 | Testis |
|  |  |  |  |  | 0.0000088 | 0.17 | Pancreas |
|  |  |  |  |  | 0.000016 | 0.17 | Heart - Atrial Appendage |
|  |  |  |  |  | 0.000017 | 0.11 | Cells - EBV-transformed lymphocytes |
|  |  |  |  |  | 0.000034 | 0.12 | Stomach |
|  |  |  |  |  | 0.000042 | 0.22 | Adrenal Gland |
|  |  |  |  |  | 0.000065 | 0.13 | Adipose - Subcutaneous |
|  |  |  |  |  | 0.00017 | 0.11 | Skin - Not Sun Exposed (Suprapubic) |
|  |  |  |  | FRAT1 | 0.000038 | -0.097 | Skin - Sun Exposed (Lower leg) |
|  |  |  |  |  | 0.00013 | -0.12 | Skin - Not Sun Exposed (Suprapubic) |
|  |  |  |  | FRAT2 | 2.00E-12 | 0.11 | Whole Blood |
|  |  |  |  |  | 3.70E-11 | 0.16 | Muscle - Skeletal |
|  |  |  |  |  | 4.40E-08 | 0.15 | Esophagus - Mucosa |
|  |  |  |  |  | 3.00E-07 | 0.16 | Spleen |
|  |  |  |  | MMS19 | 0.000087 | -0.15 | Brain - Cortex |
|  |  |  |  | PGAM1 | 0.0000097 | 0.1 | Nerve - Tibial |
|  |  |  |  | RRP12 | 4.80E-63 | 0.35 | Muscle - Skeletal |
|  |  |  |  |  | 7.80E-59 | 0.5 | Heart - Left Ventricle |
|  |  |  |  |  | 2.20E-58 | 0.39 | Whole Blood |
|  |  |  |  |  | 9.30E-50 | 0.49 | Heart - Atrial Appendage |
|  |  |  |  |  | 4.30E-23 | 0.19 | Skin - Sun Exposed (Lower leg) |
|  |  |  |  |  | 4.10E-22 | 0.29 | Brain - Caudate (basal ganglia) |
|  |  |  |  |  | 4.80E-22 | 0.29 | Pancreas |
|  |  |  |  |  | 4.70E-21 | 0.21 | Lung |
|  |  |  |  |  | 3.80E-16 | 0.18 | Skin - Not Sun Exposed (Suprapubic) |
|  |  |  |  |  | 5.30E-15 | 0.26 | Artery - Aorta |
|  |  |  |  |  | 1.00E-14 | 0.29 | Pituitary |
|  |  |  |  |  | 1.10E-14 | 0.24 | Brain - Nucleus accumbens (basal ganglia) |
|  |  |  |  |  | 2.30E-14 | 0.37 | Spleen |
|  |  |  |  |  | 4.80E-14 | 0.19 | Artery - Tibial |
|  |  |  |  |  | 1.70E-13 | 0.21 | Stomach |
|  |  |  |  |  | 9.90E-13 | 0.2 | Cells - EBV-transformed lymphocytes |
|  |  |  |  |  | 3.80E-12 | 0.49 | Brain - Cerebellum |
|  |  |  |  |  | 6.10E-12 | 0.16 | Breast - Mammary Tissue |
|  |  |  |  |  | 1.80E-11 | 0.24 | Adrenal Gland |
|  |  |  |  |  | 1.80E-11 | 0.23 | Brain - Putamen (basal ganglia) |
|  |  |  |  |  | 2.20E-11 | 0.19 | Colon - Transverse |
|  |  |  |  |  | 6.70E-11 | 0.25 | Testis |
|  |  |  |  |  | 1.70E-10 | 0.38 | Brain - Cerebellar Hemisphere |
|  |  |  |  |  | 7.60E-10 | 0.16 | Esophagus - Muscularis |
|  |  |  |  |  | 1.30E-09 | 0.23 | Brain - Cortex |
|  |  |  |  |  | 2.00E-09 | 0.21 | Brain - Frontal Cortex (BA9) |
|  |  |  |  |  | 9.70E-08 | 0.11 | Adipose - Subcutaneous |
|  |  |  |  |  | 0.0000021 | 0.19 | Artery - Coronary |
|  |  |  |  |  | 0.000004 | 0.085 | Nerve - Tibial |
|  |  |  |  |  | 0.0000046 | 0.18 | Brain - Anterior qcingulate cortex (BA24) |
|  |  |  |  |  | 0.000006 | 0.084 | Adipose - Visceral (Omentum) |
|  |  |  |  |  | 0.000013 | 0.15 | Esophagus - Gastroesophageal Junction |
|  |  |  |  |  | 0.000019 | 0.15 | Colon - Sigmoid |
|  |  |  |  |  | 0.000021 | 0.14 | Prostate |
|  |  |  |  |  | 0.00016 | 0.073 | Esophagus - Mucosa |
|  |  |  |  | SLIT1 | 1.20E-08 | -0.19 | Whole Blood |
|  |  |  |  |  | 3.70E-08 | -0.19 | Nerve - Tibial |
|  |  |  |  |  | 0.0000061 | -0.22 | Esophagus - Muscularis |
|  |  |  |  |  | 0.000029 | -0.25 | Artery - Aorta |
|  |  |  |  |  | 0.000037 | -0.2 | Skin - Sun Exposed (Lower leg) |
|  |  |  |  |  | 0.000064 | -0.18 | Artery - Tibial |
| rs694739 | 11q13.1 | 11 | 64097233 | ATL3 | 0.00012 | 0.068 | Muscle - Skeletal |
|  |  |  |  | BAD | 0.00016 | 0.081 | Esophagus - Mucosa |
|  |  |  |  | CATSPERZ | 0.00017 | 0.17 | Thyroid |
|  |  |  |  | CCDC88B | 2.00E-44 | -1 | Brain - Cerebellum |
|  |  |  |  |  | 6.60E-44 | -0.8 | Brain - Cerebellar Hemisphere |
|  |  |  |  |  | 1.30E-36 | -0.46 | Esophagus - Mucosa |
|  |  |  |  |  | 1.30E-36 | -0.33 | Esophagus - Muscularis |
|  |  |  |  |  | 2.80E-30 | -0.27 | Skin - Sun Exposed (Lower leg) |
|  |  |  |  |  | 9.60E-25 | -0.37 | Esophagus - Gastroesophageal Junction |
|  |  |  |  |  | 2.10E-21 | -0.29 | Colon - Sigmoid |
|  |  |  |  |  | 2.30E-20 | -0.25 | Colon - Transverse |
|  |  |  |  |  | 2.50E-20 | -0.52 | Brain - Cortex |
|  |  |  |  |  | 8.50E-20 | -0.43 | Brain - Frontal Cortex (BA9) |
|  |  |  |  |  | 4.40E-18 | -0.33 | Brain - Caudate (basal ganglia) |
|  |  |  |  |  | 3.80E-16 | -0.22 | Skin - Not Sun Exposed (Suprapubic) |
|  |  |  |  |  | 6.00E-16 | -0.35 | Brain - Nucleus accumbens (basal ganglia) |
|  |  |  |  |  | 7.40E-16 | -0.37 | Brain - Putamen (basal ganglia) |
|  |  |  |  |  | 4.50E-13 | -0.28 | Pituitary |
|  |  |  |  |  | 4.90E-12 | -0.28 | Brain - Hippocampus |
|  |  |  |  |  | 2.40E-11 | -0.12 | Thyroid |
|  |  |  |  |  | 1.60E-10 | -0.16 | Artery - Aorta |
|  |  |  |  |  | 2.50E-10 | -0.098 | Whole Blood |
|  |  |  |  |  | 2.70E-09 | -0.12 | Artery - Tibial |
|  |  |  |  |  | 4.50E-09 | -0.12 | Adipose - Subcutaneous |
|  |  |  |  |  | 6.20E-09 | -0.19 | Stomach |
|  |  |  |  |  | 6.40E-09 | -0.19 | Heart - Atrial Appendage |
|  |  |  |  |  | 1.50E-08 | -0.26 | Brain - Anterior qcingulate cortex (BA24) |
|  |  |  |  |  | 0.0000041 | -0.1 | Nerve - Tibial |
|  |  |  |  |  | 0.000027 | -0.091 | Adipose - Visceral (Omentum) |
|  |  |  |  |  | 0.000028 | -0.13 | Prostate |
|  |  |  |  |  | 0.000049 | -0.31 | Ovary |
|  |  |  |  | DNAJC4 | 0.00018 | 0.054 | Testis |
|  |  |  |  |  | 0.0003 | -0.08 | Thyroid |
|  |  |  |  | ENSG00000236935 | 2.50E-111 | 0.83 | Whole Blood |
|  |  |  |  |  | 5.20E-52 | 0.93 | Cells - EBV-transformed lymphocytes |
|  |  |  |  |  | 1.00E-37 | 0.84 | Spleen |
|  |  |  |  |  | 2.70E-20 | 0.44 | Lung |
|  |  |  |  |  | 2.90E-15 | 0.42 | Adipose - Visceral (Omentum) |
|  |  |  |  |  | 1.60E-11 | 0.51 | Brain - Caudate (basal ganglia) |
|  |  |  |  |  | 3.30E-11 | 0.31 | Thyroid |
|  |  |  |  |  | 3.60E-11 | 0.47 | Brain - Cerebellum |
|  |  |  |  |  | 1.60E-10 | 0.36 | Nerve - Tibial |
|  |  |  |  |  | 1.10E-09 | 0.37 | Brain - Cerebellar Hemisphere |
|  |  |  |  |  | 8.50E-09 | 0.31 | Adipose - Subcutaneous |
|  |  |  |  |  | 2.30E-07 | 0.32 | Breast - Mammary Tissue |
|  |  |  |  |  | 0.0000021 | 0.33 | Small Intestine - Terminal Ileum |
|  |  |  |  |  | 0.0000033 | 0.5 | Minor Salivary Gland |
|  |  |  |  |  | 0.000004 | 0.31 | Testis |
|  |  |  |  |  | 0.0000052 | 0.44 | Brain - Cortex |
|  |  |  |  |  | 0.0000078 | 0.25 | Esophagus - Mucosa |
|  |  |  |  |  | 0.000073 | 0.26 | Stomach |
|  |  |  |  | ENSG00000257086 | 0.00004 | -0.43 | Brain - Cerebellum |
|  |  |  |  | ENSG00000288852 | 5.90E-15 | 0.26 | Muscle - Skeletal |
|  |  |  |  |  | 1.40E-11 | 0.28 | Esophagus - Muscularis |
|  |  |  |  |  | 1.70E-10 | 0.22 | Skin - Sun Exposed (Lower leg) |
|  |  |  |  |  | 6.40E-10 | 0.26 | Breast - Mammary Tissue |
|  |  |  |  |  | 8.00E-10 | 0.25 | Thyroid |
|  |  |  |  |  | 1.70E-09 | 0.42 | Brain - Nucleus accumbens (basal ganglia) |
|  |  |  |  |  | 6.40E-09 | 0.22 | Adipose - Subcutaneous |
|  |  |  |  |  | 1.20E-08 | 0.39 | Brain - Putamen (basal ganglia) |
|  |  |  |  |  | 4.90E-08 | 0.4 | Brain - Anterior qcingulate cortex (BA24) |
|  |  |  |  |  | 7.50E-08 | 0.39 | Brain - Cerebellar Hemisphere |
|  |  |  |  |  | 1.90E-07 | 0.32 | Brain - Frontal Cortex (BA9) |
|  |  |  |  |  | 2.00E-07 | 0.35 | Brain - Cortex |
|  |  |  |  |  | 3.00E-07 | 0.22 | Lung |
|  |  |  |  |  | 3.60E-07 | 0.21 | Nerve - Tibial |
|  |  |  |  |  | 3.70E-07 | 0.19 | Skin - Not Sun Exposed (Suprapubic) |
|  |  |  |  |  | 4.10E-07 | 0.35 | Brain - Cerebellum |
|  |  |  |  |  | 4.70E-07 | 0.36 | Brain - Hippocampus |
|  |  |  |  |  | 6.30E-07 | 0.19 | Artery - Tibial |
|  |  |  |  |  | 6.30E-07 | 0.23 | Colon - Sigmoid |
|  |  |  |  |  | 0.0000025 | 0.16 | Cells - Cultured fibroblasts |
|  |  |  |  |  | 0.0000027 | 0.24 | Esophagus - Gastroesophageal Junction |
|  |  |  |  |  | 0.0000057 | 0.15 | Whole Blood |
|  |  |  |  |  | 0.0000071 | 0.3 | Liver |
|  |  |  |  |  | 0.0000084 | 0.23 | Heart - Atrial Appendage |
|  |  |  |  |  | 0.0000097 | 0.47 | Uterus |
|  |  |  |  |  | 0.000015 | 0.25 | Stomach |
|  |  |  |  |  | 0.000021 | 0.28 | Prostate |
|  |  |  |  |  | 0.000025 | 0.21 | Adipose - Visceral (Omentum) |
|  |  |  |  |  | 0.000031 | 0.21 | Colon - Transverse |
|  |  |  |  |  | 0.00022 | 0.17 | Esophagus - Mucosa |
|  |  |  |  | ENSG00000289486 | 3.20E-11 | 0.25 | Muscle - Skeletal |
|  |  |  |  |  | 3.70E-08 | 0.24 | Thyroid |
|  |  |  |  |  | 5.30E-08 | 0.33 | Artery - Aorta |
|  |  |  |  |  | 1.00E-07 | 0.26 | Esophagus - Muscularis |
|  |  |  |  |  | 1.80E-07 | 0.19 | Cells - Cultured fibroblasts |
|  |  |  |  |  | 0.0000023 | 0.23 | Artery - Tibial |
|  |  |  |  |  | 0.0000069 | 0.21 | Adipose - Subcutaneous |
|  |  |  |  |  | 0.0000074 | 0.26 | Pituitary |
|  |  |  |  |  | 0.0000087 | 0.4 | Brain - Anterior qcingulate cortex (BA24) |
|  |  |  |  |  | 0.000011 | 0.22 | Lung |
|  |  |  |  |  | 0.000012 | 0.26 | Heart - Atrial Appendage |
|  |  |  |  |  | 0.000014 | 0.26 | Esophagus - Gastroesophageal Junction |
|  |  |  |  |  | 0.000034 | 0.44 | Ovary |
|  |  |  |  |  | 0.000038 | 0.19 | Skin - Sun Exposed (Lower leg) |
|  |  |  |  |  | 0.000042 | 0.23 | Esophagus - Mucosa |
|  |  |  |  |  | 0.000054 | 0.36 | Brain - Cerebellum |
|  |  |  |  |  | 0.00013 | 0.2 | Nerve - Tibial |
|  |  |  |  | FERMT3 | 0.00024 | -0.075 | Skin - Not Sun Exposed (Suprapubic) |
|  |  |  |  | GPR137 | 0.000044 | -0.071 | Whole Blood |
|  |  |  |  | PGAM1P8 | 0.00017 | 0.22 | Testis |
|  |  |  |  | PLCB3 | 4.80E-10 | 0.16 | Esophagus - Mucosa |
|  |  |  |  |  | 8.50E-07 | -0.34 | Brain - Cerebellar Hemisphere |
|  |  |  |  |  | 0.000069 | -0.27 | Brain - Cerebellum |
|  |  |  |  |  | 0.00026 | 0.064 | Skin - Sun Exposed (Lower leg) |
|  |  |  |  |  | 0.00029 | 0.069 | Nerve - Tibial |
|  |  |  |  | PPP1R14B | 5.30E-62 | -0.36 | Artery - Tibial |
|  |  |  |  |  | 3.90E-32 | -0.26 | Muscle - Skeletal |
|  |  |  |  |  | 6.00E-30 | -0.32 | Artery - Aorta |
|  |  |  |  |  | 1.40E-16 | -0.25 | Esophagus - Gastroesophageal Junction |
|  |  |  |  |  | 1.70E-15 | -0.21 | Colon - Sigmoid |
|  |  |  |  |  | 5.80E-15 | -0.16 | Esophagus - Muscularis |
|  |  |  |  |  | 3.40E-14 | -0.12 | Skin - Sun Exposed (Lower leg) |
|  |  |  |  |  | 3.90E-14 | -0.21 | Brain - Nucleus accumbens (basal ganglia) |
|  |  |  |  |  | 1.20E-13 | -0.23 | Stomach |
|  |  |  |  |  | 3.80E-13 | -0.14 | Thyroid |
|  |  |  |  |  | 4.60E-12 | -0.33 | Pancreas |
|  |  |  |  |  | 8.50E-12 | -0.18 | Heart - Left Ventricle |
|  |  |  |  |  | 2.00E-11 | -0.12 | Skin - Not Sun Exposed (Suprapubic) |
|  |  |  |  |  | 1.10E-10 | -0.18 | Heart - Atrial Appendage |
|  |  |  |  |  | 1.30E-10 | -0.13 | Adipose - Visceral (Omentum) |
|  |  |  |  |  | 1.20E-09 | -0.23 | Brain - Cerebellar Hemisphere |
|  |  |  |  |  | 6.00E-09 | -0.19 | Brain - Hypothalamus |
|  |  |  |  |  | 6.50E-09 | -0.19 | Brain - Cortex |
|  |  |  |  |  | 6.60E-09 | -0.33 | Brain - Substantia nigra |
|  |  |  |  |  | 7.70E-09 | -0.24 | Artery - Coronary |
|  |  |  |  |  | 1.30E-08 | -0.21 | Adrenal Gland |
|  |  |  |  |  | 2.80E-08 | -0.24 | Brain - Cerebellum |
|  |  |  |  |  | 8.30E-08 | -0.2 | Pituitary |
|  |  |  |  |  | 7.60E-07 | -0.11 | Adipose - Subcutaneous |
|  |  |  |  |  | 0.0000016 | -0.1 | Breast - Mammary Tissue |
|  |  |  |  |  | 0.0000017 | -0.12 | Brain - Anterior qcingulate cortex (BA24) |
|  |  |  |  |  | 0.000012 | -0.19 | Minor Salivary Gland |
|  |  |  |  |  | 0.000012 | -0.15 | Brain - Hippocampus |
|  |  |  |  |  | 0.000013 | -0.12 | Brain - Frontal Cortex (BA9) |
|  |  |  |  |  | 0.000014 | -0.09 | Nerve - Tibial |
|  |  |  |  |  | 0.000015 | -0.12 | Brain - Putamen (basal ganglia) |
|  |  |  |  |  | 0.000029 | -0.17 | Prostate |
|  |  |  |  |  | 0.000063 | -0.08 | Colon - Transverse |
|  |  |  |  |  | 0.00014 | -0.1 | Brain - Caudate (basal ganglia) |
|  |  |  |  | PPP1R14B-AS1 | 3.80E-13 | -0.24 | Muscle - Skeletal |
|  |  |  |  |  | 6.70E-07 | -0.19 | Skin - Sun Exposed (Lower leg) |
|  |  |  |  |  | 0.0000072 | -0.19 | Breast - Mammary Tissue |
|  |  |  |  |  | 0.00019 | -0.18 | Artery - Tibial |
|  |  |  |  | PRDX5 | 0.000014 | 0.092 | Skin - Sun Exposed (Lower leg) |
|  |  |  |  | RPS6KA4 | 4.10E-08 | -0.1 | Skin - Sun Exposed (Lower leg) |
|  |  |  |  |  | 0.0000063 | 0.14 | Brain - Caudate (basal ganglia) |
|  |  |  |  |  | 0.000075 | -0.075 | Skin - Not Sun Exposed (Suprapubic) |
| rs12826560 | 12p13.2 | 12 | 10532965 | KLRC1 | 6.20E-19 | 0.35 | Whole Blood |
|  |  |  |  |  | 3.40E-12 | 0.46 | Lung |
|  |  |  |  |  | 4.80E-09 | 0.4 | Thyroid |
|  |  |  |  |  | 4.20E-07 | 0.32 | Esophagus - Mucosa |
|  |  |  |  |  | 0.000002 | 0.39 | Esophagus - Muscularis |
|  |  |  |  |  | 0.000002 | 0.51 | Small Intestine - Terminal Ileum |
|  |  |  |  |  | 0.0000042 | 0.32 | Skin - Not Sun Exposed (Suprapubic) |
|  |  |  |  |  | 0.00002 | 0.45 | Spleen |
|  |  |  |  |  | 0.000066 | 0.24 | Adipose - Subcutaneous |
|  |  |  |  | KLRC2 | 1.50E-25 | 1.1 | Brain - Caudate (basal ganglia) |
|  |  |  |  |  | 6.20E-24 | 1.3 | Brain - Spinal cord (cervical c-1) |
|  |  |  |  |  | 7.20E-24 | 1.1 | Brain - Frontal Cortex (BA9) |
|  |  |  |  |  | 3.70E-23 | 1.2 | Brain - Hippocampus |
|  |  |  |  |  | 7.00E-23 | 1.2 | Brain - Cortex |
|  |  |  |  |  | 8.20E-20 | 1.1 | Brain - Anterior qcingulate cortex (BA24) |
|  |  |  |  |  | 1.10E-19 | 1.1 | Brain - Putamen (basal ganglia) |
|  |  |  |  |  | 6.90E-19 | 1.1 | Brain - Hypothalamus |
|  |  |  |  |  | 2.10E-18 | 1 | Brain - Nucleus accumbens (basal ganglia) |
|  |  |  |  |  | 2.90E-17 | 1.1 | Brain - Amygdala |
|  |  |  |  |  | 5.40E-17 | 0.96 | Brain - Cerebellar Hemisphere |
|  |  |  |  |  | 1.50E-12 | 0.89 | Brain - Cerebellum |
|  |  |  |  |  | 5.00E-12 | 1.1 | Brain - Substantia nigra |
|  |  |  |  |  | 5.20E-11 | 0.26 | Whole Blood |
|  |  |  |  |  | 0.00023 | 0.26 | Nerve - Tibial |
|  |  |  |  | KLRC3 | 1.30E-14 | 0.65 | Brain - Frontal Cortex (BA9) |
|  |  |  |  |  | 1.30E-14 | 0.27 | Whole Blood |
|  |  |  |  |  | 1.20E-12 | 0.85 | Brain - Spinal cord (cervical c-1) |
|  |  |  |  |  | 7.20E-11 | 0.56 | Brain - Caudate (basal ganglia) |
|  |  |  |  |  | 1.10E-10 | 0.63 | Brain - Cortex |
|  |  |  |  |  | 5.00E-10 | 0.53 | Brain - Anterior qcingulate cortex (BA24) |
|  |  |  |  |  | 7.00E-10 | 0.49 | Brain - Hypothalamus |
|  |  |  |  |  | 2.40E-09 | 0.52 | Brain - Hippocampus |
|  |  |  |  |  | 3.20E-07 | 0.34 | Lung |
|  |  |  |  |  | 5.40E-07 | 0.3 | Adipose - Subcutaneous |
|  |  |  |  |  | 6.20E-07 | 0.39 | Brain - Nucleus accumbens (basal ganglia) |
|  |  |  |  |  | 0.000001 | 0.62 | Brain - Cerebellum |
|  |  |  |  |  | 0.0000011 | 0.53 | Brain - Amygdala |
|  |  |  |  |  | 0.0000042 | 0.55 | Brain - Substantia nigra |
|  |  |  |  |  | 0.0000098 | 0.45 | Brain - Putamen (basal ganglia) |
|  |  |  |  |  | 0.000013 | 0.42 | Spleen |
|  |  |  |  | KLRC4 | 8.10E-13 | 0.78 | Brain - Cortex |
|  |  |  |  |  | 2.50E-12 | 0.69 | Brain - Frontal Cortex (BA9) |
|  |  |  |  |  | 4.10E-12 | 0.66 | Brain - Hippocampus |
|  |  |  |  |  | 1.00E-09 | 0.74 | Brain - Amygdala |
|  |  |  |  |  | 4.50E-09 | 0.59 | Brain - Hypothalamus |
|  |  |  |  |  | 2.00E-08 | 0.54 | Brain - Anterior qcingulate cortex (BA24) |
|  |  |  |  |  | 6.20E-08 | 0.6 | Brain - Putamen (basal ganglia) |
|  |  |  |  |  | 6.50E-08 | 0.31 | Adipose - Subcutaneous |
|  |  |  |  |  | 7.10E-08 | 0.56 | Brain - Nucleus accumbens (basal ganglia) |
|  |  |  |  |  | 2.10E-07 | 0.44 | Brain - Caudate (basal ganglia) |
|  |  |  |  |  | 0.0000028 | 0.6 | Brain - Cerebellum |
|  |  |  |  |  | 0.0000029 | -0.23 | Thyroid |
|  |  |  |  |  | 0.0000035 | 0.59 | Brain - Cerebellar Hemisphere |
|  |  |  |  |  | 0.0000091 | 0.59 | Brain - Substantia nigra |
|  |  |  |  |  | 0.000027 | -0.42 | Pituitary |
|  |  |  |  | KLRK1 | 5.20E-14 | 0.36 | Adipose - Subcutaneous |
|  |  |  |  |  | 1.80E-10 | 0.56 | Esophagus - Gastroesophageal Junction |
|  |  |  |  |  | 8.00E-09 | 0.41 | Esophagus - Muscularis |
|  |  |  |  |  | 0.0000025 | 0.47 | Brain - Hippocampus |
|  |  |  |  |  | 0.0000086 | 0.27 | Muscle - Skeletal |
|  |  |  |  |  | 0.000018 | 0.3 | Colon - Sigmoid |
|  |  |  |  |  | 0.000029 | 0.31 | Heart - Left Ventricle |
|  |  |  |  |  | 0.000042 | 0.076 | Whole Blood |
|  |  |  |  |  | 0.000043 | 0.21 | Skin - Sun Exposed (Lower leg) |
|  |  |  |  |  | 0.000044 | 0.24 | Spleen |
|  |  |  |  |  | 0.000079 | 0.15 | Adipose - Visceral (Omentum) |
|  |  |  |  | KLRK1-AS1 | 3.50E-09 | 0.64 | Brain - Cortex |
|  |  |  |  |  | 1.70E-07 | 0.28 | Adipose - Subcutaneous |
|  |  |  |  |  | 2.30E-07 | 0.46 | Brain - Caudate (basal ganglia) |
|  |  |  |  |  | 0.0000039 | 0.41 | Brain - Frontal Cortex (BA9) |
|  |  |  |  |  | 0.0000077 | 0.43 | Adrenal Gland |
|  |  |  |  |  | 0.000036 | 0.42 | Brain - Nucleus accumbens (basal ganglia) |
|  |  |  |  | YBX3 | 0.00001 | -0.098 | Lung |
| rs6575931 | 14q32.32 | 14 | 103301574 | TRAF3 | 1.80E-08 | 0.2 | Brain - Cortex |
|  |  |  |  |  | 8.10E-08 | 0.18 | Thyroid |
|  |  |  |  |  | 1.50E-07 | 0.19 | Brain - Frontal Cortex (BA9) |
|  |  |  |  |  | 2.40E-07 | 0.24 | Brain - Hypothalamus |
|  |  |  |  |  | 5.30E-07 | 0.3 | Brain - Spinal cord (cervical c-1) |
|  |  |  |  |  | 0.0000014 | 0.15 | Esophagus - Mucosa |
|  |  |  |  |  | 0.0000024 | 0.17 | Spleen |
|  |  |  |  |  | 0.000012 | 0.22 | Brain - Anterior qcingulate cortex (BA24) |
|  |  |  |  |  | 0.00013 | -0.098 | Artery - Tibial |
| rs3852751 | 16p13.2 | 16 | 10347651 | None | None | None | None |
| rs36001636 | 16q23.2 | 16 | 79349806 | None | None | None | None |
| rs1893592 | 21q22.3 | 21 | 43855067 | RSPH1 | 2.90E-07 | 0.23 | Brain - Putamen (basal ganglia) |
|  |  |  |  |  | 0.000004 | 0.16 | Brain - Caudate (basal ganglia) |
|  |  |  |  | UBASH3A | 3.20E-48 | 0.25 | Whole Blood |
|  |  |  |  |  | 7.90E-39 | 0.4 | Thyroid |
|  |  |  |  |  | 3.50E-29 | 0.33 | Esophagus - Mucosa |
|  |  |  |  |  | 4.90E-29 | 0.58 | Spleen |
|  |  |  |  |  | 3.40E-26 | 0.29 | Lung |
|  |  |  |  |  | 6.70E-25 | 0.37 | Stomach |
|  |  |  |  |  | 1.20E-23 | 0.48 | Nerve - Tibial |
|  |  |  |  |  | 1.20E-21 | 0.26 | Colon - Transverse |
|  |  |  |  |  | 2.60E-21 | 0.35 | Adipose - Visceral (Omentum) |
|  |  |  |  |  | 6.40E-18 | 0.36 | Small Intestine - Terminal Ileum |
|  |  |  |  |  | 5.40E-17 | 0.4 | Prostate |
|  |  |  |  |  | 9.90E-17 | 0.33 | Skin - Not Sun Exposed (Suprapubic) |
|  |  |  |  |  | 2.10E-15 | 0.31 | Adipose - Subcutaneous |
|  |  |  |  |  | 2.80E-13 | 0.41 | Esophagus - Muscularis |
|  |  |  |  |  | 1.50E-11 | 0.29 | Breast - Mammary Tissue |
|  |  |  |  |  | 3.20E-11 | 0.39 | Heart - Atrial Appendage |
|  |  |  |  |  | 3.80E-11 | 0.58 | Minor Salivary Gland |
|  |  |  |  |  | 5.40E-11 | 0.38 | Vagina |
|  |  |  |  |  | 2.10E-10 | 0.44 | Liver |
|  |  |  |  |  | 4.10E-09 | 0.23 | Skin - Sun Exposed (Lower leg) |
|  |  |  |  |  | 1.00E-08 | 0.38 | Esophagus - Gastroesophageal Junction |
|  |  |  |  |  | 3.80E-08 | 0.3 | Artery - Coronary |
|  |  |  |  |  | 4.30E-08 | 0.47 | Ovary |
|  |  |  |  |  | 6.50E-08 | 0.23 | Artery - Aorta |
|  |  |  |  |  | 2.30E-07 | 0.37 | Adrenal Gland |
|  |  |  |  |  | 0.0000038 | 0.25 | Colon - Sigmoid |
|  |  |  |  |  | 0.0000073 | 0.3 | Pituitary |
|  |  |  |  |  | 0.000025 | 0.16 | Artery - Tibial |

SNP: Reference SNP identifier (rsID)

Cytogenetic band: chromosomal band location

CHR: chromosome

BP: base pair position on GRCh37

Gene: gene name

P-value: association significance

NES: normalized effect size

Tissue: GTEx-derived tissue

Table S2. Single-Cell eQTLs for novel loci identified in the meta-analysis and conjunctional FDR analysis

| **SNP** | **QTLtype** | **cellTypeName** | **Gene** | ***P*-value** | **beta** | **se** | **PMID** |
| --- | --- | --- | --- | --- | --- | --- | --- |
| rs1763839 | Cell-type-specific eQTL | CD8+ Memory | RERE | 0.000235297 | 0.19403 | 0.05179 | 37558883 |
|  | Cell-type-specific eQTL | CD8+ Memory | RERE | 0.000235297 | 0.19403 | 0.05179 | 37558883 |
|  | Cell-type-specific eQTL | Excitatory Neurons | SLC45A1 | 0.000104166 | -0.1185 | 0.03054 | 35915177 |
|  | Cell-type-specific eQTL | Naive Regulatory T | RP5-1115A15.1 | 3.47E-07 | 0.84 | 0.16484 | 35213211 |
|  | Cell-type-specific eQTL | Astrocytes | RERE | 3.24E-11 | 0.33563 | 0.05058 | 35915177 |
| rs75873622 | Response eQTL | Dopaminergic Neurons | ALDH4A1 | 8.76E-05 | 0.39296 | 0.10018 | 33664506 |
| rs7524764 | Cell-type-specific eQTL | Naive B Cell | FCRL3 | 5.42E-06 | 0.5878 | 0.12569 | 37558883 |
|  | Cell-type-specific eQTL | Naive B Cell | FCRL3 | 5.33E-05 | 0.55877 | 0.13532 | 37558883 |
|  | Cell-type-specific eQTL | Naive B Cell | FCRL3 | 5.33E-05 | 0.55877 | 0.13532 | 37558883 |
|  | Cell-type-specific eQTL | Regulatory T (Treg) | FCRL3 | 5.43E-06 | 0.63027 | 0.13487 | 37558883 |
|  | Cell-type-specific eQTL | Regulatory T (Treg) | FCRL3 | 1.79E-06 | 0.82686 | 0.16817 | 37558883 |
|  | Cell-type-specific eQTL | Naive B Cell | FCRL3 | 5.42E-06 | 0.5878 | 0.12569 | 37558883 |
|  | Cell-type-specific eQTL | Natural Killer Cell (NK) | FCRL3 | 4.73E-07 | 0.64924 | 0.12449 | 37558883 |
|  | Cell-type-specific eQTL | Natural Killer Cell (NK) | FCRL3 | 4.73E-07 | 0.64924 | 0.12449 | 37558883 |
|  | Cell-type-specific eQTL | Natural Killer Cell (NK) | FCRL3 | 9.89E-08 | 0.60986 | 0.11018 | 37558883 |
|  | Cell-type-specific eQTL | Natural Killer Cell (NK) | FCRL3 | 9.89E-08 | 0.60986 | 0.11018 | 37558883 |
|  | Cell-type-specific eQTL | CD8+ Memory | FCRL3 | 2.23E-06 | 0.4323 | 0.08863 | 37558883 |
|  | Cell-type-specific eQTL | Natural Killer Cell (NK) | FCRL3 | 8.19E-06 | 0.49896 | 0.10881 | 37558883 |
|  | Cell-type-specific eQTL | Natural Killer Cell (NK) | FCRL3 | 3.63E-06 | 0.39103 | 0.082 | 37558883 |
|  | Cell-type-specific eQTL | Natural Killer Cell (NK) | FCRL3 | 3.63E-06 | 0.39103 | 0.082 | 37558883 |
|  | Cell-type-specific eQTL | B Cell | FCRL3 | 0.000100623 | 0.25202 | 0.06348 | 37558883 |
|  | Cell-type-specific eQTL | B Cell | FCRL3 | 0.000100623 | 0.25202 | 0.06348 | 37558883 |
|  | Cell-type-specific eQTL | B Cell | FCRL3 | 4.09E-06 | 0.30628 | 0.06461 | 37558883 |
|  | Cell-type-specific eQTL | B Cell | FCRL3 | 4.09E-06 | 0.30628 | 0.06461 | 37558883 |
|  | Cell-type-specific eQTL | Regulatory T (Treg) | FCRL3 | 5.43E-06 | 0.63027 | 0.13487 | 37558883 |
|  | Cell-type-specific eQTL | Regulatory T (Treg) | FCRL3 | 2.00E-08 | 0.93926 | 0.14855 | 35618845 |
|  | Cell-type-specific eQTL | CD4+ Central Memory T | FCRL3 | 0.00083914 | 0.5384 | 0.15463 | 35618845 |
|  | Cell-type-specific eQTL | Regulatory T (Treg) | FCRL3 | 1.79E-06 | 0.82686 | 0.16817 | 37558883 |
|  | Cell-type-specific eQTL | CD8+ T Cell | FCRL3 | 0.000213927 | 0.62632 | 0.16604 | 37558883 |
|  | Cell-type-specific eQTL | CD8+ T Cell | FCRL3 | 0.000213927 | 0.62632 | 0.16604 | 37558883 |
|  | Cell-type-specific eQTL | CD8+ Memory | FCRL3 | 1.35E-07 | 0.70223 | 0.12844 | 37558883 |
|  | Cell-type-specific eQTL | CD8+ Memory | FCRL3 | 1.35E-07 | 0.70223 | 0.12844 | 37558883 |
|  | Cell-type-specific eQTL | CD8+ T Cell | FCRL3 | 0.000313297 | 0.27095 | 0.07386 | 37558883 |
|  | Cell-type-specific eQTL | CD8+ T Cell | FCRL3 | 0.000313297 | 0.27095 | 0.07386 | 37558883 |
|  | Cell-type-specific eQTL | CD8+ T Cell | FCRL3 | 7.95E-05 | 0.41404 | 0.10275 | 37558883 |
|  | Cell-type-specific eQTL | CD4 Memory | FCRL3 | 1.41E-06 | 0.85534 | 0.16255 | 35618845 |
|  | Cell-type-specific eQTL | CD4 Memory | FCRL3 | 4.86E-05 | 0.64229 | 0.14871 | 35618845 |
|  | Cell-type-specific eQTL | CD4+ T Cell | FCRL3 | 5.82E-05 | 0.33669 | 0.08201 | 37558883 |
|  | Cell-type-specific eQTL | CD4+ T Cell | FCRL3 | 5.82E-05 | 0.33669 | 0.08201 | 37558883 |
|  | Cell-type-specific eQTL | CD8+ T Cell | FCRL3 | 8.94E-05 | 0.31195 | 0.07798 | 37558883 |
|  | Cell-type-specific eQTL | Natural Killer Cell (NK) | FCRL3 | 8.19E-06 | 0.49896 | 0.10881 | 37558883 |
|  | Cell-type-specific eQTL | CD8+ T Cell | FCRL3 | 8.94E-05 | 0.31195 | 0.07798 | 37558883 |
|  | Cell-type-specific eQTL | CD8+ Memory | FCRL3 | 2.23E-06 | 0.4323 | 0.08863 | 37558883 |
|  | Cell-type-specific eQTL | Natural Killer Cell (NK) | FCRL3 | 3.85E-05 | 1.17649 | 0.27956 | 37558883 |
|  | Cell-type-specific eQTL | Natural Killer Cell (NK) | FCRL3 | 3.85E-05 | 1.17649 | 0.27956 | 37558883 |
|  | Cell-type-specific eQTL | Naive Regulatory T | FCRL2 | 6.26E-09 | 1.31 | 0.22549 | 35213211 |
|  | Cell-type-specific eQTL | Memory Regulatory T | FCRL2 | 3.52E-09 | 1.34 | 0.22691 | 35213211 |
|  | Response eQTL | Natural Killer Cell (NK) | FCRL3 | 3.57E-05 | 4.1336 | 0.99995 | 35672358 |
|  | Response eQTL | CD8+ T Cell | FCRL3 | 2.52E-05 | 4.2132 | 0.99996 | 35672358 |
|  | Response eQTL | Natural Killer Cell (NK) | FCRL3 | 0.0001742 | 3.75376 | 0.99996 | 35672358 |
|  | Response eQTL | CD8+ T Cell | FCRL3 | 2.16E-05 | 4.2474 | 0.99998 | 35672358 |
|  | Response eQTL | CD8+ T Cell | FCRL3 | 2.42E-06 | 4.7148 | 1 | 35672358 |
|  | Cell-type-specific eQTL | CD8+ T Cell | FCRL3 | 7.95E-05 | 0.41404 | 0.10275 | 37558883 |
| rs3818813 | Cell-type-specific eQTL | Memory Regulatory T | FCRL2 | 2.37E-11 | 1.42 | 0.21253 | 35213211 |
|  | Response eQTL | CD4+ T Cell | FCRL1 | 0.000489743 | -0.26884 | 0.07711 | 35389781 |
|  | Response eQTL | CD8+ T Cell | FCRL3 | 0.000242 | 3.67058 | 0.99995 | 35672358 |
|  | Response eQTL | CD8+ T Cell | FCRL3 | 8.72E-06 | 4.4468 | 0.99996 | 35672358 |
|  | Cell-type-specific eQTL | CD8+ T Cell | FCRL3 | 2.78E-05 | 0.37074 | 0.08641 | 37558883 |
|  | Cell-type-specific eQTL | CD8+ T Cell | FCRL3 | 2.78E-05 | 0.37074 | 0.08641 | 37558883 |
|  | Cell-type-specific eQTL | CD8+ T Cell | FCRL3 | 4.55E-06 | 0.29542 | 0.06267 | 37558883 |
|  | Cell-type-specific eQTL | CD8+ T Cell | FCRL3 | 4.55E-06 | 0.29542 | 0.06267 | 37558883 |
|  | Cell-type-specific eQTL | CD8+ T Cell | FCRL3 | 1.70E-06 | 0.33196 | 0.06728 | 37558883 |
|  | Cell-type-specific eQTL | CD8+ T Cell | FCRL3 | 1.70E-06 | 0.33196 | 0.06728 | 37558883 |
|  | Cell-type-specific eQTL | CD4+ T Cell | FCRL1 | 1.19E-06 | 0.1753 | 0.03499 | 37558883 |
|  | Cell-type-specific eQTL | CD4+ T Cell | FCRL1 | 1.19E-06 | 0.1753 | 0.03499 | 37558883 |
|  | Cell-type-specific eQTL | CD4+ T Cell | FCRL3 | 1.19E-05 | 0.32906 | 0.07326 | 37558883 |
|  | Cell-type-specific eQTL | CD4+ T Cell | FCRL3 | 1.19E-05 | 0.32906 | 0.07326 | 37558883 |
|  | Cell-type-specific eQTL | CD4+ T Cell | FCRL1 | 9.51E-07 | 0.15637 | 0.03094 | 37558883 |
|  | Cell-type-specific eQTL | CD4+ T Cell | FCRL1 | 9.51E-07 | 0.15637 | 0.03094 | 37558883 |
|  | Cell-type-specific eQTL | CD4+ T Cell | FCRL3 | 1.80E-05 | 0.30838 | 0.07023 | 37558883 |
|  | Cell-type-specific eQTL | CD4+ T Cell | FCRL3 | 1.80E-05 | 0.30838 | 0.07023 | 37558883 |
|  | Cell-type-specific eQTL | CD4+ T Cell | FCRL3 | 4.11E-06 | 0.33531 | 0.07085 | 37558883 |
|  | Cell-type-specific eQTL | CD4+ T Cell | FCRL3 | 4.11E-06 | 0.33531 | 0.07085 | 37558883 |
|  | Cell-type-specific eQTL | Natural Killer Cell (NK) | FCRL3 | 6.43E-06 | 0.42562 | 0.09169 | 37558883 |
|  | Cell-type-specific eQTL | Natural Killer Cell (NK) | FCRL3 | 6.43E-06 | 0.42562 | 0.09169 | 37558883 |
|  | Cell-type-specific eQTL | Natural Killer Cell (NK) | FCRL3 | 0.000101998 | 0.28813 | 0.07262 | 37558883 |
|  | Cell-type-specific eQTL | Natural Killer Cell (NK) | FCRL3 | 0.000101998 | 0.28813 | 0.07262 | 37558883 |
|  | Cell-type-specific eQTL | B Cell | FCRL3 | 0.000264578 | 0.20609 | 0.05547 | 37558883 |
|  | Cell-type-specific eQTL | B Cell | FCRL3 | 0.000264578 | 0.20609 | 0.05547 | 37558883 |
|  | Cell-type-specific eQTL | B Cell | FCRL3 | 0.000536853 | 0.20241 | 0.05751 | 37558883 |
|  | Cell-type-specific eQTL | B Cell | FCRL3 | 0.000536853 | 0.20241 | 0.05751 | 37558883 |
|  | Cell-type-specific eQTL | Regulatory T (Treg) | FCRL1 | 2.19E-06 | 0.6263 | 0.12848 | 37558883 |
|  | Cell-type-specific eQTL | Regulatory T (Treg) | FCRL1 | 2.19E-06 | 0.6263 | 0.12848 | 37558883 |
|  | Cell-type-specific eQTL | Regulatory T (Treg) | FCRL3 | 8.31E-05 | 0.7289 | 0.18148 | 37558883 |
|  | Cell-type-specific eQTL | Regulatory T (Treg) | FCRL3 | 8.31E-05 | 0.7289 | 0.18148 | 37558883 |
|  | Cell-type-specific eQTL | Regulatory T (Treg) | FCRL1 | 2.23E-05 | 0.48119 | 0.1108 | 37558883 |
|  | Cell-type-specific eQTL | Regulatory T (Treg) | FCRL1 | 2.23E-05 | 0.48119 | 0.1108 | 37558883 |
|  | Cell-type-specific eQTL | Regulatory T (Treg) | FCRL3 | 3.49E-08 | 0.65258 | 0.11374 | 37558883 |
|  | Cell-type-specific eQTL | Regulatory T (Treg) | FCRL3 | 3.49E-08 | 0.65258 | 0.11374 | 37558883 |
|  | Cell-type-specific eQTL | Regulatory T (Treg) | FCRL3 | 7.49E-06 | 0.67007 | 0.1458 | 37558883 |
|  | Cell-type-specific eQTL | Regulatory T (Treg) | FCRL3 | 7.49E-06 | 0.67007 | 0.1458 | 37558883 |
|  | Cell-type-specific eQTL | CD8+ T Cell | FCRL3 | 1.16E-05 | 0.63344 | 0.14079 | 37558883 |
|  | Cell-type-specific eQTL | CD8+ T Cell | FCRL3 | 1.16E-05 | 0.63344 | 0.14079 | 37558883 |
|  | Cell-type-specific eQTL | CD8+ Memory | FCRL3 | 1.25E-07 | 0.61105 | 0.11146 | 37558883 |
|  | Cell-type-specific eQTL | CD8+ Memory | FCRL3 | 1.25E-07 | 0.61105 | 0.11146 | 37558883 |
|  | Cell-type-specific eQTL | CD8+ Memory | FCRL3 | 7.83E-06 | 0.35596 | 0.07749 | 37558883 |
|  | Cell-type-specific eQTL | CD8+ Memory | FCRL3 | 7.83E-06 | 0.35596 | 0.07749 | 37558883 |
|  | Cell-type-specific eQTL | CD8+ Memory | FCRL3 | 0.000203582 | 0.3164 | 0.08359 | 37558883 |
|  | Cell-type-specific eQTL | CD8+ Memory | FCRL3 | 0.000203582 | 0.3164 | 0.08359 | 37558883 |
|  | Cell-type-specific eQTL | Natural Killer Cell (NK) | FCRL3 | 3.52E-06 | 0.52331 | 0.10954 | 37558883 |
|  | Cell-type-specific eQTL | Natural Killer Cell (NK) | FCRL3 | 3.52E-06 | 0.52331 | 0.10954 | 37558883 |
|  | Cell-type-specific eQTL | CD4+ Central Memory T | FCRL3 | 0.000276411 | 0.58424 | 0.15298 | 35618845 |
|  | Cell-type-specific eQTL | Regulatory T (Treg) | FCRL3 | 4.75E-09 | 0.93637 | 0.14043 | 35618845 |
|  | Cell-type-specific eQTL | CD4 Memory | FCRL3 | 3.52E-06 | 0.82288 | 0.16372 | 35618845 |
|  | Cell-type-specific eQTL | CD4 Memory | FCRL3 | 0.000200474 | 0.59348 | 0.15155 | 35618845 |
|  | Cell-type-specific eQTL | Naive Regulatory T | FCRL2 | 1.27E-11 | 1.42 | 0.20969 | 35213211 |
|  | Cell-type-specific eQTL | Memory Regulatory T | FCRL3 | 1.25E-05 | 1.01 | 0.23119 | 35213211 |
|  | Cell-type-specific eQTL | Natural Killer Cell (NK) | FCRL3 | 1.37E-05 | 0.44404 | 0.09953 | 37558883 |
|  | Cell-type-specific eQTL | Natural Killer Cell (NK) | FCRL3 | 1.37E-05 | 0.44404 | 0.09953 | 37558883 |
|  | Cell-type-specific eQTL | Mucosal invariant T cell | FCRL3 | 2.96E-05 | 0.8289 | 0.19388 | 37558883 |
|  | Cell-type-specific eQTL | Mucosal invariant T cell | FCRL3 | 2.96E-05 | 0.8289 | 0.19388 | 37558883 |
| rs3771258 | Cell-type-specific eQTL | Excitatory Neurons | C2orf74 | 1.02E-08 | -0.46324 | 0.08088 | 35915177 |
|  | Cell-type-specific eQTL | Oligodendrocytes | C2orf74 | 2.97E-09 | -0.435 | 0.07332 | 35915177 |
|  | Cell-type-specific eQTL | Monocyte (Mono) | RP11-355B11.2 | 2.87E-06 | 0.68 | 0.1453 | 35213211 |
|  | Response eQTL | Ependymal-like 1 | PEX13 | 2.89E-05 | 0.24914 | 0.05958 | 33664506 |
| rs231805 | Cell-type-specific eQTL | CD4 Memory | CTLA4 | 0.00141779 | -0.23812 | 0.07174 | 35618845 |
| rs13429408 | Cell-type-specific eQTL | CD8+ Naive T | RP11-378A13.1 | 3.41E-07 | 0.82 | 0.16081 | 35213211 |
|  | Cell-type-specific eQTL | Monocyte (Mono) | RP11-378A13.1 | 1.20E-07 | 0.82 | 0.15488 | 35213211 |
|  | Cell-type-specific eQTL | Oligodendrocytes | ARPC2 | 4.49E-10 | -0.42637 | 0.06837 | 35915177 |
|  | Cell-type-specific eQTL | Microglia (Micro) | ARPC2 | 3.33E-05 | -0.36733 | 0.08852 | 35915177 |
|  | Cell-type-specific eQTL | CD4+ T Cell | RP11-378A13.1 | 3.29E-07 | 0.81 | 0.15864 | 35213211 |
|  | Cell-type-specific eQTL | Natural Killer Cell (NK) | RP11-378A13.1 | 1.12E-07 | 0.8 | 0.15076 | 35213211 |
|  | Cell-type-specific eQTL | Monocyte (Mono) | PNKD | 9.42E-10 | 0.9 | 0.14708 | 35213211 |
|  | Cell-type-specific eQTL | Monocyte (Mono) | RP11-378A13.1 | 1.88E-16 | 1.15 | 0.13974 | 35213211 |
|  | Cell-type-specific eQTL | CD4 Memory | TMBIM1 | 0.00153863 | -0.13582 | 0.04125 | 35618845 |
|  | Cell-type-specific eQTL | Naive Regulatory T | ARPC2 | 8.25E-07 | 0.76 | 0.15418 | 35213211 |
|  | Cell-type-specific eQTL | Naive Regulatory T | RP11-378A13.1 | 3.43E-12 | 1.04 | 0.14945 | 35213211 |
|  | Cell-type-specific eQTL | Memory Regulatory T | RP11-378A13.1 | 3.99E-12 | 1.04 | 0.14991 | 35213211 |
|  | Cell-type-specific eQTL | CD4+ T Cell | RP11-378A13.1 | 6.91E-12 | 1.05 | 0.15307 | 35213211 |
|  | Cell-type-specific eQTL | CD4+ T Cell | RP11-378A13.1 | 4.99E-10 | 0.94 | 0.15114 | 35213211 |
|  | Cell-type-specific eQTL | CD4+ T Cell | RP11-378A13.1 | 2.14E-12 | 1.04 | 0.14804 | 35213211 |
|  | Cell-type-specific eQTL | CD4+ T Cell | RP11-378A13.1 | 1.11E-08 | 0.93 | 0.1628 | 35213211 |
|  | Cell-type-specific eQTL | Inhibitory Neurons | ARPC2 | 3.30E-05 | -0.27589 | 0.06645 | 35915177 |
|  | Cell-type-specific eQTL | CD14+ Monocyte (CD14 Mono) | RP11-378A13.1 | 0.000398318 | 0.19893 | 0.05517 | 37558883 |
|  | Cell-type-specific eQTL | CD14+ Monocyte (CD14 Mono) | RP11-378A13.1 | 0.000398318 | 0.19893 | 0.05517 | 37558883 |
|  | Response eQTL | Astrocyte-like Cells | ARPC2 | 2.56E-06 | 0.26051 | 0.05539 | 33664506 |
|  | Cell-type-specific eQTL | Floor Plate Progenitors | ARPC2 | 5.52E-06 | 0.13067 | 0.02876 | 33664506 |
|  | Cell-type-specific eQTL | Ependymal-like 1 | ARPC2 | 2.90E-06 | 0.21194 | 0.04531 | 33664506 |
|  | Cell-type-specific eQTL | Proliferating Floor Plate Progenitors | TMBIM1 | 1.03E-10 | 0.35125 | 0.05435 | 33664506 |
|  | Cell-type-specific eQTL | Proliferating Floor Plate Progenitors | ARPC2 | 1.39E-16 | 0.21005 | 0.02541 | 33664506 |
|  | Cell-type-specific eQTL | Floor Plate Progenitors | BCS1L | 0.00089379 | 0.11242 | 0.03384 | 33664506 |
|  | Cell-type-specific eQTL | Floor Plate Progenitors | TMBIM1 | 8.75E-13 | 0.49686 | 0.0695 | 33664506 |
|  | Cell-type-specific eQTL | Floor Plate Progenitors | ARPC2 | 4.95E-14 | 0.18411 | 0.02444 | 33664506 |
|  | Cell-type-specific eQTL | CD14+ Monocyte (CD14 Mono) | PNKD | 3.31E-07 | 0.0675 | 0.01322 | 35389781 |
|  | Response eQTL | Monocyte (Mono) | TMBIM1 | 0.0003254 | -3.59419 | 1.00005 | 35672358 |
|  | Cell-type-specific eQTL | Serotonergic-like Neurons | ARPC2 | 0.000668077 | 0.20758 | 0.06101 | 33664506 |
|  | Response eQTL | Serotonergic-like Neurons | ARPC2 | 1.36E-09 | 0.4323 | 0.07133 | 33664506 |
|  | Cell-type-specific eQTL | Ependymal-like 1 | SLC11A1 | 0.000380466 | -0.22279 | 0.0627 | 33664506 |
|  | Cell-type-specific eQTL | Ependymal-like 1 | ARPC2 | 0.000182517 | 0.19973 | 0.05337 | 33664506 |
|  | Response eQTL | Ependymal-like 1 | ARPC2 | 2.25E-09 | 0.3429 | 0.05735 | 33664506 |
|  | Response eQTL | Dopaminergic Neurons | ARPC2 | 3.07E-10 | 0.32884 | 0.05224 | 33664506 |
|  | Cell-type-specific eQTL | CD8+ Naive T | ARPC2 | 6.43E-09 | 0.9 | 0.15503 | 35213211 |
|  | Cell-type-specific eQTL | CD8+ Naive T | RP11-378A13.1 | 3.07E-11 | 1 | 0.15053 | 35213211 |
|  | Cell-type-specific eQTL | CD4+ Naive T | RP11-378A13.1 | 4.90E-10 | 0.96 | 0.15428 | 35213211 |
|  | Cell-type-specific eQTL | CD4+ Naive T | ARPC2 | 8.58E-08 | 0.83 | 0.15501 | 35213211 |
|  | Cell-type-specific eQTL | CD4+ Naive T | RP11-378A13.1 | 3.14E-11 | 1.03 | 0.15513 | 35213211 |
|  | Cell-type-specific eQTL | Naive B Cell | ARPC2 | 4.24E-08 | 0.82 | 0.14962 | 35213211 |
|  | Cell-type-specific eQTL | Naive B Cell | RP11-378A13.1 | 2.56E-10 | 0.94 | 0.14865 | 35213211 |
|  | Cell-type-specific eQTL | CD16+ Monocyte (CD16 Mono) | PNKD | 0.000254496 | 0.08606 | 0.02353 | 35389781 |
|  | Cell-type-specific eQTL | Naive T Cell | AAMP | 0.000191027 | 0.27718 | 0.07053 | 35618845 |
|  | Cell-type-specific eQTL | Monocyte (Mono) | PNKD | 6.15E-07 | 0.75 | 0.15041 | 35213211 |
| rs34463936 | Response eQTL | Monocyte (Mono) | IL7R | 4.72E-06 | 4.5768 | 0.99999 | 35672358 |
|  | Cell-type-specific eQTL | Ependymal-like 1 | CAPSL | 0.000125128 | 0.12397 | 0.03232 | 33664506 |
|  | Cell-type-specific eQTL | CD14+ Monocyte (CD14 Mono) | IL7R | 4.16E-07 | 0.44898 | 0.0856 | 37558883 |
|  | Cell-type-specific eQTL | CD14+ Monocyte (CD14 Mono) | IL7R | 4.16E-07 | 0.44898 | 0.0856 | 37558883 |
|  | Cell-type-specific eQTL | CD14+ Monocyte (CD14 Mono) | IL7R | 3.99E-08 | 0.86991 | 0.15176 | 37558883 |
|  | Cell-type-specific eQTL | CD14+ Monocyte (CD14 Mono) | IL7R | 3.99E-08 | 0.86991 | 0.15176 | 37558883 |
|  | Cell-type-specific eQTL | CD14+ Monocyte (CD14 Mono) | IL7R | 5.18E-11 | 0.24032 | 0.03453 | 37558883 |
|  | Cell-type-specific eQTL | CD14+ Monocyte (CD14 Mono) | IL7R | 5.18E-11 | 0.24032 | 0.03453 | 37558883 |
|  | Cell-type-specific eQTL | CD4+ Naive T | IL7R | 0.000441233 | 0.11623 | 0.03253 | 37558883 |
|  | Cell-type-specific eQTL | CD4+ Naive T | IL7R | 0.000441233 | 0.11623 | 0.03253 | 37558883 |
|  | Cell-type-specific eQTL | CD4+ Naive T | IL7R | 1.56E-05 | 0.15962 | 0.03605 | 37558883 |
|  | Cell-type-specific eQTL | CD4+ Naive T | IL7R | 1.56E-05 | 0.15962 | 0.03605 | 37558883 |
|  | Cell-type-specific eQTL | Effector CD4+ T Cell | IL7R | 0.000242797 | 0.08802 | 0.02355 | 37558883 |
|  | Cell-type-specific eQTL | Effector CD4+ T Cell | IL7R | 0.000242797 | 0.08802 | 0.02355 | 37558883 |
|  | Cell-type-specific eQTL | CD16+ Monocyte (CD16 Mono) | IL7R | 7.53E-05 | 0.56463 | 0.13939 | 37558883 |
|  | Cell-type-specific eQTL | CD16+ Monocyte (CD16 Mono) | IL7R | 7.53E-05 | 0.56463 | 0.13939 | 37558883 |
|  | Cell-type-specific eQTL | Monocyte (Mono) | IL7R | 1.86E-11 | 0.24693 | 0.0346 | 37558883 |
|  | Cell-type-specific eQTL | CD8+ Naive T | IL7R | 0.000255725 | 0.14772 | 0.03965 | 37558883 |
|  | Cell-type-specific eQTL | CD8+ Naive T | IL7R | 0.000255725 | 0.14772 | 0.03965 | 37558883 |
|  | Cell-type-specific eQTL | CD4+ Naive T | IL7R | 0.000265716 | 0.08816 | 0.02374 | 37558883 |
|  | Cell-type-specific eQTL | CD4+ Naive T | IL7R | 0.000265716 | 0.08816 | 0.02374 | 37558883 |
|  | Cell-type-specific eQTL | Monocyte (Mono) | IL7R | 3.30E-07 | 0.43874 | 0.08289 | 37558883 |
|  | Cell-type-specific eQTL | Monocyte (Mono) | IL7R | 3.30E-07 | 0.43874 | 0.08289 | 37558883 |
|  | Cell-type-specific eQTL | Monocyte (Mono) | IL7R | 1.09E-07 | 0.7482 | 0.13549 | 37558883 |
|  | Cell-type-specific eQTL | Monocyte (Mono) | IL7R | 1.09E-07 | 0.7482 | 0.13549 | 37558883 |
|  | Cell-type-specific eQTL | Monocyte (Mono) | IL7R | 1.86E-11 | 0.24693 | 0.0346 | 37558883 |
|  | Response eQTL | Ependymal-like 1 | CAPSL | 7.99E-07 | 0.13896 | 0.02815 | 33664506 |
|  | Cell-type-specific eQTL | CD4+ T Cell | IL7R | 0.000276794 | 0.09506 | 0.0257 | 37558883 |
|  | Cell-type-specific eQTL | CD4+ T Cell | IL7R | 0.000276794 | 0.09506 | 0.0257 | 37558883 |
|  | Cell-type-specific eQTL | CD4+ T Cell | IL7R | 0.000393162 | 0.08536 | 0.02369 | 37558883 |
|  | Cell-type-specific eQTL | CD4+ T Cell | IL7R | 0.000393162 | 0.08536 | 0.02369 | 37558883 |
|  | Cell-type-specific eQTL | CD8+ T Cell | IL7R | 0.00027189 | 0.08168 | 0.02244 | 35389781 |
| rs10213865 | Cell-type-specific eQTL | Effector CD4+ T Cell | IL7R | 7.75E-05 | 0.08898 | 0.02205 | 37558883 |
|  | Cell-type-specific eQTL | CD14+ Monocyte (CD14 Mono) | IL7R | 2.50E-12 | 0.24081 | 0.03218 | 37558883 |
|  | Cell-type-specific eQTL | Effector CD4+ T Cell | IL7R | 7.75E-05 | 0.08898 | 0.02205 | 37558883 |
|  | Cell-type-specific eQTL | CD4+ Naive T | IL7R | 1.68E-06 | 0.16777 | 0.034 | 37558883 |
|  | Cell-type-specific eQTL | CD16+ Monocyte (CD16 Mono) | IL7R | 1.50E-05 | 0.58714 | 0.13201 | 37558883 |
|  | Cell-type-specific eQTL | CD8+ T Cell | IL7R | 0.000362618 | 0.07902 | 0.02216 | 35389781 |
|  | Response eQTL | Monocyte (Mono) | IL7R | 3.44E-06 | 4.6426 | 0.99998 | 35672358 |
|  | Response eQTL | Ependymal-like 1 | CAPSL | 4.39E-07 | 0.14257 | 0.02822 | 33664506 |
|  | Cell-type-specific eQTL | Serotonergic-like Neurons | LMBRD2 | 0.000424289 | -0.14197 | 0.04028 | 33664506 |
|  | Cell-type-specific eQTL | Ependymal-like 1 | CAPSL | 4.29E-05 | 0.13229 | 0.03233 | 33664506 |
|  | Cell-type-specific eQTL | CD14+ Monocyte (CD14 Mono) | IL7R | 6.14E-09 | 0.86153 | 0.14125 | 37558883 |
|  | Cell-type-specific eQTL | CD14+ Monocyte (CD14 Mono) | IL7R | 6.14E-09 | 0.86153 | 0.14125 | 37558883 |
|  | Cell-type-specific eQTL | CD14+ Monocyte (CD14 Mono) | IL7R | 8.56E-08 | 0.44582 | 0.08 | 37558883 |
|  | Cell-type-specific eQTL | CD14+ Monocyte (CD14 Mono) | IL7R | 8.56E-08 | 0.44582 | 0.08 | 37558883 |
|  | Cell-type-specific eQTL | CD16+ Monocyte (CD16 Mono) | IL7R | 1.50E-05 | 0.58714 | 0.13201 | 37558883 |
|  | Cell-type-specific eQTL | CD4+ Naive T | IL7R | 0.000820404 | 0.10584 | 0.03116 | 37558883 |
|  | Cell-type-specific eQTL | CD4+ Naive T | IL7R | 0.000820404 | 0.10584 | 0.03116 | 37558883 |
|  | Cell-type-specific eQTL | CD4+ Naive T | IL7R | 0.000190854 | 0.08494 | 0.02234 | 37558883 |
|  | Cell-type-specific eQTL | CD4+ Naive T | IL7R | 0.000190854 | 0.08494 | 0.02234 | 37558883 |
|  | Cell-type-specific eQTL | CD8+ Naive T | IL7R | 0.000376879 | 0.13837 | 0.03823 | 37558883 |
|  | Cell-type-specific eQTL | CD8+ Naive T | IL7R | 0.000376879 | 0.13837 | 0.03823 | 37558883 |
|  | Cell-type-specific eQTL | Monocyte (Mono) | IL7R | 1.01E-12 | 0.2485 | 0.03255 | 37558883 |
|  | Cell-type-specific eQTL | Monocyte (Mono) | IL7R | 1.01E-12 | 0.2485 | 0.03255 | 37558883 |
|  | Cell-type-specific eQTL | Monocyte (Mono) | IL7R | 3.05E-09 | 0.77622 | 0.12474 | 37558883 |
|  | Cell-type-specific eQTL | Monocyte (Mono) | IL7R | 3.05E-09 | 0.77622 | 0.12474 | 37558883 |
|  | Cell-type-specific eQTL | Monocyte (Mono) | IL7R | 6.74E-07 | 0.4043 | 0.07863 | 37558883 |
|  | Cell-type-specific eQTL | Monocyte (Mono) | IL7R | 6.74E-07 | 0.4043 | 0.07863 | 37558883 |
|  | Cell-type-specific eQTL | CD4+ T Cell | IL7R | 0.000255659 | 0.08346 | 0.02243 | 37558883 |
|  | Cell-type-specific eQTL | CD4+ T Cell | IL7R | 0.000255659 | 0.08346 | 0.02243 | 37558883 |
|  | Cell-type-specific eQTL | CD4+ T Cell | IL7R | 0.000112418 | 0.09508 | 0.02415 | 37558883 |
|  | Cell-type-specific eQTL | CD4+ T Cell | IL7R | 0.000112418 | 0.09508 | 0.02415 | 37558883 |
|  | Cell-type-specific eQTL | Ependymal-like 1 | CAPSL | 0.00014539 | 0.16561 | 0.0436 | 33664506 |
|  | Cell-type-specific eQTL | CD14+ Monocyte (CD14 Mono) | IL7R | 2.50E-12 | 0.24081 | 0.03218 | 37558883 |
|  | Cell-type-specific eQTL | CD4+ Naive T | IL7R | 1.68E-06 | 0.16777 | 0.034 | 37558883 |
| rs77973332 | None | None | None | None | None | None | None |
| rs57943165 | None | None | None | None | None | None | None |
| rs1250564 | Cell-type-specific eQTL | CD4+ Central Memory T | ZMIZ1 | 0.000632208 | 0.446 | 0.12555 | 35618845 |
|  | Cell-type-specific eQTL | Natural Killer Cell (NK) | ZMIZ1 | 2.71E-05 | 0.33218 | 0.07722 | 37558883 |
|  | Cell-type-specific eQTL | Naive T Cell | ZMIZ1 | 0.000514387 | 0.43288 | 0.11909 | 35618845 |
|  | Cell-type-specific eQTL | Natural Killer Cell (NK) | PPIF | 0.000179923 | 0.29012 | 0.07596 | 37558883 |
|  | Cell-type-specific eQTL | Natural Killer Cell (NK) | PPIF | 0.000179923 | 0.29012 | 0.07596 | 37558883 |
|  | Cell-type-specific eQTL | Natural Killer Cell (NK) | ZMIZ1 | 2.71E-05 | 0.33218 | 0.07722 | 37558883 |
| rs7895695 | Response eQTL | Serotonergic-like Neurons | RRP12 | 0.000115426 | 0.29939 | 0.07765 | 33664506 |
|  | Cell-type-specific eQTL | Serotonergic-like Neurons | FRAT2 | 0.000714252 | -0.14929 | 0.04412 | 33664506 |
|  | Response eQTL | Monocyte (Mono) | RRP12 | 0.0001348 | 3.81752 | 0.99994 | 35672358 |
|  | Cell-type-specific eQTL | CD14+ Monocyte (CD14 Mono) | RRP12 | 1.86E-07 | 0.28355 | 0.05242 | 37558883 |
|  | Response eQTL | Monocyte (Mono) | FRAT2 | 0.0002839 | 3.62956 | 0.99999 | 35672358 |
|  | Cell-type-specific eQTL | Monocyte (Mono) | RRP12 | 0.00102794 | 0.14643 | 0.04391 | 37558883 |
|  | Cell-type-specific eQTL | CD14+ Monocyte (CD14 Mono) | FRAT2 | 5.52E-09 | 0.16789 | 0.02879 | 35389781 |
|  | Cell-type-specific eQTL | Monocyte (Mono) | FRAT2 | 4.64E-05 | 0.64 | 0.15713 | 35213211 |
|  | Cell-type-specific eQTL | CD16+ Monocyte (CD16 Mono) | RRP12 | 0.000271674 | 0.13722 | 0.03769 | 35389781 |
|  | Cell-type-specific eQTL | CD14+ Monocyte (CD14 Mono) | RRP12 | 3.98E-20 | 0.2979 | 0.03242 | 35389781 |
|  | Cell-type-specific eQTL | CD4+ T Cell | FRAT1 | 2.80E-05 | -0.67 | 0.15994 | 35213211 |
|  | Cell-type-specific eQTL | Monocyte (Mono) | RRP12 | 0.00102794 | 0.14643 | 0.04391 | 37558883 |
|  | Cell-type-specific eQTL | Monocyte (Mono) | RRP12 | 5.72E-07 | 0.24698 | 0.04774 | 37558883 |
|  | Cell-type-specific eQTL | Monocyte (Mono) | RRP12 | 5.72E-07 | 0.24698 | 0.04774 | 37558883 |
|  | Cell-type-specific eQTL | CD14+ Monocyte (CD14 Mono) | RRP12 | 1.86E-07 | 0.28355 | 0.05242 | 37558883 |
| rs694739 | Response eQTL | Natural Killer Cell (NK) | CCDC88B | 2.39E-07 | -5.166 | 1.00001 | 35672358 |
|  | Response eQTL | Natural Killer Cell (NK) | PPP1R14B | 1.95E-09 | -6.002 | 1.00001 | 35672358 |
|  | Response eQTL | CD8+ T Cell | AP003774.1 | 2.47E-06 | 4.7108 | 0.99999 | 35672358 |
|  | Response eQTL | CD8+ T Cell | PPP1R14B | 1.88E-12 | -7.0432 | 1 | 35672358 |
|  | Response eQTL | CD4+ T Cell | PPP1R14B | 1.23E-06 | -4.8508 | 1.00002 | 35672358 |
|  | Response eQTL | CD4+ T Cell | CCDC88B | 3.82E-07 | -5.0778 | 1.00004 | 35672358 |
|  | Response eQTL | CD4+ T Cell | AP003774.1 | 2.79E-07 | 5.1372 | 0.99999 | 35672358 |
|  | Response eQTL | Natural Killer Cell (NK) | AP003774.1 | 5.18E-08 | 5.445 | 0.99998 | 35672358 |
|  | Cell-type-specific eQTL | Natural Killer Cell (NK) | AP003774.1 | 7.59E-07 | 0.56676 | 0.11086 | 37558883 |
|  | Cell-type-specific eQTL | Memory B Cell | AP003774.1 | 0.00027245 | 0.39118 | 0.10552 | 37558883 |
|  | Cell-type-specific eQTL | Naive B Cell | AP003774.1 | 1.06E-05 | 0.64554 | 0.14297 | 37558883 |
|  | Cell-type-specific eQTL | T Cell | AP003774.1 | 0.000115619 | 0.46925 | 0.11524 | 35618845 |
|  | Cell-type-specific eQTL | Naive T Cell | AP003774.1 | 2.39E-06 | 0.54653 | 0.10674 | 35618845 |
|  | Cell-type-specific eQTL | Naive T Cell | AP003774.1 | 3.17E-07 | 0.63637 | 0.1132 | 35618845 |
|  | Cell-type-specific eQTL | Effector CD4+ T Cell | AP003774.1 | 3.52E-08 | 0.57941 | 0.10103 | 37558883 |
|  | Cell-type-specific eQTL | Effector CD4+ T Cell | AP003774.1 | 3.52E-08 | 0.57941 | 0.10103 | 37558883 |
|  | Cell-type-specific eQTL | CD4+ Naive T | AP003774.1 | 3.39E-10 | 0.56771 | 0.08594 | 37558883 |
|  | Cell-type-specific eQTL | CD4+ Naive T | AP003774.1 | 3.39E-10 | 0.56771 | 0.08594 | 37558883 |
|  | Cell-type-specific eQTL | Effector CD4+ T Cell | AP003774.1 | 4.98E-11 | 0.70949 | 0.10199 | 37558883 |
|  | Cell-type-specific eQTL | CD8+ T Cell | AP003774.1 | 2.62E-05 | 0.71703 | 0.16665 | 37558883 |
|  | Cell-type-specific eQTL | CD8+ Memory | AP003774.1 | 3.96E-07 | 0.77082 | 0.14699 | 37558883 |
|  | Cell-type-specific eQTL | CD8+ Memory | AP003774.1 | 3.96E-07 | 0.77082 | 0.14699 | 37558883 |
|  | Cell-type-specific eQTL | CD8+ Memory | AP003774.1 | 1.56E-06 | 0.69805 | 0.14085 | 37558883 |
|  | Cell-type-specific eQTL | CD8+ Memory | AP003774.1 | 1.56E-06 | 0.69805 | 0.14085 | 37558883 |
|  | Cell-type-specific eQTL | CD8+ Memory | PPP1R14B | 0.000101195 | -0.22263 | 0.05608 | 37558883 |
|  | Cell-type-specific eQTL | CD8+ Memory | PPP1R14B | 0.000101195 | -0.22263 | 0.05608 | 37558883 |
|  | Cell-type-specific eQTL | CD8+ Memory | AP003774.1 | 1.88E-06 | 0.65029 | 0.13236 | 37558883 |
|  | Cell-type-specific eQTL | CD8+ Naive T | AP003774.1 | 4.06E-07 | 0.62409 | 0.11895 | 37558883 |
|  | Cell-type-specific eQTL | CD8+ Naive T | PPP1R14B | 0.000219813 | -0.19592 | 0.05202 | 37558883 |
|  | Cell-type-specific eQTL | CD8+ Naive T | PPP1R14B | 0.000219813 | -0.19592 | 0.05202 | 37558883 |
|  | Cell-type-specific eQTL | CD8+ Naive T | AP003774.1 | 9.42E-10 | 0.71061 | 0.11027 | 37558883 |
|  | Cell-type-specific eQTL | CD8+ Naive T | AP003774.1 | 9.42E-10 | 0.71061 | 0.11027 | 37558883 |
|  | Cell-type-specific eQTL | CD8+ Naive T | AP003774.1 | 1.85E-05 | 0.49516 | 0.11277 | 37558883 |
|  | Cell-type-specific eQTL | CD8+ Naive T | AP003774.1 | 1.85E-05 | 0.49516 | 0.11277 | 37558883 |
|  | Cell-type-specific eQTL | CD8+ T Cell | AP003774.1 | 2.62E-05 | 0.71703 | 0.16665 | 37558883 |
|  | Cell-type-specific eQTL | Naive T Cell | AP003774.1 | 3.28E-06 | 0.57164 | 0.11344 | 35618845 |
|  | Cell-type-specific eQTL | Naive T Cell | AP003774.1 | 1.40E-08 | 0.70765 | 0.11101 | 35618845 |
|  | Cell-type-specific eQTL | Naive T Cell | AP003774.1 | 1.95E-12 | 0.69841 | 0.0828 | 35618845 |
|  | Cell-type-specific eQTL | Naive T Cell | CCDC88B | 0.000358123 | -0.2519 | 0.06727 | 35618845 |
|  | Cell-type-specific eQTL | Naive T Cell | AP003774.1 | 1.38E-14 | 0.94667 | 0.09856 | 35618845 |
|  | Cell-type-specific eQTL | CD4+ T Cell | AP003774.1 | 2.89E-16 | 1.04 | 0.12718 | 35213211 |
|  | Cell-type-specific eQTL | CD4+ T Cell | AP003774.1 | 1.03E-15 | 1.01 | 0.12588 | 35213211 |
|  | Cell-type-specific eQTL | CD4+ T Cell | AP003774.1 | 1.06E-16 | 1.02 | 0.12293 | 35213211 |
|  | Cell-type-specific eQTL | B Cell | AP003774.1 | 4.12E-10 | 0.72982 | 0.11089 | 37558883 |
|  | Cell-type-specific eQTL | B Cell | AP003774.1 | 4.12E-10 | 0.72982 | 0.11089 | 37558883 |
|  | Cell-type-specific eQTL | B Cell | AP003774.1 | 2.64E-07 | 0.51871 | 0.09725 | 37558883 |
|  | Cell-type-specific eQTL | B Cell | AP003774.1 | 2.64E-07 | 0.51871 | 0.09725 | 37558883 |
|  | Cell-type-specific eQTL | CD8+ Naive T | AP003774.1 | 4.06E-07 | 0.62409 | 0.11895 | 37558883 |
|  | Cell-type-specific eQTL | CD4+ T Cell | GPR137 | 0.000165899 | -0.16091 | 0.04192 | 37558883 |
|  | Cell-type-specific eQTL | CD4+ T Cell | AP003774.1 | 5.30E-22 | 0.69566 | 0.06412 | 37558883 |
|  | Cell-type-specific eQTL | CD4+ T Cell | AP003774.1 | 5.30E-22 | 0.69566 | 0.06412 | 37558883 |
|  | Cell-type-specific eQTL | CD4+ T Cell | AP003774.1 | 4.99E-14 | 0.54654 | 0.06753 | 37558883 |
|  | Cell-type-specific eQTL | CD4+ T Cell | AP003774.1 | 4.99E-14 | 0.54654 | 0.06753 | 37558883 |
|  | Cell-type-specific eQTL | Natural Killer Cell (NK) | AP003774.1 | 2.47E-10 | 0.84926 | 0.127 | 37558883 |
|  | Cell-type-specific eQTL | Natural Killer Cell (NK) | AP003774.1 | 2.47E-10 | 0.84926 | 0.127 | 37558883 |
|  | Cell-type-specific eQTL | Natural Killer Cell (NK) | AP003774.1 | 7.59E-07 | 0.56676 | 0.11086 | 37558883 |
|  | Cell-type-specific eQTL | CD8+ T Cell | PPP1R14B | 2.96E-05 | -0.18051 | 0.04222 | 37558883 |
|  | Cell-type-specific eQTL | CD8+ T Cell | AP003774.1 | 2.81E-12 | 0.59305 | 0.07959 | 37558883 |
|  | Cell-type-specific eQTL | CD8+ T Cell | AP003774.1 | 2.81E-12 | 0.59305 | 0.07959 | 37558883 |
|  | Cell-type-specific eQTL | CD8+ T Cell | PPP1R14B | 6.34E-07 | -0.24183 | 0.04698 | 37558883 |
|  | Cell-type-specific eQTL | CD8+ T Cell | PPP1R14B | 6.34E-07 | -0.24183 | 0.04698 | 37558883 |
|  | Cell-type-specific eQTL | CD4+ T Cell | AP003774.1 | 1.04E-17 | 0.68028 | 0.07215 | 37558883 |
|  | Cell-type-specific eQTL | CD4+ T Cell | AP003774.1 | 1.04E-17 | 0.68028 | 0.07215 | 37558883 |
|  | Cell-type-specific eQTL | CD4+ T Cell | GPR137 | 0.000165899 | -0.16091 | 0.04192 | 37558883 |
|  | Cell-type-specific eQTL | Excitatory Neurons | GPR137 | 9.78E-05 | -0.13769 | 0.03534 | 35915177 |
|  | Cell-type-specific eQTL | Excitatory Neurons | PLCB3 | 4.70E-06 | -0.26853 | 0.05866 | 35915177 |
|  | Cell-type-specific eQTL | Astrocytes | CCDC88B | 1.77E-10 | -0.35717 | 0.05598 | 35915177 |
|  | Cell-type-specific eQTL | CD8+ T Cell | AP003774.1 | 1.52E-16 | 0.68057 | 0.07539 | 37558883 |
|  | Cell-type-specific eQTL | CD8+ T Cell | AP003774.1 | 1.52E-16 | 0.68057 | 0.07539 | 37558883 |
|  | Cell-type-specific eQTL | CD8+ T Cell | AP003774.1 | 1.88E-14 | 0.6279 | 0.07592 | 37558883 |
|  | Cell-type-specific eQTL | CD8+ T Cell | AP003774.1 | 1.88E-14 | 0.6279 | 0.07592 | 37558883 |
|  | Cell-type-specific eQTL | CD8+ T Cell | PPP1R14B | 2.96E-05 | -0.18051 | 0.04222 | 37558883 |
|  | Cell-type-specific eQTL | Effector CD4+ T Cell | AP003774.1 | 4.98E-11 | 0.70949 | 0.10199 | 37558883 |
|  | Cell-type-specific eQTL | Effector CD4+ T Cell | AP003774.1 | 3.98E-17 | 0.826 | 0.0895 | 37558883 |
|  | Cell-type-specific eQTL | Effector CD4+ T Cell | AP003774.1 | 3.98E-17 | 0.826 | 0.0895 | 37558883 |
|  | Cell-type-specific eQTL | Natural Killer Cell (NK) | AP003774.1 | 1.55E-16 | 1.03 | 0.1248 | 35213211 |
|  | Cell-type-specific eQTL | Monocyte (Mono) | AP003774.1 | 2.90E-12 | 0.9 | 0.12889 | 35213211 |
|  | Cell-type-specific eQTL | Monocyte (Mono) | AP003774.1 | 2.24E-12 | 0.88 | 0.12538 | 35213211 |
|  | Cell-type-specific eQTL | CD8+ Naive T | AP003774.1 | 3.03E-06 | 0.68 | 0.14564 | 35213211 |
|  | Cell-type-specific eQTL | CD8+ Naive T | AP003774.1 | 6.94E-17 | 1.03 | 0.12338 | 35213211 |
|  | Cell-type-specific eQTL | CD4 Memory | AP003774.1 | 7.03E-15 | 0.8334 | 0.08537 | 35618845 |
|  | Cell-type-specific eQTL | CD4 Memory | CCDC88B | 1.56E-06 | -0.25653 | 0.049 | 35618845 |
|  | Cell-type-specific eQTL | CD4 Memory | AP003774.1 | 2.23E-14 | 0.87181 | 0.09154 | 35618845 |
|  | Cell-type-specific eQTL | CD4 Memory | AP003774.1 | 1.31E-06 | 0.66344 | 0.12651 | 35618845 |
|  | Cell-type-specific eQTL | Naive Regulatory T | AP003774.1 | 2.00E-10 | 0.89 | 0.13991 | 35213211 |
|  | Cell-type-specific eQTL | Memory Regulatory T | AP003774.1 | 2.84E-15 | 0.99 | 0.12535 | 35213211 |
|  | Cell-type-specific eQTL | CD4+ T Cell | AP003774.1 | 1.05E-13 | 0.98 | 0.13181 | 35213211 |
|  | Cell-type-specific eQTL | CD4+ T Cell | AP003774.1 | 2.72E-17 | 1.06 | 0.12532 | 35213211 |
|  | Cell-type-specific eQTL | CD4+ Central Memory T | AP003774.1 | 3.77E-08 | 0.80695 | 0.13308 | 35618845 |
|  | Cell-type-specific eQTL | CD4+ Naive T | AP003774.1 | 1.08E-06 | 0.76333 | 0.14481 | 35618845 |
|  | Cell-type-specific eQTL | CD4+ Naive T | AP003774.1 | 9.88E-13 | 0.92714 | 0.10606 | 35618845 |
|  | Cell-type-specific eQTL | CD4+ Naive T | AP003774.1 | 4.89E-11 | 0.94139 | 0.12179 | 35618845 |
|  | Cell-type-specific eQTL | CD4+ Naive T | AP003774.1 | 0.000246885 | 0.47337 | 0.12349 | 35618845 |
|  | Cell-type-specific eQTL | CD4 Memory | AP003774.1 | 1.89E-10 | 1.04637 | 0.14412 | 35618845 |
|  | Cell-type-specific eQTL | CD4 Memory | RP11-783K16.5 | 8.21E-05 | -0.59153 | 0.14278 | 35618845 |
|  | Cell-type-specific eQTL | CD4 Memory | PPP1R14B | 0.000108959 | -0.57361 | 0.14114 | 35618845 |
|  | Cell-type-specific eQTL | Naive T Cell | RP11-783K16.5 | 0.000927337 | -0.44875 | 0.12997 | 35618845 |
|  | Cell-type-specific eQTL | Naive T Cell | AP003774.1 | 2.36E-05 | 0.69137 | 0.1543 | 35618845 |
|  | Cell-type-specific eQTL | Naive T Cell | AP003774.1 | 1.12E-08 | 0.92107 | 0.14517 | 35618845 |
|  | Cell-type-specific eQTL | CD4+ Effector Memory T | AP003774.1 | 6.69E-13 | 0.88336 | 0.10177 | 35618845 |
|  | Cell-type-specific eQTL | CD4+ Effector Memory T | AP003774.1 | 1.14E-06 | 0.56631 | 0.10665 | 35618845 |
|  | Cell-type-specific eQTL | CD4+ Central Memory T | AP003774.1 | 4.58E-12 | 0.71979 | 0.08737 | 35618845 |
|  | Cell-type-specific eQTL | CD4+ Central Memory T | AP003774.1 | 3.03E-08 | 0.66078 | 0.10638 | 35618845 |
|  | Cell-type-specific eQTL | CD4+ Central Memory T | AP003774.1 | 0.00093386 | 0.47134 | 0.1373 | 35618845 |
|  | Response eQTL | B Cell | PPP1R14B | 5.86E-06 | -4.5312 | 1.00001 | 35672358 |
|  | Response eQTL | Natural Killer Cell (NK) | CCDC88B | 3.98E-05 | -4.1086 | 1.00003 | 35672358 |
|  | Response eQTL | Natural Killer Cell (NK) | PPP1R14B | 1.43E-08 | -5.67 | 1 | 35672358 |
|  | Response eQTL | CD8+ T Cell | AP003774.1 | 1.86E-07 | 5.2132 | 0.99998 | 35672358 |
|  | Response eQTL | CD8+ T Cell | PPP1R14B | 3.66E-11 | -6.6174 | 1.00002 | 35672358 |
|  | Response eQTL | CD4+ T Cell | AP003774.1 | 2.24E-05 | 4.2394 | 0.99998 | 35672358 |
|  | Response eQTL | CD4+ T Cell | PPP1R14B | 3.63E-08 | -5.5078 | 1.00002 | 35672358 |
|  | Response eQTL | Natural Killer Cell (NK) | AP003774.1 | 0.0002758 | 3.63703 | 1 | 35672358 |
|  | Cell-type-specific eQTL | B Cell | AP003774.1 | 1.52E-06 | 4.8082 | 0.99997 | 35672358 |
|  | Response eQTL | Natural Killer Cell (NK) | CCDC88B | 2.91E-05 | -4.1806 | 1.00003 | 35672358 |
|  | Response eQTL | Natural Killer Cell (NK) | AP003774.1 | 1.56E-05 | 4.3196 | 0.99996 | 35672358 |
|  | Response eQTL | Natural Killer Cell (NK) | PPP1R14B | 3.93E-11 | -6.6066 | 1.00003 | 35672358 |
|  | Response eQTL | CD8+ T Cell | AP003774.1 | 4.45E-07 | 5.0488 | 0.99998 | 35672358 |
|  | Response eQTL | CD8+ T Cell | PPP1R14B | 5.72E-11 | -6.5508 | 1.00002 | 35672358 |
|  | Response eQTL | CD4+ T Cell | PPP1R14B | 1.61E-06 | -4.7976 | 1.00001 | 35672358 |
|  | Response eQTL | CD4+ T Cell | AP003774.1 | 4.97E-13 | 7.2262 | 0.99999 | 35672358 |
|  | Cell-type-specific eQTL | B Cell | PPP1R14B | 0.000157695 | -0.65914 | 0.17444 | 34822289 |
|  | Cell-type-specific eQTL | Natural Killer Cell (NK) | TRMT112 | 3.02E-07 | 0.09307 | 0.01817 | 35389781 |
|  | Cell-type-specific eQTL | CD14+ Monocyte (CD14 Mono) | CCDC88B | 0.000657881 | -0.07088 | 0.02081 | 35389781 |
|  | Cell-type-specific eQTL | Natural Killer Cell (NK) | CCDC88B | 0.0001225 | -3.84107 | 1.00004 | 35672358 |
|  | Cell-type-specific eQTL | Natural Killer Cell (NK) | AP003774.1 | 5.95E-05 | 4.0148 | 0.99998 | 35672358 |
|  | Cell-type-specific eQTL | CD8+ T Cell | AP003774.1 | 7.92E-05 | 3.9468 | 0.99997 | 35672358 |
|  | Cell-type-specific eQTL | CD4+ T Cell | CCDC88B | 8.85E-06 | -4.4434 | 1.00002 | 35672358 |
|  | Cell-type-specific eQTL | CD4+ T Cell | AP003774.1 | 2.62E-09 | 5.9536 | 0.99999 | 35672358 |
|  | Cell-type-specific eQTL | CD4+ Naive T | AP003774.1 | 5.51E-06 | 0.66 | 0.14523 | 35213211 |
|  | Cell-type-specific eQTL | CD4+ Naive T | AP003774.1 | 2.08E-13 | 0.97 | 0.13209 | 35213211 |
|  | Cell-type-specific eQTL | Naive B Cell | AP003774.1 | 8.84E-16 | 0.99 | 0.1231 | 35213211 |
|  | Cell-type-specific eQTL | CD8+ T Cell | PPP1R14B | 3.79E-05 | -0.71354 | 0.17318 | 34822289 |
|  | Cell-type-specific eQTL | CD4+ T Cell | PPP1R14B | 0.000458026 | -0.66225 | 0.18899 | 34822289 |
|  | Cell-type-specific eQTL | CD4+ T Cell | AP003774.4 | 0.000413503 | 0.69744 | 0.1975 | 34822289 |
|  | Cell-type-specific eQTL | CD4+ T Cell | AP003774.4 | 0.000244416 | 0.64888 | 0.1769 | 34822289 |
|  | Cell-type-specific eQTL | CD4+ T Cell | PPP1R14B | 7.47E-08 | -0.97415 | 0.18109 | 34822289 |
|  | Response eQTL | B Cell | CCDC88B | 6.08E-05 | -4.0096 | 1.00002 | 35672358 |
|  | Cell-type-specific eQTL | Ependymal-like 1 | FKBP2 | 0.000502648 | 0.17801 | 0.05116 | 33664506 |
|  | Response eQTL | Ependymal-like 1 | PRDX5 | 7.38E-08 | 0.32918 | 0.06117 | 33664506 |
|  | Cell-type-specific eQTL | Dopaminergic Neurons | PLCB3 | 3.83E-05 | -0.23225 | 0.0564 | 33664506 |
|  | Cell-type-specific eQTL | Floor Plate Progenitors | TRPT1 | 9.73E-05 | -0.18657 | 0.04787 | 33664506 |
|  | Cell-type-specific eQTL | Oligodendrocytes | RPS6KA4 | 7.09E-06 | -0.28224 | 0.06285 | 35915177 |
|  | Cell-type-specific eQTL | Microglia (Micro) | RPS6KA4 | 3.60E-05 | -0.28246 | 0.06836 | 35915177 |
|  | Cell-type-specific eQTL | Excitatory Neurons | CCDC88B | 1.18E-19 | -0.54766 | 0.06038 | 35915177 |
|  | Response eQTL | B Cell | AP003774.1 | 6.81E-05 | 3.983 | 0.99999 | 35672358 |
|  | Response eQTL | Natural Killer Cell (NK) | PPP1R14B | 1.03E-05 | -4.4118 | 1.00004 | 35672358 |
|  | Response eQTL | Natural Killer Cell (NK) | AP003774.1 | 2.84E-14 | 7.6054 | 0.99998 | 35672358 |
|  | Response eQTL | CD8+ T Cell | RP11-783K16.5 | 1.82E-05 | -4.2854 | 1.00004 | 35672358 |
|  | Response eQTL | CD8+ T Cell | PPP1R14B | 1.42E-05 | -4.3412 | 1 | 35672358 |
|  | Response eQTL | CD8+ T Cell | AP003774.1 | 1.13E-07 | 5.3044 | 0.99999 | 35672358 |
|  | Response eQTL | CD4+ T Cell | PPP1R14B | 8.10E-07 | -4.933 | 1.00003 | 35672358 |
|  | Response eQTL | CD4+ T Cell | AP003774.1 | 1.71E-13 | 7.3698 | 0.99999 | 35672358 |
|  | Response eQTL | Natural Killer Cell (NK) | PPP1R14B | 8.33E-09 | -5.7616 | 1 | 35672358 |
|  | Response eQTL | CD8+ T Cell | PPP1R14B | 0.0002158 | -3.69976 | 1.00003 | 35672358 |
|  | Response eQTL | CD8+ T Cell | AP003774.1 | 2.19E-09 | 5.9832 | 0.99998 | 35672358 |
|  | Response eQTL | CD4+ T Cell | AP003774.1 | 1.85E-07 | 5.2136 | 0.99998 | 35672358 |
|  | Response eQTL | Natural Killer Cell (NK) | PPP1R14B | 1.37E-06 | -4.8298 | 1.00001 | 35672358 |
|  | Response eQTL | CD8+ T Cell | PPP1R14B | 3.26E-05 | -4.1546 | 1.00005 | 35672358 |
|  | Response eQTL | CD8+ T Cell | AP003774.1 | 7.43E-12 | 6.8492 | 0.99999 | 35672358 |
|  | Response eQTL | CD4+ T Cell | AP003774.1 | 1.95E-07 | 5.2036 | 0.99999 | 35672358 |
|  | Cell-type-specific eQTL | Natural Killer Cell (NK) | AP003774.1 | 1.92E-05 | 0.76483 | 0.17475 | 37558883 |
|  | Cell-type-specific eQTL | Natural Killer Cell (NK) | AP003774.1 | 1.92E-05 | 0.76483 | 0.17475 | 37558883 |
|  | Cell-type-specific eQTL | Natural Killer Cell (NK) | AP003774.1 | 1.25E-06 | 0.77028 | 0.15388 | 37558883 |
|  | Cell-type-specific eQTL | CD8+ Memory | AP003774.1 | 1.88E-06 | 0.65029 | 0.13236 | 37558883 |
|  | Cell-type-specific eQTL | CD4+ Naive T | AP003774.1 | 4.64E-13 | 0.75229 | 0.09701 | 37558883 |
|  | Cell-type-specific eQTL | CD4+ Naive T | AP003774.1 | 4.64E-13 | 0.75229 | 0.09701 | 37558883 |
|  | Cell-type-specific eQTL | CD4+ Naive T | AP003774.1 | 1.14E-09 | 0.54719 | 0.08569 | 37558883 |
|  | Cell-type-specific eQTL | CD4+ Naive T | AP003774.1 | 1.14E-09 | 0.54719 | 0.08569 | 37558883 |
|  | Cell-type-specific eQTL | Natural Killer Cell (NK) | AP003774.1 | 2.19E-05 | 0.5625 | 0.12932 | 37558883 |
|  | Cell-type-specific eQTL | Natural Killer Cell (NK) | AP003774.1 | 2.19E-05 | 0.5625 | 0.12932 | 37558883 |
|  | Cell-type-specific eQTL | Naive B Cell | AP003774.1 | 1.06E-05 | 0.64554 | 0.14297 | 37558883 |
|  | Cell-type-specific eQTL | Natural Killer Cell (NK) | AP003774.1 | 1.25E-06 | 0.77028 | 0.15388 | 37558883 |
|  | Cell-type-specific eQTL | Mucosal invariant T cell | AP003774.1 | 2.40E-08 | 0.90728 | 0.15594 | 37558883 |
|  | Cell-type-specific eQTL | Mucosal invariant T cell | AP003774.1 | 2.40E-08 | 0.90728 | 0.15594 | 37558883 |
|  | Cell-type-specific eQTL | Memory B Cell | AP003774.1 | 0.00027245 | 0.39118 | 0.10552 | 37558883 |
| rs12826560 | Cell-type-specific eQTL | Memory B Cell | KLRK1 | 4.01E-05 | 0.59742 | 0.14207 | 37558883 |
|  | Cell-type-specific eQTL | B Cell | KLRK1 | 1.10E-11 | 0.99888 | 0.13829 | 37558883 |
|  | Cell-type-specific eQTL | B Cell | KLRK1 | 1.10E-11 | 0.99888 | 0.13829 | 37558883 |
|  | Cell-type-specific eQTL | B Cell | RP11-277P12.20 | 1.92E-06 | 0.50858 | 0.10359 | 37558883 |
|  | Response eQTL | B Cell | RP11-277P12.20 | 2.71E-06 | 0.54852 | 0.11344 | 37558883 |
|  | Cell-type-specific eQTL | Oligodendrocyte Precursor Cell (OPC) | KLRC4-KLRK1 | 1.18E-08 | 0.73213 | 0.12838 | 35915177 |
|  | Cell-type-specific eQTL | Oligodendrocyte Precursor Cell (OPC) | RP11-277P12.6 | 2.42E-08 | 0.554 | 0.0993 | 35915177 |
|  | Cell-type-specific eQTL | Naive B Cell | KLRK1 | 2.88E-09 | 1.26716 | 0.20392 | 37558883 |
|  | Cell-type-specific eQTL | Naive B Cell | KLRK1 | 9.21E-06 | 0.88758 | 0.19488 | 37558883 |
|  | Cell-type-specific eQTL | Naive B Cell | KLRK1 | 9.21E-06 | 0.88758 | 0.19488 | 37558883 |
|  | Cell-type-specific eQTL | Naive B Cell | KLRK1 | 1.61E-05 | 0.91729 | 0.20759 | 37558883 |
|  | Cell-type-specific eQTL | Naive B Cell | RP11-277P12.20 | 5.88E-06 | 0.60701 | 0.13046 | 37558883 |
|  | Cell-type-specific eQTL | Naive B Cell | RP11-277P12.20 | 5.88E-06 | 0.60701 | 0.13046 | 37558883 |
|  | Cell-type-specific eQTL | Memory B Cell | KLRK1 | 6.24E-06 | 0.67665 | 0.14571 | 37558883 |
|  | Cell-type-specific eQTL | Memory B Cell | KLRK1 | 6.24E-06 | 0.67665 | 0.14571 | 37558883 |
|  | Cell-type-specific eQTL | CD8+ Naive T | KLRK1 | 0.000333271 | 0.11341 | 0.03104 | 37558883 |
|  | Cell-type-specific eQTL | Memory B Cell | KLRK1 | 4.01E-05 | 0.59742 | 0.14207 | 37558883 |
|  | Cell-type-specific eQTL | Naive B Cell | KLRK1 | 2.88E-09 | 1.26716 | 0.20392 | 37558883 |
|  | Cell-type-specific eQTL | Natural Killer Cell (NK) | KLRC1 | 4.97E-06 | 0.36509 | 0.07778 | 37558883 |
|  | Cell-type-specific eQTL | Natural Killer Cell (NK) | KLRC2 | 1.21E-11 | 0.88673 | 0.12319 | 37558883 |
|  | Cell-type-specific eQTL | Natural Killer Cell (NK) | KLRC2 | 1.21E-11 | 0.88673 | 0.12319 | 37558883 |
|  | Cell-type-specific eQTL | Naive B Cell | RP11-277P12.20 | 1.41E-05 | 0.71131 | 0.15969 | 37558883 |
|  | Cell-type-specific eQTL | Naive B Cell | RP11-277P12.20 | 1.41E-05 | 0.71131 | 0.15969 | 37558883 |
|  | Cell-type-specific eQTL | Naive B Cell | KLRK1 | 2.95E-06 | 0.93464 | 0.19431 | 37558883 |
|  | Cell-type-specific eQTL | Naive B Cell | KLRK1 | 2.95E-06 | 0.93464 | 0.19431 | 37558883 |
|  | Cell-type-specific eQTL | Naive B Cell | KLRK1 | 1.61E-05 | 0.91729 | 0.20759 | 37558883 |
|  | Cell-type-specific eQTL | Natural Killer Cell (NK) | KLRC1 | 6.22E-06 | 0.60094 | 0.12949 | 37558883 |
|  | Cell-type-specific eQTL | Natural Killer Cell (NK) | KLRC1 | 6.22E-06 | 0.60094 | 0.12949 | 37558883 |
|  | Cell-type-specific eQTL | Natural Killer Cell (NK) | KLRC3 | 2.66E-06 | 0.88032 | 0.18218 | 37558883 |
|  | Cell-type-specific eQTL | Natural Killer Cell (NK) | KLRC3 | 2.66E-06 | 0.88032 | 0.18218 | 37558883 |
|  | Cell-type-specific eQTL | CD8+ Naive T | KLRC4 | 2.73E-08 | -0.82598 | 0.14251 | 37558883 |
|  | Cell-type-specific eQTL | CD8+ Naive T | RP11-277P12.9 | 6.93E-07 | -0.78 | 0.15715 | 35213211 |
|  | Cell-type-specific eQTL | Natural Killer Cell (NK) | KLRC2 | 1.45E-07 | 0.56475 | 0.1035 | 37558883 |
|  | Cell-type-specific eQTL | Natural Killer Cell (NK) | KLRC1 | 8.50E-06 | 0.39916 | 0.08729 | 37558883 |
|  | Cell-type-specific eQTL | Natural Killer Cell (NK) | KLRC1 | 8.50E-06 | 0.39916 | 0.08729 | 37558883 |
|  | Cell-type-specific eQTL | Natural Killer Cell (NK) | KLRC3 | 7.73E-14 | 0.81238 | 0.10073 | 37558883 |
|  | Cell-type-specific eQTL | CD8+ Memory | KLRC4 | 4.20E-06 | -0.59121 | 0.12491 | 37558883 |
|  | Cell-type-specific eQTL | CD8+ Memory | KLRC4 | 4.20E-06 | -0.59121 | 0.12491 | 37558883 |
|  | Cell-type-specific eQTL | Natural Killer Cell (NK) | KLRC3 | 4.61E-12 | 0.913 | 0.12401 | 37558883 |
|  | Cell-type-specific eQTL | Natural Killer Cell (NK) | KLRC3 | 4.61E-12 | 0.913 | 0.12401 | 37558883 |
|  | Cell-type-specific eQTL | Natural Killer Cell (NK) | KLRC1 | 7.91E-10 | 0.65121 | 0.10074 | 37558883 |
|  | Cell-type-specific eQTL | Natural Killer Cell (NK) | KLRC1 | 7.91E-10 | 0.65121 | 0.10074 | 37558883 |
|  | Cell-type-specific eQTL | Natural Killer Cell (NK) | KLRC2 | 1.03E-08 | 0.88888 | 0.14859 | 37558883 |
|  | Cell-type-specific eQTL | CD8+ Naive T | KLRC4 | 2.36E-06 | -0.6131 | 0.12591 | 37558883 |
|  | Cell-type-specific eQTL | CD8+ Naive T | KLRC4 | 2.73E-08 | -0.82598 | 0.14251 | 37558883 |
|  | Cell-type-specific eQTL | Natural Killer Cell (NK) | KLRC1 | 4.97E-06 | 0.36509 | 0.07778 | 37558883 |
|  | Cell-type-specific eQTL | Natural Killer Cell (NK) | KLRC1 | 6.53E-09 | 0.48602 | 0.08 | 37558883 |
|  | Cell-type-specific eQTL | Natural Killer Cell (NK) | KLRC1 | 6.53E-09 | 0.48602 | 0.08 | 37558883 |
|  | Cell-type-specific eQTL | Natural Killer Cell (NK) | KLRC2 | 6.76E-11 | 0.80082 | 0.11581 | 37558883 |
|  | Cell-type-specific eQTL | Natural Killer Cell (NK) | KLRC2 | 6.76E-11 | 0.80082 | 0.11581 | 37558883 |
|  | Cell-type-specific eQTL | Natural Killer Cell (NK) | KLRC3 | 7.73E-14 | 0.81238 | 0.10073 | 37558883 |
|  | Cell-type-specific eQTL | B Cell | RP11-277P12.20 | 1.92E-06 | 0.50858 | 0.10359 | 37558883 |
|  | Cell-type-specific eQTL | B Cell | KLRK1 | 1.38E-08 | 0.72146 | 0.12177 | 37558883 |
|  | Cell-type-specific eQTL | B Cell | KLRK1 | 1.38E-08 | 0.72146 | 0.12177 | 37558883 |
|  | Cell-type-specific eQTL | CD8+ Memory | KLRC4 | 2.74E-05 | -0.48293 | 0.11248 | 37558883 |
|  | Cell-type-specific eQTL | CD8+ Memory | KLRC4 | 2.74E-05 | -0.48293 | 0.11248 | 37558883 |
|  | Cell-type-specific eQTL | CD8+ Naive T | KLRC4 | 4.16E-06 | -0.62355 | 0.13161 | 37558883 |
|  | Cell-type-specific eQTL | CD8+ Naive T | KLRC4 | 4.16E-06 | -0.62355 | 0.13161 | 37558883 |
|  | Cell-type-specific eQTL | CD8+ Naive T | KLRC4 | 2.36E-06 | -0.6131 | 0.12591 | 37558883 |
|  | Cell-type-specific eQTL | CD8+ Naive T | KLRK1 | 0.000333271 | 0.11341 | 0.03104 | 37558883 |
|  | Cell-type-specific eQTL | Memory Regulatory T | KLRC1 | 5.53E-05 | 0.65 | 0.16121 | 35213211 |
|  | Cell-type-specific eQTL | CD4+ T Cell | KLRC4 | 0.000688402 | -0.18938 | 0.05495 | 37558883 |
|  | Cell-type-specific eQTL | CD4+ T Cell | KLRC4 | 0.000688402 | -0.18938 | 0.05495 | 37558883 |
|  | Cell-type-specific eQTL | CD4+ T Cell | KLRC4 | 2.34E-05 | -0.32819 | 0.07577 | 37558883 |
|  | Cell-type-specific eQTL | CD8+ T Cell | KLRC4 | 0.000300707 | -0.33782 | 0.0918 | 37558883 |
|  | Cell-type-specific eQTL | Naive B Cell | KLRK1 | 8.88E-10 | 0.91 | 0.14849 | 35213211 |
|  | Cell-type-specific eQTL | CD8+ Naive T | RP11-277P12.20 | 9.69E-08 | -0.82 | 0.15378 | 35213211 |
|  | Cell-type-specific eQTL | CD8+ Naive T | KLRC4-KLRK1 | 1.04E-07 | -0.82 | 0.15414 | 35213211 |
|  | Cell-type-specific eQTL | CD8+ T Cell | KLRC4 | 8.09E-07 | -0.43787 | 0.08595 | 37558883 |
|  | Cell-type-specific eQTL | CD8+ T Cell | KLRC4 | 8.09E-07 | -0.43787 | 0.08595 | 37558883 |
|  | Cell-type-specific eQTL | CD8+ T Cell | KLRK1 | 1.96E-05 | 0.11817 | 0.02701 | 37558883 |
|  | Cell-type-specific eQTL | CD8+ T Cell | KLRK1 | 1.96E-05 | 0.11817 | 0.02701 | 37558883 |
|  | Response eQTL | B Cell | RP11-277P12.20 | 2.71E-06 | 0.54852 | 0.11344 | 37558883 |
|  | Cell-type-specific eQTL | CD4+ T Cell | KLRC4 | 2.34E-05 | -0.32819 | 0.07577 | 37558883 |
|  | Cell-type-specific eQTL | Naive Regulatory T | KLRK1 | 3.74E-05 | 0.64 | 0.15524 | 35213211 |
|  | Cell-type-specific eQTL | Natural Killer Cell (NK) | KLRC2 | 3.56E-05 |  |  | 10.1126/science.abf3041 |
|  | Cell-type-specific eQTL | Oligodendrocytes | KLRC4-KLRK1 | 3.08E-10 | 0.7751 | 0.12313 | 35915177 |
|  | Cell-type-specific eQTL | Oligodendrocytes | RP11-277P12.6 | 1.52E-06 | 0.52151 | 0.10844 | 35915177 |
|  | Cell-type-specific eQTL | Natural Killer Cell (NK) | RP11-277P12.20 | 1.56E-06 | 0.75 | 0.15615 | 35213211 |
|  | Cell-type-specific eQTL | Natural Killer Cell (NK) | KLRC4-KLRK1 | 2.79E-06 | 0.72 | 0.15366 | 35213211 |
|  | Cell-type-specific eQTL | Natural Killer Cell (NK) | KLRC1 | 3.96E-06 | 0.72 | 0.15607 | 35213211 |
|  | Cell-type-specific eQTL | CD8+ T Cell | KLRC4 | 1.33E-07 | -0.36799 | 0.06976 | 35389781 |
|  | Cell-type-specific eQTL | Natural Killer Cell (NK) | KLRC2 | 5.39E-09 | 0.60714 | 0.10406 | 35389781 |
|  | Cell-type-specific eQTL | Natural Killer Cell (NK) | KLRC1 | 2.49E-05 | 0.33363 | 0.07915 | 35389781 |
|  | Cell-type-specific eQTL | Natural Killer Cell (NK) | KLRC2 | 1.45E-07 | 0.56475 | 0.1035 | 37558883 |
|  | Cell-type-specific eQTL | CD8+ Naive T | KLRC4-KLRK1 | 2.21E-07 | -0.79 | 0.15249 | 35213211 |
|  | Cell-type-specific eQTL | Natural Killer Cell (NK) | KLRK1 | 2.51E-05 | 0.14246 | 0.03297 | 37558883 |
|  | Cell-type-specific eQTL | Natural Killer Cell (NK) | KLRC2 | 3.25E-06 | 0.52168 | 0.10883 | 37558883 |
|  | Cell-type-specific eQTL | Natural Killer Cell (NK) | KLRC3 | 1.09E-08 | 0.45419 | 0.07604 | 37558883 |
|  | Cell-type-specific eQTL | Natural Killer Cell (NK) | KLRC3 | 1.09E-08 | 0.45419 | 0.07604 | 37558883 |
|  | Cell-type-specific eQTL | B Cell | RP11-277P12.20 | 2.85E-09 | 0.60758 | 0.09758 | 37558883 |
|  | Cell-type-specific eQTL | B Cell | RP11-277P12.20 | 2.85E-09 | 0.60758 | 0.09758 | 37558883 |
|  | Cell-type-specific eQTL | B Cell | KLRK1 | 2.60E-08 | 0.70672 | 0.12184 | 37558883 |
|  | Cell-type-specific eQTL | B Cell | KLRK1 | 2.60E-08 | 0.70672 | 0.12184 | 37558883 |
|  | Cell-type-specific eQTL | Natural Killer Cell (NK) | KLRC3 | 9.31E-10 | 0.53799 | 0.08348 | 37558883 |
|  | Cell-type-specific eQTL | Natural Killer Cell (NK) | KLRK1 | 2.51E-05 | 0.14246 | 0.03297 | 37558883 |
|  | Cell-type-specific eQTL | Natural Killer Cell (NK) | KLRC3 | 3.88E-14 | 0.98349 | 0.12047 | 37558883 |
|  | Cell-type-specific eQTL | Natural Killer Cell (NK) | KLRC3 | 6.71E-12 | 0.55797 | 0.07638 | 37558883 |
|  | Cell-type-specific eQTL | Natural Killer Cell (NK) | KLRC3 | 6.71E-12 | 0.55797 | 0.07638 | 37558883 |
|  | Cell-type-specific eQTL | Natural Killer Cell (NK) | KLRC1 | 1.71E-07 | 0.42442 | 0.07823 | 37558883 |
|  | Cell-type-specific eQTL | Natural Killer Cell (NK) | KLRC2 | 3.25E-06 | 0.52168 | 0.10883 | 37558883 |
|  | Cell-type-specific eQTL | Natural Killer Cell (NK) | KLRC3 | 3.88E-14 | 0.98349 | 0.12047 | 37558883 |
|  | Cell-type-specific eQTL | Natural Killer Cell (NK) | KLRC2 | 1.03E-08 | 0.88888 | 0.14859 | 37558883 |
|  | Cell-type-specific eQTL | Natural Killer Cell (NK) | KLRC3 | 9.31E-10 | 0.53799 | 0.08348 | 37558883 |
|  | Cell-type-specific eQTL | Natural Killer Cell (NK) | KLRC2 | 2.63E-08 | 0.60875 | 0.10483 | 37558883 |
|  | Cell-type-specific eQTL | Natural Killer Cell (NK) | KLRC2 | 2.63E-08 | 0.60875 | 0.10483 | 37558883 |
|  | Cell-type-specific eQTL | Natural Killer Cell (NK) | KLRC1 | 1.22E-05 | 0.42381 | 0.09432 | 37558883 |
|  | Cell-type-specific eQTL | Natural Killer Cell (NK) | KLRC1 | 1.22E-05 | 0.42381 | 0.09432 | 37558883 |
|  | Cell-type-specific eQTL | CD8+ T Cell | KLRC4 | 9.42E-07 | -0.4228 | 0.08353 | 37558883 |
|  | Cell-type-specific eQTL | CD8+ T Cell | KLRC4 | 9.42E-07 | -0.4228 | 0.08353 | 37558883 |
|  | Cell-type-specific eQTL | CD8+ T Cell | KLRC4 | 0.000300707 | -0.33782 | 0.0918 | 37558883 |
|  | Cell-type-specific eQTL | Natural Killer Cell (NK) | KLRC1 | 1.71E-07 | 0.42442 | 0.07823 | 37558883 |
| rs6575931 | Cell-type-specific eQTL | Excitatory Neurons | TRAF3 | 6.10E-05 | 0.22713 | 0.05666 | 35915177 |
| rs3852751 | None | None | None | None | None | None | None |
| rs36001636 | None | None | None | None | None | None | None |
| rs1893592 | Cell-type-specific eQTL | CD4 Memory | UBASH3A | 1.08E-14 | 0.14707 | 0.01903 | 35545678 |
|  | Cell-type-specific eQTL | Memory T Cell | UBASH3A | 0.000632903 | -0.06837 | 0.02001 | 35545678 |
|  | Cell-type-specific eQTL | CD4+ T Cell | UBASH3A | 8.96E-12 | 1.04 | 0.15244 | 35213211 |
|  | Cell-type-specific eQTL | CD4+ T Cell | UBASH3A | 1.60E-11 | 0.98 | 0.14543 | 35213211 |
|  | Cell-type-specific eQTL | Memory T Cell | UBASH3A | 0.260115 | -0.04217 | 0.03745 | 35545678 |
|  | Cell-type-specific eQTL | CD8+ T Cell | UBASH3A | 2.66E-13 | 0.19129 | 0.02617 | 35545678 |
|  | Cell-type-specific eQTL | Memory Regulatory T | UBASH3A | 7.45E-13 | 1.01 | 0.14085 | 35213211 |
|  | Cell-type-specific eQTL | CD4+ T Cell | UBASH3A | 2.02E-12 | 1.03 | 0.14645 | 35213211 |
|  | Cell-type-specific eQTL | Memory T Cell | UBASH3A | 1.74E-16 | 0.15507 | 0.01882 | 35545678 |
|  | Cell-type-specific eQTL | CD4+ T Cell | UBASH3A | 8.99E-10 | 0.92 | 0.15017 | 35213211 |
|  | Cell-type-specific eQTL | Naive Regulatory T | UBASH3A | 3.09E-16 | 1.14 | 0.13954 | 35213211 |
|  | Cell-type-specific eQTL | Memory T Cell | UBASH3A | 0.00107793 | 0.07206 | 0.02204 | 35545678 |
|  | Cell-type-specific eQTL | Memory T Cell | UBASH3A | 0.312984 | 0.03051 | 0.03023 | 35545678 |
|  | Cell-type-specific eQTL | CD4+ T Cell | UBASH3A | 9.14E-08 | 0.12482 | 0.02336 | 35389781 |
|  | Cell-type-specific eQTL | CD4+ Naive T | UBASH3A | 3.67E-15 | 1.11 | 0.14112 | 35213211 |
|  | Cell-type-specific eQTL | CD8+ Naive T | UBASH3A | 2.72E-15 | 1.12 | 0.14172 | 35213211 |
|  | Cell-type-specific eQTL | CD4+ T Cell | UBASH3A | 1.65E-19 | 1.21 | 0.13393 | 35213211 |

SNP: Reference SNP identifier (rsID)

QTLtype: QTL category

cellTypeName: specific cell type

Gene: gene name

P-value: association significance

beta: effect size

se: standard error of beta

PMID: source dataset

Table S3. mQTLs for novel loci identified in the meta-analysis and conjunctional FDR analysis.

| **SNP** | **CHR** | **BP** | **Probe** | **Probe Chr** | **Probe bp** | **Gene** | **Orientation** | **beta** | **se** | ***P*-value** | **PMID** |
| --- | --- | --- | --- | --- | --- | --- | --- | --- | --- | --- | --- |
| rs1763839 | 1 | 8473336 | cg00546117 | 1 | 8445545 | RERE | + | -0.073 | 0.00168233 | 0 | 30401456 |
|  |  |  | cg15702277 | 1 | 8477935 | RERE | + | -0.006 | 0.000932541 | 5.02838E-10 | 30401456 |
|  |  |  | cg19465883 | 1 | 8484670 | RERE;LOC102724552 | + | -0.089 | 0.00410113 | 1.3488E-103 | 30401456 |
|  |  |  | cg16484858 | 1 | 8484694 | RERE;LOC102724552 | + | -0.078 | 0.00366088 | 7.5254E-102 | 30401456 |
|  |  |  | cg14004768 | 1 | 8484703 | RERE;LOC102724552 | + | -0.058 | 0.00272735 | 1.524E-100 | 30401456 |
|  |  |  | cg04317648 | 1 | 8485376 | LOC102724552;RERE | + | -0.025 | 0.00166082 | 8.23327E-51 | 30401456 |
|  |  |  | cg03281911 | 1 | 8498744 | RERE | + | -0.015 | 0.00215919 | 1.70904E-12 | 30401456 |
|  |  |  | cg08047233 | 1 | 8578167 | RERE | + | -0.012 | 0.00193276 | 5.93822E-10 | 30401456 |
|  |  |  | cg17442683 | 1 | 8664311 | RERE | + | 0.027 | 0.00193599 | 5.51429E-43 | 30401456 |
|  |  |  | cg02423433 | 1 | 8741157 | RERE | + | -0.014 | 0.00189993 | 8.40836E-13 | 30401456 |
|  |  |  | cg06159269 | 1 | 8767347 | RERE | + | -0.021 | 0.00207356 | 2.61228E-23 | 30401456 |
|  |  |  | cg19371916 | 1 | 8874984 | RERE | + | 0.010 | 0.0016835 | 1.14926E-09 | 30401456 |
|  |  |  | cg10211414 | 1 | 8875053 | RERE | + | 0.021 | 0.00247213 | 3.30814E-17 | 30401456 |
|  |  |  | cg07020871 | 1 | 8932179 | ENO1 | + | 0.010 | 0.00144864 | 1.72174E-11 | 30401456 |
|  |  |  | cg15867712 | 1 | 8932359 | ENO1 | + | 0.009 | 0.00128561 | 1.1682E-12 | 30401456 |
|  |  |  | cg22965541 | 1 | 8935061 | ENO1 | + | 0.018 | 0.00268716 | 2.01813E-11 | 30401456 |
|  |  |  | cg18273817 | 1 | 8936178 | ENO1 | + | 0.019 | 0.00302753 | 1.59471E-10 | 30401456 |
|  |  |  | cg06972019 | 1 | 8937448 | ENO1 | + | 0.015 | 0.00173232 | 4.86472E-19 | 30401456 |
|  |  |  | cg26857135 | 1 | 8972241 |  | + | -0.045 | 0.00565565 | 2.9449E-15 | 30401456 |
|  |  |  | cg00546117 | 1 | 8445545 |  | N | -1.016 | 0.0276927 | 8.2518E-295 | 30514905; 29500431 |
|  |  |  | cg00786138 | 1 | 8470722 |  | N | -0.203 | 0.0345997 | 4.55141E-09 | 30514905; 29500431 |
|  |  |  | cg01447281 | 1 | 8482689 |  | N | -0.219 | 0.0344688 | 2.26549E-10 | 30514905; 29500431 |
|  |  |  | cg00120948 | 1 | 8484417 |  | N | -0.310 | 0.0341902 | 1.26409E-19 | 30514905; 29500431 |
|  |  |  | cg17029193 | 1 | 8510607 |  | N | 0.201 | 0.0344335 | 4.97455E-09 | 30514905; 29500431 |
|  |  |  | cg23963229 | 1 | 8725074 |  | N | -0.223 | 0.0348281 | 1.57753E-10 | 30514905; 29500431 |
|  |  |  | cg06159269 | 1 | 8767347 |  | N | -0.310 | 0.0339517 | 6.323E-20 | 30514905; 29500431 |
|  |  |  | cg19371916 | 1 | 8874984 |  | N | 0.224 | 0.0346082 | 9.25538E-11 | 30514905; 29500431 |
|  |  |  | cg10211414 | 1 | 8875053 |  | N | 0.282 | 0.0340872 | 1.21585E-16 | 30514905; 29500431 |
|  |  |  | cg10443148 | 1 | 8922612 |  | N | 0.212 | 0.0339985 | 4.53352E-10 | 30514905; 29500431 |
|  |  |  | cg06972019 | 1 | 8937448 |  | N | 0.370 | 0.0338241 | 6.48582E-28 | 30514905; 29500431 |
|  |  |  | cg22352169 | 1 | 8394401 | SLC45A1 | + | -0.368 | 0.0528994 | 3.35489E-12 | 38548728 |
|  |  |  | cg00546117 | 1 | 8445545 | RERE | - | -1.416 | 0.0430297 | 1.6905E-237 | 38548728 |
|  |  |  | cg09888229 | 1 | 8487494 | RERE | - | 0.581 | 0.0517568 | 2.77424E-29 | 38548728 |
|  |  |  | cg06159269 | 1 | 8767347 | RERE | - | -0.722 | 0.0503447 | 1.21111E-46 | 38548728 |
|  |  |  | cg19371916 | 1 | 8874984 | RERE | - | 0.403 | 0.0526883 | 1.96903E-14 | 38548728 |
|  |  |  | cg10211414 | 1 | 8875053 | RERE | - | 0.453 | 0.0523854 | 5.27928E-18 | 38548728 |
|  |  |  | cg10313368 | 1 | 8935228 | ENO1 | - | 0.411 | 0.052846 | 7.17964E-15 | 38548728 |
|  |  |  | cg06972019 | 1 | 8937448 | ENO1-AS1 | + | 0.585 | 0.0526249 | 1.10335E-28 | 38548728 |
| rs7524764 | 1 | 157685739 | cg14420844 | 1 | 157550457 | FCRL4 | + | 0.005 | 0.000802391 | 2.2272E-09 | 30401456 |
|  |  |  | cg14453769 | 1 | 157596094 |  | + | -0.033 | 0.00359206 | 1.09103E-19 | 30401456 |
|  |  |  | cg25259754 | 1 | 157670220 | FCRL3 | + | -0.020 | 0.0018865 | 1.70652E-27 | 30401456 |
|  |  |  | cg17134153 | 1 | 157670328 | FCRL3 | + | -0.035 | 0.00261654 | 1.63589E-40 | 30401456 |
|  |  |  | cg01045635 | 1 | 157670481 | FCRL3 | + | -0.012 | 0.00167742 | 1.07997E-13 | 30401456 |
|  |  |  | cg19602479 | 1 | 157670869 | FCRL3 | + | -0.015 | 0.0022521 | 1.79203E-11 | 30401456 |
|  |  |  | cg21721331 | 1 | 157670877 | FCRL3 | + | -0.038 | 0.00286449 | 2.37366E-39 | 30401456 |
|  |  |  | cg25445846 | 1 | 157743759 | FCRL2 | + | -0.025 | 0.00154659 | 9.14831E-57 | 30401456 |
|  |  |  | cg18268488 | 1 | 157545234 |  | N | 0.359 | 0.0438949 | 2.76511E-16 | 30514905; 29500431 |
|  |  |  | cg25259754 | 1 | 157670220 |  | N | -0.279 | 0.0445887 | 4.06593E-10 | 30514905; 29500431 |
|  |  |  | cg17134153 | 1 | 157670328 |  | N | -0.613 | 0.0431586 | 8.92338E-46 | 30514905; 29500431 |
|  |  |  | cg18268488 | 1 | 157545234 | FCRL4 | - | 0.421 | 0.0347432 | 8.09977E-34 | 38548728 |
|  |  |  | cg25259754 | 1 | 157670220 | FCRL3 | - | -0.436 | 0.0346215 | 2.30098E-36 | 38548728 |
|  |  |  | cg17134153 | 1 | 157670328 | FCRL3 | - | -0.689 | 0.0324053 | 1.9246E-100 | 38548728 |
|  |  |  | cg08786003 | 1 | 157670710 | FCRL3 | - | -0.208 | 0.0355884 | 5.22137E-09 | 38548728 |
|  |  |  | cg19602479 | 1 | 157670869 | FCRL3 | - | -0.231 | 0.0354825 | 7.59454E-11 | 38548728 |
| rs3818813 | 1 | 157718325 | cg14453769 | 1 | 157596094 |  | + | -0.030 | 0.00368687 | 1.70507E-16 | 30401456 |
|  |  |  | cg25259754 | 1 | 157670220 | FCRL3 | + | -0.019 | 0.00194418 | 4.22114E-22 | 30401456 |
|  |  |  | cg17134153 | 1 | 157670328 | FCRL3 | + | -0.031 | 0.00272063 | 4.35605E-30 | 30401456 |
|  |  |  | cg01045635 | 1 | 157670481 | FCRL3 | + | -0.012 | 0.0017188 | 2.12492E-11 | 30401456 |
|  |  |  | cg21721331 | 1 | 157670877 | FCRL3 | + | -0.035 | 0.0029591 | 1.8878E-32 | 30401456 |
|  |  |  | cg25445846 | 1 | 157743759 | FCRL2 | + | -0.027 | 0.00153967 | 2.5487E-71 | 30401456 |
|  |  |  | cg18268488 | 1 | 157545234 |  | N | 0.291 | 0.0453403 | 1.33044E-10 | 30514905; 29500431 |
|  |  |  | cg17134153 | 1 | 157670328 |  | N | -0.529 | 0.0442965 | 6.41506E-33 | 30514905; 29500431 |
|  |  |  | cg18268488 | 1 | 157545234 | FCRL4 | - | 0.343 | 0.0338399 | 3.72708E-24 | 38548728 |
|  |  |  | cg25259754 | 1 | 157670220 | FCRL3 | - | -0.444 | 0.033325 | 1.88595E-40 | 38548728 |
|  |  |  | cg17134153 | 1 | 157670328 | FCRL3 | - | -0.708 | 0.0309166 | 3.6632E-116 | 38548728 |
|  |  |  | cg19602479 | 1 | 157670869 | FCRL3 | - | -0.247 | 0.034229 | 5.15147E-13 | 38548728 |
| rs3771258 | 2 | 61764140 | cg15644097 | 2 | 61373028 | LOC339803;C2orf74 | + | 0.020 | 0.00240374 | 3.49556E-17 | 30401456 |
|  |  |  | cg27356779 | 2 | 61393225 |  | + | 0.028 | 0.0021856 | 2.22906E-36 | 30401456 |
|  |  |  | cg03313140 | 2 | 61403611 | AHSA2 | + | 0.015 | 0.00245243 | 4.92796E-10 | 30401456 |
|  |  |  | cg16084286 | 2 | 61417648 | USP34 | + | 0.037 | 0.00216441 | 2.22333E-64 | 30401456 |
|  |  |  | cg24698774 | 2 | 61562609 | USP34 | + | -0.009 | 0.00148673 | 7.24485E-09 | 30401456 |
|  |  |  | cg01923102 | 2 | 61583672 | USP34 | + | -0.018 | 0.00230945 | 3.41553E-14 | 30401456 |
|  |  |  | cg02054108 | 2 | 61607478 | USP34 | + | -0.015 | 0.00144048 | 1.16305E-25 | 30401456 |
|  |  |  | cg06854882 | 2 | 61646507 | USP34 | + | -0.021 | 0.00153553 | 2.18561E-41 | 30401456 |
|  |  |  | cg18970362 | 2 | 61650995 | USP34 | + | -0.015 | 0.00162886 | 9.06026E-21 | 30401456 |
|  |  |  | cg09180213 | 2 | 61713241 | XPO1 | + | -0.010 | 0.0014577 | 7.82452E-13 | 30401456 |
|  |  |  | cg22001211 | 2 | 61766532 | XPO1 | + | 0.005 | 0.000768301 | 1.77205E-09 | 30401456 |
|  |  |  | cg04132236 | 2 | 61824961 |  | + | -0.011 | 0.00126889 | 5.52158E-17 | 30401456 |
|  |  |  | cg24686644 | 2 | 62020921 |  | + | -0.016 | 0.00283786 | 6.1432E-09 | 30401456 |
|  |  |  | cg10580144 | 2 | 61372316 |  | N | -0.198 | 0.0335382 | 3.37306E-09 | 30514905; 29500431 |
|  |  |  | cg03313140 | 2 | 61403611 |  | N | 0.202 | 0.0335067 | 1.55108E-09 | 30514905; 29500431 |
|  |  |  | cg15711740 | 2 | 61764176 |  | N | 0.279 | 0.0322065 | 4.39261E-18 | 30514905; 29500431 |
|  |  |  | cg22001211 | 2 | 61766532 |  | N | 0.200 | 0.0330415 | 1.43679E-09 | 30514905; 29500431 |
|  |  |  | cg04541421 | 2 | 61370764 | LOC339803 | - | 0.461 | 0.029327 | 1.0534E-55 | 38548728 |
|  |  |  | cg06949933 | 2 | 61406491 | AHSA2 | + | 0.229 | 0.0307726 | 1.10024E-13 | 38548728 |
|  |  |  | cg22934200 | 2 | 61407270 | AHSA2 | + | 0.205 | 0.0304007 | 1.4185E-11 | 38548728 |
|  |  |  | cg02054108 | 2 | 61607478 | SNORA70B | - | -0.507 | 0.0290093 | 1.68211E-68 | 38548728 |
|  |  |  | cg15711740 | 2 | 61764176 | XPO1 | - | 0.278 | 0.030696 | 1.30703E-19 | 38548728 |
|  |  |  | cg22001211 | 2 | 61766532 | XPO1 | - | 0.207 | 0.0308473 | 2.06524E-11 | 38548728 |
|  |  |  | cg04132236 | 2 | 61824961 | XPO1 | - | -0.248 | 0.0306883 | 7.27926E-16 | 38548728 |
|  |  |  | cg07589899 | 2 | 62020677 | FAM161A | - | -0.238 | 0.030702 | 1.01585E-14 | 38548728 |
|  |  |  | cg24686644 | 2 | 62020921 | FAM161A | - | -0.249 | 0.0306885 | 4.67991E-16 | 38548728 |
| rs231805 | 2 | 204708749 | cg05092371 | 2 | 204731519 |  | N | -1.325 | 0.038665 | 1.7129E-257 | 30514905; 29500431 |
|  |  |  | cg05092371 | 2 | 204731519 | CTLA4 | + | -1.362 | 0.0307511 | 0 | 38548728 |
| rs13429408 | 2 | 219142860 | cg14150666 | 2 | 218991310 | CXCR2 | + | 0.033 | 0.00164375 | 1.4302E-91 | 30401456 |
|  |  |  | cg04731861 | 2 | 219085781 | ARPC2 | + | 0.031 | 0.00153733 | 1.09596E-92 | 30401456 |
|  |  |  | cg01094660 | 2 | 219097708 | ARPC2 | + | 0.021 | 0.00148591 | 9.16746E-44 | 30401456 |
|  |  |  | cg20190597 | 2 | 219119100 |  | + | 0.016 | 0.000932754 | 6.68472E-64 | 30401456 |
|  |  |  | cg25124402 | 2 | 219125749 | GPBAR1 | + | -0.007 | 0.00128842 | 7.29723E-09 | 30401456 |
|  |  |  | cg20019365 | 2 | 219134978 | PNKD;AAMP | + | -0.004 | 0.000341775 | 1.79813E-26 | 30401456 |
|  |  |  | cg23569941 | 2 | 219139048 | TMBIM1;PNKD | + | 0.019 | 0.00251119 | 1.2511E-13 | 30401456 |
|  |  |  | cg13835894 | 2 | 219148914 | PNKD;TMBIM1 | + | -0.018 | 0.00125576 | 5.70618E-48 | 30401456 |
|  |  |  | cg05083539 | 2 | 219150776 | PNKD;TMBIM1 | + | 0.007 | 0.0010232 | 1.40507E-12 | 30401456 |
|  |  |  | cg24578623 | 2 | 219152149 | PNKD;TMBIM1 | + | 0.006 | 0.00103029 | 4.99639E-09 | 30401456 |
|  |  |  | cg05273171 | 2 | 219154857 | PNKD;TMBIM1 | + | 0.010 | 0.000944846 | 2.47E-25 | 30401456 |
|  |  |  | cg18259342 | 2 | 219184859 | PNKD | + | -0.044 | 0.00160052 | 4.6312E-166 | 30401456 |
|  |  |  | cg22712983 | 2 | 219187374 | PNKD | + | -0.043 | 0.00240705 | 3.86295E-71 | 30401456 |
|  |  |  | cg15100426 | 2 | 219187432 | PNKD | + | -0.020 | 0.00175179 | 5.86868E-29 | 30401456 |
|  |  |  | cg26786924 | 2 | 219187654 | PNKD | + | -0.004 | 0.000681087 | 6.26367E-09 | 30401456 |
|  |  |  | cg04018708 | 2 | 219188804 | PNKD | + | -0.009 | 0.000769097 | 1.03355E-29 | 30401456 |
|  |  |  | cg04880052 | 2 | 219191631 | PNKD | + | -0.007 | 0.0011181 | 1.9818E-09 | 30401456 |
|  |  |  | cg04863746 | 2 | 219198921 | PNKD;CATIP-AS2 | + | 0.043 | 0.00259658 | 2.78092E-60 | 30401456 |
|  |  |  | cg07203607 | 2 | 219222315 | CATIP;CATIP-AS2 | + | 0.015 | 0.00134111 | 2.44927E-27 | 30401456 |
|  |  |  | cg04454831 | 2 | 219249000 | SLC11A1 | + | 0.018 | 0.00202103 | 2.3005E-18 | 30401456 |
|  |  |  | cg06547715 | 2 | 218990976 |  | N | 0.369 | 0.0324977 | 8.34635E-30 | 30514905; 29500431 |
|  |  |  | cg14150666 | 2 | 218991310 |  | N | 0.660 | 0.0305144 | 9.5625E-104 | 30514905; 29500431 |
|  |  |  | cg06608945 | 2 | 219082296 |  | N | -0.427 | 0.0309375 | 2.95072E-43 | 30514905; 29500431 |
|  |  |  | cg04731861 | 2 | 219085781 |  | N | 0.578 | 0.031361 | 6.82739E-76 | 30514905; 29500431 |
|  |  |  | cg25124402 | 2 | 219125749 |  | N | -0.213 | 0.0317939 | 1.95942E-11 | 30514905; 29500431 |
|  |  |  | cg13835894 | 2 | 219148914 |  | N | -0.405 | 0.0324333 | 7.25919E-36 | 30514905; 29500431 |
|  |  |  | cg18259342 | 2 | 219184859 |  | N | -0.745 | 0.0297492 | 1.7424E-138 | 30514905; 29500431 |
|  |  |  | cg05991184 | 2 | 219186017 |  | N | 0.202 | 0.0326392 | 6.3585E-10 | 30514905; 29500431 |
|  |  |  | cg22712983 | 2 | 219187374 |  | N | -0.336 | 0.0326635 | 8.72053E-25 | 30514905; 29500431 |
|  |  |  | cg15100426 | 2 | 219187432 |  | N | -0.191 | 0.0329704 | 7.26281E-09 | 30514905; 29500431 |
|  |  |  | cg26786924 | 2 | 219187654 |  | N | -0.324 | 0.0325849 | 2.35871E-23 | 30514905; 29500431 |
|  |  |  | cg04880052 | 2 | 219191631 |  | N | -0.312 | 0.0323502 | 4.69556E-22 | 30514905; 29500431 |
|  |  |  | cg04066495 | 2 | 219222123 |  | N | 0.626 | 0.0308826 | 3.14143E-91 | 30514905; 29500431 |
|  |  |  | cg13215060 | 2 | 219229146 |  | N | -0.695 | 0.0297085 | 3.5544E-121 | 30514905; 29500431 |
|  |  |  | cg06547715 | 2 | 218990976 | CXCR2 | + | 0.452 | 0.0307611 | 7.3176E-49 | 38548728 |
|  |  |  | cg14150666 | 2 | 218991310 | CXCR2 | + | 0.493 | 0.0303888 | 3.25618E-59 | 38548728 |
|  |  |  | cg19141132 | 2 | 219080920 | ARPC2 | + | 0.184 | 0.0315972 | 5.51951E-09 | 38548728 |
|  |  |  | cg08216808 | 2 | 219081385 | ARPC2 | + | 0.333 | 0.0312373 | 1.83286E-26 | 38548728 |
|  |  |  | cg00012203 | 2 | 219082015 | ARPC2 | + | -0.826 | 0.0274968 | 1.8444E-198 | 38548728 |
|  |  |  | cg04731861 | 2 | 219085781 | ARPC2 | + | 1.057 | 0.0233967 | 0 | 38548728 |
|  |  |  | cg25124402 | 2 | 219125749 | GPBAR1 | + | -0.232 | 0.0316072 | 2.05768E-13 | 38548728 |
|  |  |  | cg20019365 | 2 | 219134978 |  | * | -0.352 | 0.0309366 | 6.08779E-30 | 38548728 |
|  |  |  | cg23569941 | 2 | 219139048 | PNKD | + | 0.497 | 0.030437 | 5.62907E-60 | 38548728 |
|  |  |  | cg13835894 | 2 | 219148914 | TMBIM1 | - | -0.595 | 0.0296386 | 1.04159E-89 | 38548728 |
|  |  |  | cg01329789 | 2 | 219157515 | TMBIM1 | - | -0.242 | 0.0315718 | 1.68546E-14 | 38548728 |
|  |  |  | cg18259342 | 2 | 219184859 | PNKD | + | -1.067 | 0.0231666 | 0 | 38548728 |
|  |  |  | cg05991184 | 2 | 219186017 | PNKD | + | 0.293 | 0.0314188 | 1.18859E-20 | 38548728 |
|  |  |  | cg22712983 | 2 | 219187374 | PNKD | + | -0.553 | 0.0297928 | 6.27035E-77 | 38548728 |
|  |  |  | cg15100426 | 2 | 219187432 | PNKD | + | -0.315 | 0.0314179 | 1.01073E-23 | 38548728 |
|  |  |  | cg26786924 | 2 | 219187654 | PNKD | + | -0.430 | 0.0307146 | 1.47323E-44 | 38548728 |
|  |  |  | cg04880052 | 2 | 219191631 | PNKD | + | -0.240 | 0.0317745 | 4.25706E-14 | 38548728 |
|  |  |  | cg04066495 | 2 | 219222123 | CATIP | + | 0.669 | 0.0288439 | 5.9925E-119 | 38548728 |
|  |  |  | cg13215060 | 2 | 219229146 | CATIP-AS1 | - | -0.413 | 0.0311023 | 3.25402E-40 | 38548728 |
| rs34463936 | 5 | 35850149 | cg06790244 | 5 | 35815845 |  | + | -0.040 | 0.00350561 | 8.72978E-31 | 30401456 |
|  |  |  | cg27582180 | 5 | 35852286 |  | + | -0.007 | 0.00103758 | 1.34665E-11 | 30401456 |
|  |  |  | cg02771489 | 5 | 35852768 |  | + | 0.010 | 0.00100444 | 3.36748E-25 | 30401456 |
|  |  |  | cg09065326 | 5 | 35853926 |  | + | -0.010 | 0.00156049 | 2.26905E-10 | 30401456 |
|  |  |  | cg04213565 | 5 | 35900039 |  | + | -0.011 | 0.00179814 | 7.2745E-10 | 30401456 |
|  |  |  | cg13894535 | 5 | 35919491 | CAPSL | + | -0.024 | 0.00197016 | 1.26739E-34 | 30401456 |
|  |  |  | cg13894535 | 5 | 35919491 |  | N | -0.378 | 0.0358524 | 6.05231E-26 | 30514905; 29500431 |
|  |  |  | cg10994379 | 5 | 35939420 |  | N | 0.238 | 0.0361038 | 4.13327E-11 | 30514905; 29500431 |
|  |  |  | cg13894535 | 5 | 35919491 | CAPSL | - | -0.335 | 0.0427503 | 4.81539E-15 | 38548728 |
|  |  |  | cg10994379 | 5 | 35939420 | LOC100506406 | + | 0.304 | 0.0430281 | 1.55884E-12 | 38548728 |
| rs10213865 | 5 | 35857850 | cg06790244 | 5 | 35815845 |  | + | -0.039 | 0.00351962 | 3.50503E-29 | 30401456 |
|  |  |  | cg06790244 | 5 | 35815845 |  | + | -0.039 | 0.00351962 | 3.50503E-29 | 30401456 |
|  |  |  | cg27582180 | 5 | 35852286 |  | + | -0.007 | 0.00103869 | 1.48822E-11 | 30401456 |
|  |  |  | cg27582180 | 5 | 35852286 |  | + | -0.007 | 0.00103869 | 1.48822E-11 | 30401456 |
|  |  |  | cg02771489 | 5 | 35852768 |  | + | 0.010 | 0.00100657 | 1.33422E-24 | 30401456 |
|  |  |  | cg02771489 | 5 | 35852768 |  | + | 0.010 | 0.00100657 | 1.33422E-24 | 30401456 |
|  |  |  | cg09065326 | 5 | 35853926 |  | + | -0.010 | 0.00156403 | 1.0029E-09 | 30401456 |
|  |  |  | cg09065326 | 5 | 35853926 |  | + | -0.010 | 0.00156403 | 1.0029E-09 | 30401456 |
|  |  |  | cg04213565 | 5 | 35900039 |  | + | -0.011 | 0.00179882 | 3.64871E-10 | 30401456 |
|  |  |  | cg04213565 | 5 | 35900039 |  | + | -0.011 | 0.00179882 | 3.64871E-10 | 30401456 |
|  |  |  | cg13894535 | 5 | 35919491 | CAPSL | + | -0.024 | 0.00197105 | 6.56742E-35 | 30401456 |
|  |  |  | cg13894535 | 5 | 35919491 | CAPSL | + | -0.024 | 0.00197105 | 6.56742E-35 | 30401456 |
|  |  |  | cg25377862 | 5 | 36071462 |  | + | 0.018 | 0.00311453 | 5.5645E-09 | 30401456 |
|  |  |  | cg25377862 | 5 | 36071462 |  | + | 0.018 | 0.00311453 | 5.5645E-09 | 30401456 |
|  |  |  | cg13894535 | 5 | 35919491 |  | N | -0.398 | 0.0360722 | 2.54701E-28 | 30514905; 29500431 |
|  |  |  | cg10994379 | 5 | 35939420 |  | N | 0.243 | 0.0364316 | 2.52655E-11 | 30514905; 29500431 |
|  |  |  | cg27582180 | 5 | 35852286 | IL7R | + | -0.218 | 0.0381139 | 1.07555E-08 | 38548728 |
|  |  |  | cg04312209 | 5 | 35857130 | IL7R | + | -0.227 | 0.0376177 | 1.71248E-09 | 38548728 |
|  |  |  | cg13894535 | 5 | 35919491 | CAPSL | - | -0.217 | 0.038076 | 1.25105E-08 | 38548728 |
|  |  |  | cg10994379 | 5 | 35939420 | LOC100506406 | + | 0.340 | 0.0378696 | 2.84966E-19 | 38548728 |
| rs4484457 (Proxy variant of rs10213865 (LD [r2] = 0.42)) | 5 | 35915661 | cg06790244 | 5 | 35815845 |  | + | -0.048 | 0.00303407 | 9.90596E-56 | 30401456 |
|  |  |  | cg09065326 | 5 | 35853926 |  | + | -0.010 | 0.00140704 | 2.98181E-12 | 30401456 |
|  |  |  | cg01183517 | 5 | 35899347 |  | + | 0.015 | 0.00161788 | 3.19062E-20 | 30401456 |
|  |  |  | cg04213565 | 5 | 35900039 |  | + | -0.015 | 0.00159297 | 9.90328E-21 | 30401456 |
|  |  |  | cg13894535 | 5 | 35919491 | CAPSL;CAPSL | + | -0.028 | 0.00169658 | 6.6127E-63 | 30401456 |
|  |  |  | cg13894535 | 5 | 35919491 |  | N | -0.449 | 0.0318833 | 4.4815E-45 | 30514905; 29500431 |
|  |  |  | cg13894535 | 5 | 35919491 | CAPSL | - | 0.184 | 0.0315568 | 5.09241E-09 | 38548728 |
|  |  |  | cg18511008 | 5 | 35925474 | CAPSL | - | 0.455 | 0.0304004 | 1.05429E-50 | 38548728 |
|  |  |  | cg19698137 | 5 | 35990997 | UGT3A1 | - | 0.266 | 0.0311013 | 1.28832E-17 | 38548728 |
|  |  |  | cg19897071 | 5 | 35991382 | UGT3A1 | - | 0.189 | 0.0316932 | 2.32329E-09 | 38548728 |
| rs77973332 | 10 | 64881009 | cg25356468 | 10 | 64875536 | NRBF2 | + | 0.277 | 0.040339 | 6.33836E-12 | 38548728 |
|  |  |  | cg01528832 | 10 | 65225240 | JMJD1C-AS1 | + | 0.462 | 0.0402507 | 1.66519E-30 | 38548728 |
| rs57943165 | 10 | 65362966 | cg01528832 | 10 | 65225240 | JMJD1C-AS1 | + | 0.453 | 0.0390774 | 4.31166E-31 | 38548728 |
|  |  |  | cg22797468 | 10 | 65364720 | REEP3 | + | 0.282 | 0.0391004 | 5.94784E-13 | 38548728 |
| rs1250564 | 10 | 81047342 | cg06073814 | 10 | 80983818 | ZMIZ1 | + | 0.042 | 0.0050455 | 1.92115E-16 | 30401456 |
|  |  |  | cg18737081 | 10 | 80999807 | ZMIZ1 | + | -0.032 | 0.00358582 | 1.10632E-18 | 30401456 |
|  |  |  | cg20744163 | 10 | 80999841 | ZMIZ1 | + | -0.060 | 0.00660605 | 1.41152E-19 | 30401456 |
|  |  |  | cg07056602 | 10 | 81004884 | ZMIZ1 | + | -0.010 | 0.00114738 | 8.72981E-19 | 30401456 |
|  |  |  | cg13928309 | 10 | 81008284 | ZMIZ1 | + | -0.008 | 0.00118294 | 8.95073E-13 | 30401456 |
|  |  |  | cg13526706 | 10 | 81009815 | ZMIZ1 | + | -0.009 | 0.00114526 | 2.94263E-16 | 30401456 |
|  |  |  | cg17753789 | 10 | 81026766 | ZMIZ1 | + | -0.008 | 0.00130232 | 4.17168E-09 | 30401456 |
|  |  |  | cg06259934 | 10 | 81034374 | ZMIZ1 | + | -0.026 | 0.00128568 | 5.71105E-90 | 30401456 |
|  |  |  | cg03347973 | 10 | 81034505 | ZMIZ1 | + | -0.021 | 0.00133759 | 1.54684E-56 | 30401456 |
|  |  |  | cg26483294 | 10 | 81042515 | ZMIZ1 | + | 0.013 | 0.0010601 | 2.38688E-32 | 30401456 |
|  |  |  | cg17100355 | 10 | 81043875 | ZMIZ1 | + | 0.052 | 0.0014038 | 1.8654E-300 | 30401456 |
|  |  |  | cg23689050 | 10 | 81044831 | ZMIZ1 | + | -0.019 | 0.00129796 | 3.05335E-49 | 30401456 |
|  |  |  | cg09955892 | 10 | 81044855 | ZMIZ1 | + | -0.010 | 0.0012497 | 2.95929E-16 | 30401456 |
|  |  |  | cg04609265 | 10 | 81045085 | ZMIZ1 | + | -0.019 | 0.00129412 | 1.55163E-49 | 30401456 |
|  |  |  | cg02795981 | 10 | 81045119 | ZMIZ1 | + | -0.020 | 0.00136285 | 8.88247E-49 | 30401456 |
|  |  |  | cg13574913 | 10 | 81045166 | ZMIZ1 | + | -0.034 | 0.00136785 | 6.6117E-135 | 30401456 |
|  |  |  | cg13115165 | 10 | 81045939 | ZMIZ1 | + | -0.034 | 0.00119388 | 5.978E-178 | 30401456 |
|  |  |  | cg06373032 | 10 | 81046101 | ZMIZ1 | + | -0.016 | 0.000660189 | 3.9138E-132 | 30401456 |
|  |  |  | cg01021283 | 10 | 81046551 | ZMIZ1 | + | -0.012 | 0.000577392 | 5.87272E-96 | 30401456 |
|  |  |  | cg17889259 | 10 | 81048344 | ZMIZ1 | + | -0.014 | 0.00140339 | 3.4984E-23 | 30401456 |
|  |  |  | cg18737081 | 10 | 80999807 |  | N | -0.356 | 0.0326538 | 1.05204E-27 | 30514905; 29500431 |
|  |  |  | cg20744163 | 10 | 80999841 |  | N | -0.378 | 0.0324618 | 2.91872E-31 | 30514905; 29500431 |
|  |  |  | cg14371731 | 10 | 81003175 |  | N | -0.188 | 0.0330704 | 1.31356E-08 | 30514905; 29500431 |
|  |  |  | cg20543544 | 10 | 81003657 |  | N | -0.196 | 0.0337241 | 5.80637E-09 | 30514905; 29500431 |
|  |  |  | cg19215199 | 10 | 81005284 |  | N | -0.182 | 0.0331742 | 3.79521E-08 | 30514905; 29500431 |
|  |  |  | cg17753789 | 10 | 81026766 |  | N | -0.219 | 0.0329177 | 2.97392E-11 | 30514905; 29500431 |
|  |  |  | cg06259934 | 10 | 81034374 |  | N | -0.344 | 0.0329301 | 1.36393E-25 | 30514905; 29500431 |
|  |  |  | cg02795981 | 10 | 81045119 |  | N | -0.291 | 0.0331759 | 1.89225E-18 | 30514905; 29500431 |
|  |  |  | cg08514558 | 10 | 81106712 |  | N | -0.185 | 0.033266 | 2.90889E-08 | 30514905; 29500431 |
|  |  |  | cg18737081 | 10 | 80999807 | PPIF | + | 0.338 | 0.0302158 | 4.01741E-29 | 38548728 |
|  |  |  | cg20744163 | 10 | 80999841 | PPIF | + | 0.346 | 0.0301954 | 1.83475E-30 | 38548728 |
|  |  |  | cg14371731 | 10 | 81003175 | PPIF | + | 0.205 | 0.030417 | 1.50874E-11 | 38548728 |
|  |  |  | cg20543544 | 10 | 81003657 | PPIF | + | 0.253 | 0.0304803 | 1.14298E-16 | 38548728 |
|  |  |  | cg19215199 | 10 | 81005284 | PPIF | + | 0.415 | 0.0297811 | 3.59005E-44 | 38548728 |
|  |  |  | cg06308720 | 10 | 81006066 | PPIF | + | 0.309 | 0.0301274 | 1.0212E-24 | 38548728 |
|  |  |  | cg17753789 | 10 | 81026766 | PPIF | + | 0.204 | 0.0305282 | 2.40193E-11 | 38548728 |
|  |  |  | cg06259934 | 10 | 81034374 | PPIF | + | 0.721 | 0.0273795 | 7.7956E-153 | 38548728 |
|  |  |  | cg02795981 | 10 | 81045119 | PPIF | + | 0.467 | 0.0295266 | 3.01389E-56 | 38548728 |
|  |  |  | cg10099930 | 10 | 81082189 | PPIF | + | 0.173 | 0.0303091 | 1.11999E-08 | 38548728 |
| rs11189178 | 10 | 99143272 | cg12074985 | 10 | 99080756 | FRAT1 | + | 0.010 | 0.00116285 | 7.54059E-17 | 30401456 |
|  |  |  | cg16501237 | 10 | 99081016 | FRAT1 | + | 0.020 | 0.00303467 | 7.23771E-11 | 30401456 |
|  |  |  | cg07529834 | 10 | 99092337 | FRAT2 | + | -0.009 | 0.0014155 | 7.50244E-11 | 30401456 |
|  |  |  | cg24590430 | 10 | 99097076 |  | + | -0.026 | 0.00236215 | 2.89611E-27 | 30401456 |
|  |  |  | cg13630239 | 10 | 99116547 | RRP12 | + | -0.043 | 0.00166192 | 1.6282E-145 | 30401456 |
|  |  |  | cg25862470 | 10 | 99148114 | RRP12 | + | 0.030 | 0.00124635 | 4.6025E-125 | 30401456 |
|  |  |  | cg06359132 | 10 | 99160096 | RRP12 | + | -0.033 | 0.0031047 | 3.44103E-26 | 30401456 |
|  |  |  | cg20016023 | 10 | 99160130 | RRP12 | + | -0.046 | 0.0026065 | 1.17461E-70 | 30401456 |
|  |  |  | cg04175911 | 10 | 99172893 |  | + | -0.007 | 0.00122673 | 4.55244E-09 | 30401456 |
|  |  |  | cg25507584 | 10 | 99173832 |  | + | -0.014 | 0.00114647 | 5.81587E-33 | 30401456 |
|  |  |  | cg26545845 | 10 | 99175095 |  | + | 0.011 | 0.0013073 | 9.00582E-18 | 30401456 |
|  |  |  | cg07052063 | 10 | 99255236 | MMS19 | + | 0.016 | 0.00201385 | 7.29809E-15 | 30401456 |
|  |  |  | cg17107501 | 10 | 98982314 |  | N | -0.255 | 0.0341635 | 8.64638E-14 | 30514905; 29500431 |
|  |  |  | cg16271424 | 10 | 99080594 |  | N | 0.243 | 0.0338722 | 8.00856E-13 | 30514905; 29500431 |
|  |  |  | cg12074985 | 10 | 99080756 |  | N | 0.345 | 0.0337276 | 1.26634E-24 | 30514905; 29500431 |
|  |  |  | cg10705379 | 10 | 99080932 |  | N | 0.316 | 0.0339636 | 1.26984E-20 | 30514905; 29500431 |
|  |  |  | cg16501237 | 10 | 99081016 |  | N | 0.323 | 0.0337844 | 1.06445E-21 | 30514905; 29500431 |
|  |  |  | cg20702121 | 10 | 99090072 |  | N | -0.495 | 0.0335826 | 2.96113E-49 | 30514905; 29500431 |
|  |  |  | cg24590430 | 10 | 99097076 |  | N | -0.310 | 0.0340042 | 7.86601E-20 | 30514905; 29500431 |
|  |  |  | cg13630239 | 10 | 99116547 |  | N | -0.668 | 0.0316089 | 4.862E-99 | 30514905; 29500431 |
|  |  |  | cg06359132 | 10 | 99160096 |  | N | -0.459 | 0.0337198 | 4.118E-42 | 30514905; 29500431 |
|  |  |  | cg20016023 | 10 | 99160130 |  | N | -0.675 | 0.0322814 | 4.26656E-97 | 30514905; 29500431 |
|  |  |  | cg08345082 | 10 | 99160200 |  | N | -0.513 | 0.0333197 | 1.64789E-53 | 30514905; 29500431 |
|  |  |  | cg07052063 | 10 | 99255236 |  | N | 0.249 | 0.0336463 | 1.44197E-13 | 30514905; 29500431 |
|  |  |  | cg16111448 | 10 | 99372353 |  | N | -0.185 | 0.0336819 | 4.26475E-08 | 30514905; 29500431 |
|  |  |  | cg16501237 | 10 | 99081016 | FRAT1 | + | 0.205 | 0.036333 | 1.62653E-08 | 38548728 |
|  |  |  | cg20702121 | 10 | 99090072 | FRAT2 | - | -0.709 | 0.0340685 | 3.17341E-96 | 38548728 |
|  |  |  | cg13680696 | 10 | 99092708 | FRAT2 | - | -0.207 | 0.0362558 | 1.13644E-08 | 38548728 |
|  |  |  | cg24590430 | 10 | 99097076 | FRAT2 | - | -0.690 | 0.0337787 | 7.85278E-93 | 38548728 |
|  |  |  | cg13630239 | 10 | 99116547 | FRAT2 | - | -1.083 | 0.0296469 | 5.3715E-292 | 38548728 |
|  |  |  | cg06359132 | 10 | 99160096 | RRP12 | - | -0.695 | 0.033762 | 2.95324E-94 | 38548728 |
|  |  |  | cg20016023 | 10 | 99160130 | RRP12 | - | -0.920 | 0.0315837 | 1.5902E-186 | 38548728 |
|  |  |  | cg08345082 | 10 | 99160200 | RRP12 | - | -0.718 | 0.0337895 | 2.6387E-100 | 38548728 |
|  |  |  | cg25603927 | 10 | 99185264 | PGAM1 | + | 0.238 | 0.0363618 | 5.97947E-11 | 38548728 |
|  |  |  | cg07151406 | 10 | 99185290 | PGAM1 | + | 0.311 | 0.0363823 | 1.24367E-17 | 38548728 |
|  |  |  | cg07221526 | 10 | 99185307 | PGAM1 | + | 0.292 | 0.0364866 | 1.12147E-15 | 38548728 |
|  |  |  | cg07052063 | 10 | 99255236 | MMS19 | - | 0.272 | 0.0360956 | 4.98322E-14 | 38548728 |
|  |  |  | cg25247692 | 10 | 99313390 | ANKRD2 | + | 0.252 | 0.0364259 | 4.4529E-12 | 38548728 |
| rs7895695 | 10 | 99167255 | cg07529834 | 10 | 99092337 | FRAT2 | + | -0.011 | 0.00145056 | 1.16134E-14 | 30401456 |
|  |  |  | cg07529834 | 10 | 99092337 | FRAT2 | + | -0.011 | 0.00145056 | 1.16134E-14 | 30401456 |
|  |  |  | cg07529834 | 10 | 99092337 | FRAT2 | + | -0.011 | 0.00145056 | 1.16134E-14 | 30401456 |
|  |  |  | cg24590430 | 10 | 99097076 |  | + | -0.033 | 0.00235895 | 1.86594E-45 | 30401456 |
|  |  |  | cg24590430 | 10 | 99097076 |  | + | -0.033 | 0.00235895 | 1.86594E-45 | 30401456 |
|  |  |  | cg24590430 | 10 | 99097076 |  | + | -0.033 | 0.00235895 | 1.86594E-45 | 30401456 |
|  |  |  | cg13630239 | 10 | 99116547 | RRP12 | + | -0.049 | 0.00159762 | 6.0374E-204 | 30401456 |
|  |  |  | cg13630239 | 10 | 99116547 | RRP12 | + | -0.049 | 0.00159762 | 6.0374E-204 | 30401456 |
|  |  |  | cg13630239 | 10 | 99116547 | RRP12 | + | -0.049 | 0.00159762 | 6.0374E-204 | 30401456 |
|  |  |  | cg25862470 | 10 | 99148114 | RRP12 | + | 0.025 | 0.00139497 | 2.01843E-71 | 30401456 |
|  |  |  | cg25862470 | 10 | 99148114 | RRP12 | + | 0.025 | 0.00139497 | 2.01843E-71 | 30401456 |
|  |  |  | cg25862470 | 10 | 99148114 | RRP12 | + | 0.025 | 0.00139497 | 2.01843E-71 | 30401456 |
|  |  |  | cg06359132 | 10 | 99160096 | RRP12 | + | -0.029 | 0.00325007 | 4.78198E-19 | 30401456 |
|  |  |  | cg06359132 | 10 | 99160096 | RRP12 | + | -0.029 | 0.00325007 | 4.78198E-19 | 30401456 |
|  |  |  | cg06359132 | 10 | 99160096 | RRP12 | + | -0.029 | 0.00325007 | 4.78198E-19 | 30401456 |
|  |  |  | cg20016023 | 10 | 99160130 | RRP12 | + | -0.041 | 0.0027912 | 3.93031E-49 | 30401456 |
|  |  |  | cg20016023 | 10 | 99160130 | RRP12 | + | -0.041 | 0.0027912 | 3.93031E-49 | 30401456 |
|  |  |  | cg20016023 | 10 | 99160130 | RRP12 | + | -0.041 | 0.0027912 | 3.93031E-49 | 30401456 |
|  |  |  | cg04175911 | 10 | 99172893 |  | + | -0.009 | 0.00125755 | 1.08884E-12 | 30401456 |
|  |  |  | cg04175911 | 10 | 99172893 |  | + | -0.009 | 0.00125755 | 1.08884E-12 | 30401456 |
|  |  |  | cg04175911 | 10 | 99172893 |  | + | -0.009 | 0.00125755 | 1.08884E-12 | 30401456 |
|  |  |  | cg07192409 | 10 | 99173040 |  | + | -0.009 | 0.00137575 | 1.09039E-10 | 30401456 |
|  |  |  | cg07192409 | 10 | 99173040 |  | + | -0.009 | 0.00137575 | 1.09039E-10 | 30401456 |
|  |  |  | cg07192409 | 10 | 99173040 |  | + | -0.009 | 0.00137575 | 1.09039E-10 | 30401456 |
|  |  |  | cg25507584 | 10 | 99173832 |  | + | -0.016 | 0.00116121 | 1.59477E-43 | 30401456 |
|  |  |  | cg25507584 | 10 | 99173832 |  | + | -0.016 | 0.00116121 | 1.59477E-43 | 30401456 |
|  |  |  | cg25507584 | 10 | 99173832 |  | + | -0.016 | 0.00116121 | 1.59477E-43 | 30401456 |
|  |  |  | cg26545845 | 10 | 99175095 |  | + | 0.010 | 0.00135917 | 2.89163E-14 | 30401456 |
|  |  |  | cg26545845 | 10 | 99175095 |  | + | 0.010 | 0.00135917 | 2.89163E-14 | 30401456 |
|  |  |  | cg26545845 | 10 | 99175095 |  | + | 0.010 | 0.00135917 | 2.89163E-14 | 30401456 |
|  |  |  | cg07052063 | 10 | 99255236 | MMS19 | + | 0.017 | 0.00207013 | 3.93699E-17 | 30401456 |
|  |  |  | cg07052063 | 10 | 99255236 | MMS19 | + | 0.017 | 0.00207013 | 3.93699E-17 | 30401456 |
|  |  |  | cg07052063 | 10 | 99255236 | MMS19 | + | 0.017 | 0.00207013 | 3.93699E-17 | 30401456 |
|  |  |  | cg20702121 | 10 | 99090072 |  | N | -0.599 | 0.0347024 | 8.36568E-67 | 30514905; 29500431 |
|  |  |  | cg24590430 | 10 | 99097076 |  | N | -0.555 | 0.0352661 | 9.0908E-56 | 30514905; 29500431 |
|  |  |  | cg13630239 | 10 | 99116547 |  | N | -0.844 | 0.032559 | 3.5903E-148 | 30514905; 29500431 |
|  |  |  | cg06359132 | 10 | 99160096 |  | N | -0.412 | 0.0361028 | 3.75578E-30 | 30514905; 29500431 |
|  |  |  | cg20016023 | 10 | 99160130 |  | N | -0.595 | 0.034939 | 3.94194E-65 | 30514905; 29500431 |
|  |  |  | cg08345082 | 10 | 99160200 |  | N | -0.455 | 0.0355963 | 2.21541E-37 | 30514905; 29500431 |
|  |  |  | cg07052063 | 10 | 99255236 |  | N | 0.275 | 0.0361862 | 2.90272E-14 | 30514905; 29500431 |
|  |  |  | cg20702121 | 10 | 99090072 | FRAT2 | - | -0.707 | 0.0342202 | 1.06715E-94 | 38548728 |
|  |  |  | cg24590430 | 10 | 99097076 | FRAT2 | - | -0.688 | 0.0339347 | 1.93755E-91 | 38548728 |
|  |  |  | cg13630239 | 10 | 99116547 | FRAT2 | - | -1.082 | 0.0298397 | 6.1779E-288 | 38548728 |
|  |  |  | cg06359132 | 10 | 99160096 | RRP12 | - | -0.697 | 0.0338628 | 2.91777E-94 | 38548728 |
|  |  |  | cg20016023 | 10 | 99160130 | RRP12 | - | -0.924 | 0.0316733 | 4.575E-187 | 38548728 |
|  |  |  | cg08345082 | 10 | 99160200 | RRP12 | - | -0.715 | 0.0339379 | 1.70631E-98 | 38548728 |
|  |  |  | cg25603927 | 10 | 99185264 | PGAM1 | + | 0.245 | 0.0364562 | 1.71058E-11 | 38548728 |
|  |  |  | cg07151406 | 10 | 99185290 | PGAM1 | + | 0.313 | 0.0364874 | 8.892E-18 | 38548728 |
|  |  |  | cg07221526 | 10 | 99185307 | PGAM1 | + | 0.286 | 0.0366167 | 5.81576E-15 | 38548728 |
|  |  |  | cg07052063 | 10 | 99255236 | MMS19 | - | 0.272 | 0.0362143 | 5.90664E-14 | 38548728 |
|  |  |  | cg25247692 | 10 | 99313390 | ANKRD2 | + | 0.260 | 0.0365195 | 1.04921E-12 | 38548728 |
| rs694739 | 11 | 64097233 | cg13682317 | 11 | 63898274 | MACROD1 | + | 0.023 | 0.00352278 | 3.34584E-11 | 30401456 |
|  |  |  | cg09300426 | 11 | 63952618 | STIP1 | + | -0.005 | 0.000892153 | 3.86549E-09 | 30401456 |
|  |  |  | cg10468373 | 11 | 64009913 | FKBP2 | + | -0.008 | 0.00122101 | 1.04234E-11 | 30401456 |
|  |  |  | cg02228329 | 11 | 64053129 | BAD;GPR137 | + | 0.002 | 0.000329971 | 2.27825E-12 | 30401456 |
|  |  |  | cg23796481 | 11 | 64053134 | BAD;GPR137 | + | 0.009 | 0.000702069 | 1.73964E-37 | 30401456 |
|  |  |  | cg10680210 | 11 | 64107158 | CCDC88B | + | -0.015 | 0.00176551 | 2.63825E-17 | 30401456 |
|  |  |  | cg20975835 | 11 | 64107374 | CCDC88B | + | -0.009 | 0.000882931 | 8.32734E-27 | 30401456 |
|  |  |  | cg07884764 | 11 | 64107517 | CCDC88B | + | 0.009 | 0.00109659 | 1.41651E-17 | 30401456 |
|  |  |  | cg00022866 | 11 | 64108440 | CCDC88B | + | 0.030 | 0.00248676 | 4.69642E-33 | 30401456 |
|  |  |  | cg04422903 | 11 | 64108550 | CCDC88B | + | 0.013 | 0.00161219 | 1.32545E-16 | 30401456 |
|  |  |  | cg26707686 | 11 | 64115133 | CCDC88B | + | -0.009 | 0.00137085 | 2.68117E-10 | 30401456 |
|  |  |  | cg10730362 | 11 | 64125599 | RPS6KA4 | + | -0.006 | 0.00106312 | 4.35668E-09 | 30401456 |
|  |  |  | cg20930290 | 11 | 64138764 | RPS6KA4 | + | -0.013 | 0.00127648 | 6.76786E-26 | 30401456 |
|  |  |  | cg06485381 | 11 | 64154072 |  | + | 0.013 | 0.00132292 | 7.26748E-24 | 30401456 |
|  |  |  | cg03074692 | 11 | 64216380 |  | + | 0.007 | 0.000963518 | 1.97615E-13 | 30401456 |
|  |  |  | cg06497934 | 11 | 63899087 |  | N | 0.213 | 0.0335342 | 2.29668E-10 | 30514905; 29500431 |
|  |  |  | cg04369964 | 11 | 63997586 |  | N | -0.213 | 0.0324137 | 5.28108E-11 | 30514905; 29500431 |
|  |  |  | cg12423493 | 11 | 64007813 |  | N | 0.198 | 0.0336892 | 4.43784E-09 | 30514905; 29500431 |
|  |  |  | cg16977872 | 11 | 64008132 |  | N | 0.199 | 0.0333544 | 2.25307E-09 | 30514905; 29500431 |
|  |  |  | cg10468373 | 11 | 64009913 |  | N | -0.324 | 0.0335058 | 4.44564E-22 | 30514905; 29500431 |
|  |  |  | cg26204322 | 11 | 64018687 |  | N | -0.187 | 0.0327771 | 1.19178E-08 | 30514905; 29500431 |
|  |  |  | cg25069102 | 11 | 64039302 |  | N | 0.203 | 0.0331212 | 8.38004E-10 | 30514905; 29500431 |
|  |  |  | cg23796481 | 11 | 64053134 |  | N | 0.530 | 0.031372 | 4.98065E-64 | 30514905; 29500431 |
|  |  |  | cg10680210 | 11 | 64107158 |  | N | -0.338 | 0.0338778 | 1.70981E-23 | 30514905; 29500431 |
|  |  |  | cg20975835 | 11 | 64107374 |  | N | -0.412 | 0.0334362 | 7.67164E-35 | 30514905; 29500431 |
|  |  |  | cg07884764 | 11 | 64107517 |  | N | 0.325 | 0.0336288 | 4.08859E-22 | 30514905; 29500431 |
|  |  |  | cg09619347 | 11 | 64107520 |  | N | 0.520 | 0.0331161 | 1.16671E-55 | 30514905; 29500431 |
|  |  |  | cg00022866 | 11 | 64108440 |  | N | 0.438 | 0.033236 | 1.14731E-39 | 30514905; 29500431 |
|  |  |  | cg04422903 | 11 | 64108550 |  | N | 0.268 | 0.0335073 | 1.22298E-15 | 30514905; 29500431 |
|  |  |  | cg20930290 | 11 | 64138764 |  | N | -0.303 | 0.033467 | 1.21956E-19 | 30514905; 29500431 |
|  |  |  | cg27022558 | 11 | 64141041 |  | N | -0.199 | 0.0335994 | 2.90012E-09 | 30514905; 29500431 |
|  |  |  | cg15605307 | 11 | 64146487 |  | N | 0.194 | 0.032877 | 3.43501E-09 | 30514905; 29500431 |
|  |  |  | cg10470368 | 11 | 64146517 |  | N | -0.801 | 0.0294585 | 9.3073E-163 | 30514905; 29500431 |
|  |  |  | cg03074692 | 11 | 64216380 |  | N | 0.262 | 0.0323415 | 5.25453E-16 | 30514905; 29500431 |
|  |  |  | cg14659698 | 11 | 64216417 |  | N | 0.319 | 0.0335666 | 2.3254E-21 | 30514905; 29500431 |
|  |  |  | cg24431193 | 11 | 63883947 | FLRT1 | + | -0.351 | 0.0380255 | 2.84124E-20 | 38548728 |
|  |  |  | cg14350224 | 11 | 63885769 | FLRT1 | + | 0.225 | 0.0382284 | 4.13176E-09 | 38548728 |
|  |  |  | cg15188398 | 11 | 63912722 | MACROD1 | - | -0.318 | 0.0382623 | 8.88416E-17 | 38548728 |
|  |  |  | cg04000281 | 11 | 63949212 | STIP1 | + | -0.383 | 0.0381051 | 8.55034E-24 | 38548728 |
|  |  |  | cg15432711 | 11 | 63952213 | STIP1 | + | -0.336 | 0.0384964 | 2.74863E-18 | 38548728 |
|  |  |  | cg18225595 | 11 | 63971243 | FERMT3 | + | 0.314 | 0.0382101 | 2.28079E-16 | 38548728 |
|  |  |  | cg19465662 | 11 | 63993652 | TRPT1 | - | 0.343 | 0.0375786 | 7.20367E-20 | 38548728 |
|  |  |  | cg03236948 | 11 | 63997492 | DNAJC4 | + | 0.260 | 0.0383585 | 1.16899E-11 | 38548728 |
|  |  |  | cg09854726 | 11 | 64007569 | FKBP2 | + | -0.234 | 0.0387963 | 1.56065E-09 | 38548728 |
|  |  |  | cg12423493 | 11 | 64007813 | FKBP2 | + | -0.234 | 0.0385049 | 1.17459E-09 | 38548728 |
|  |  |  | cg19006947 | 11 | 64034861 | PLCB3 | + | 0.568 | 0.0369717 | 3.2122E-53 | 38548728 |
|  |  |  | cg18375707 | 11 | 64034959 | PLCB3 | + | 0.538 | 0.0369976 | 7.1649E-48 | 38548728 |
|  |  |  | cg27651452 | 11 | 64035061 | PLCB3 | + | 0.363 | 0.0381223 | 1.79486E-21 | 38548728 |
|  |  |  | cg02228329 | 11 | 64053129 | GPR137 | + | 0.540 | 0.0376108 | 9.80375E-47 | 38548728 |
|  |  |  | cg23796481 | 11 | 64053134 | GPR137 | + | 0.587 | 0.0369711 | 1.0787E-56 | 38548728 |
|  |  |  | cg10680210 | 11 | 64107158 | CCDC88B | + | -0.466 | 0.0372984 | 8.97436E-36 | 38548728 |
|  |  |  | cg20975835 | 11 | 64107374 | CCDC88B | + | -0.262 | 0.0384273 | 1.00554E-11 | 38548728 |
|  |  |  | cg07884764 | 11 | 64107517 | CCDC88B | + | 0.433 | 0.0379202 | 3.25576E-30 | 38548728 |
|  |  |  | cg09619347 | 11 | 64107520 | CCDC88B | + | 0.621 | 0.0363233 | 1.91539E-65 | 38548728 |
|  |  |  | cg01577604 | 11 | 64109823 | MIR7155 | - | 0.235 | 0.0386953 | 1.24612E-09 | 38548728 |
|  |  |  | cg00490203 | 11 | 64115989 | MIR7155 | - | 0.233 | 0.0383685 | 1.3006E-09 | 38548728 |
|  |  |  | cg12797157 | 11 | 64116165 | MIR7155 | - | 0.235 | 0.038896 | 1.57137E-09 | 38548728 |
|  |  |  | cg01998806 | 11 | 64116279 | MIR7155 | - | 0.255 | 0.0383591 | 3.16148E-11 | 38548728 |
|  |  |  | cg27470087 | 11 | 64127816 | RPS6KA4 | + | 0.236 | 0.0383861 | 7.7678E-10 | 38548728 |
|  |  |  | cg20930290 | 11 | 64138764 | MIR1237 | + | -0.300 | 0.0382789 | 4.22987E-15 | 38548728 |
|  |  |  | cg18585107 | 11 | 64138839 | MIR1237 | + | 0.243 | 0.0384919 | 2.65186E-10 | 38548728 |
|  |  |  | cg27022558 | 11 | 64141041 | MIR1237 | + | -0.345 | 0.0382228 | 1.61794E-19 | 38548728 |
|  |  |  | cg22380161 | 11 | 64310371 | SLC22A11 | + | -0.254 | 0.0383685 | 3.49164E-11 | 38548728 |
|  |  |  | cg26780677 | 11 | 64546109 | SF1 | - | 0.214 | 0.0385515 | 2.6704E-08 | 38548728 |
| rs12826560 | 12 | 10532965 | cg26485376 | 12 | 10516240 |  | + | 0.040 | 0.00472266 | 1.10169E-17 | 30401456 |
|  |  |  | cg15368872 | 12 | 10525233 | KLRK1 | + | -0.020 | 0.00234682 | 2.75314E-17 | 30401456 |
|  |  |  | cg08936757 | 12 | 10543994 | KLRK1;LOC101928100;KLRC4-KLRK1 | + | 0.024 | 0.00409183 | 2.33885E-09 | 30401456 |
|  |  |  | cg04131958 | 12 | 10560526 | KLRC4;KLRC4-KLRK1 | + | 0.029 | 0.00327317 | 2.26365E-18 | 30401456 |
|  |  |  | cg10195814 | 12 | 10560602 | KLRC4 | + | 0.014 | 0.00194103 | 1.04372E-13 | 30401456 |
|  |  |  | cg06264089 | 12 | 10563947 | KLRC4-KLRK1 | + | 0.198 | 0.0123871 | 1.63318E-57 | 30401456 |
|  |  |  | cg04531182 | 12 | 10563981 | KLRC4-KLRK1 | + | 0.236 | 0.014271 | 1.89825E-61 | 30401456 |
|  |  |  | cg08041188 | 12 | 10564015 | KLRC4-KLRK1 | + | 0.229 | 0.0140559 | 1.40063E-59 | 30401456 |
|  |  |  | cg12866867 | 12 | 10565229 | KLRC3 | + | 0.042 | 0.00463195 | 6.33717E-20 | 30401456 |
|  |  |  | cg15368872 | 12 | 10525233 |  | N | -0.325 | 0.0539567 | 1.6543E-09 | 30514905; 29500431 |
|  |  |  | cg03394309 | 12 | 10544636 |  | N | -0.332 | 0.0535637 | 5.62759E-10 | 30514905; 29500431 |
|  |  |  | cg20694715 | 12 | 10766273 |  | N | 0.324 | 0.052506 | 6.93435E-10 | 30514905; 29500431 |
|  |  |  | cg22509679 | 12 | 10379548 | GABARAPL1 | + | -0.279 | 0.0380578 | 2.50266E-13 | 38548728 |
|  |  |  | cg03394309 | 12 | 10544636 | KLRK1 | - | -0.254 | 0.0378069 | 1.77601E-11 | 38548728 |
|  |  |  | cg10195814 | 12 | 10560602 | KLRC4 | - | 0.282 | 0.0378645 | 1.00247E-13 | 38548728 |
|  |  |  | cg20047055 | 12 | 10608067 | KLRC1 | - | 0.235 | 0.0381706 | 7.27671E-10 | 38548728 |
|  |  |  | cg05152903 | 12 | 10872218 | YBX3 | - | -0.263 | 0.0378899 | 3.92887E-12 | 38548728 |
| rs79357521 | 12 | 10544978 | cg26485376 | 12 | 10516240 |  | + | 0.040 | 0.00469426 | 1.12875E-17 | 30401456 |
|  |  |  | cg15368872 | 12 | 10525233 | KLRK1 | + | -0.020 | 0.00233282 | 2.98172E-17 | 30401456 |
|  |  |  | cg08936757 | 12 | 10543994 | KLRK1;LOC101928100;KLRC4-KLRK1 | + | 0.024 | 0.00406938 | 4.43304E-09 | 30401456 |
|  |  |  | cg04131958 | 12 | 10560526 | KLRC4;KLRC4-KLRK1 | + | 0.029 | 0.00325291 | 1.88293E-18 | 30401456 |
|  |  |  | cg10195814 | 12 | 10560602 | KLRC4 | + | 0.014 | 0.00192875 | 7.41626E-14 | 30401456 |
|  |  |  | cg06264089 | 12 | 10563947 | KLRC4-KLRK1 | + | 0.197 | 0.0123039 | 6.44169E-58 | 30401456 |
|  |  |  | cg04531182 | 12 | 10563981 | KLRC4-KLRK1 | + | 0.235 | 0.0141782 | 9.90936E-62 | 30401456 |
|  |  |  | cg08041188 | 12 | 10564015 | KLRC4-KLRK1 | + | 0.228 | 0.0139636 | 6.7289E-60 | 30401456 |
|  |  |  | cg12866867 | 12 | 10565229 | KLRC3 | + | 0.042 | 0.00460278 | 4.63561E-20 | 30401456 |
|  |  |  | cg15368872 | 12 | 10525233 |  | N | -0.314 | 0.0532777 | 3.62334E-09 | 30514905; 29500431 |
|  |  |  | cg03394309 | 12 | 10544636 |  | N | -0.323 | 0.0528723 | 9.68465E-10 | 30514905; 29500431 |
|  |  |  | cg20694715 | 12 | 10766273 |  | N | 0.301 | 0.05049 | 2.39198E-09 | 30514905; 29500431 |
|  |  |  | cg13014531 | 12 | 10366500 | GABARAPL1 | + | -0.214 | 0.0379672 | 1.86865E-08 | 38548728 |
|  |  |  | cg22509679 | 12 | 10379548 | GABARAPL1 | + | -0.286 | 0.0381781 | 6.64976E-14 | 38548728 |
|  |  |  | cg03394309 | 12 | 10544636 | KLRK1 | - | -0.257 | 0.0379366 | 1.28588E-11 | 38548728 |
|  |  |  | cg10195814 | 12 | 10560602 | KLRC4 | - | 0.295 | 0.0379584 | 7.08901E-15 | 38548728 |
|  |  |  | cg20047055 | 12 | 10608067 | KLRC1 | - | 0.234 | 0.0383137 | 1.06353E-09 | 38548728 |
|  |  |  | cg05152903 | 12 | 10872218 | YBX3 | - | -0.268 | 0.0380175 | 1.76501E-12 | 38548728 |
| rs56083744 | 14 | 103292191 | cg09978105 | 14 | 103227646 |  | + | -0.027 | 0.00418956 | 1.0454E-10 | 30401456 |
|  |  |  | cg01565703 | 14 | 103245090 | TRAF3 | + | -0.011 | 0.00161928 | 4.31573E-12 | 30401456 |
|  |  |  | cg13979304 | 14 | 103250954 | TRAF3 | + | 0.010 | 0.00176899 | 3.97843E-09 | 30401456 |
|  |  |  | cg08794574 | 14 | 103292047 | TRAF3 | + | 0.008 | 0.0011979 | 2.95542E-11 | 30401456 |
|  |  |  | cg07319276 | 14 | 103348741 | TRAF3 | + | -0.014 | 0.00150339 | 2.80257E-20 | 30401456 |
|  |  |  | cg22078571 | 14 | 103388915 | AMN | + | 0.011 | 0.00172304 | 4.73948E-11 | 30401456 |
|  |  |  | cg09978105 | 14 | 103227646 |  | N | -0.334 | 0.0440941 | 3.5832E-14 | 30514905; 29500431 |
|  |  |  | cg01565703 | 14 | 103245090 |  | N | -0.433 | 0.0444843 | 2.20932E-22 | 30514905; 29500431 |
|  |  |  | cg21920469 | 14 | 103375193 |  | N | -0.755 | 0.0421338 | 7.8703E-72 | 30514905; 29500431 |
|  |  |  | cg18924816 | 14 | 103388762 |  | N | 0.315 | 0.0439384 | 7.74859E-13 | 30514905; 29500431 |
|  |  |  | cg25017060 | 14 | 103389017 |  | N | 0.266 | 0.044389 | 1.93032E-09 | 30514905; 29500431 |
|  |  |  | cg24810305 | 14 | 103389022 |  | N | 0.247 | 0.044721 | 3.22358E-08 | 30514905; 29500431 |
|  |  |  | cg09978105 | 14 | 103227646 |  | N | -0.337 | 0.031443 | 7.69257E-27 | 38548728 |
|  |  |  | cg01565703 | 14 | 103245090 |  | N | -0.396 | 0.0313987 | 2.16071E-36 | 38548728 |
|  |  |  | cg18185554 | 14 | 103294574 |  | N | 0.173 | 0.0314065 | 3.445E-08 | 38548728 |
|  |  |  | cg26115667 | 14 | 103294656 |  | N | 0.204 | 0.0315176 | 9.62493E-11 | 38548728 |
|  |  |  | cg10920224 | 14 | 103367591 |  | N | -0.198 | 0.0316188 | 3.54125E-10 | 38548728 |
|  |  |  | cg21920469 | 14 | 103375193 |  | N | -0.448 | 0.0310866 | 4.17968E-47 | 38548728 |
|  |  |  | cg18924816 | 14 | 103388762 |  | N | 0.222 | 0.0314445 | 1.57568E-12 | 38548728 |
|  |  |  | cg25017060 | 14 | 103389017 |  | N | 0.214 | 0.0316385 | 1.36463E-11 | 38548728 |
|  |  |  | cg24810305 | 14 | 103389022 |  | N | 0.206 | 0.0316421 | 7.68654E-11 | 38548728 |
|  |  |  | cg10087771 | 14 | 103399429 |  | N | -0.231 | 0.0315813 | 2.71052E-13 | 38548728 |
|  |  |  | cg19561503 | 14 | 103433986 |  | N | 0.364 | 0.031427 | 5.98841E-31 | 38548728 |
|  |  |  | cg18364858 | 14 | 103227298 | TRAF3 | + | -0.314 | 0.030775 | 1.75027E-24 | 38548728 |
|  |  |  | cg09978105 | 14 | 103227646 | TRAF3 | + | -0.410 | 0.0303382 | 1.25707E-41 | 38548728 |
|  |  |  | cg01565703 | 14 | 103245090 | TRAF3 | + | -0.595 | 0.0293273 | 1.15503E-91 | 38548728 |
|  |  |  | cg26115667 | 14 | 103294656 | TRAF3 | + | 0.268 | 0.0308881 | 3.73266E-18 | 38548728 |
|  |  |  | cg23520688 | 14 | 103294740 | TRAF3 | + | 0.242 | 0.0309594 | 4.81247E-15 | 38548728 |
|  |  |  | cg14886849 | 14 | 103367489 | AMN | + | -0.197 | 0.0311477 | 2.58725E-10 | 38548728 |
|  |  |  | cg10920224 | 14 | 103367591 | AMN | + | -0.229 | 0.0309364 | 1.35841E-13 | 38548728 |
|  |  |  | cg18924816 | 14 | 103388762 | AMN | + | 0.234 | 0.0311884 | 6.21542E-14 | 38548728 |
|  |  |  | cg01759155 | 14 | 103388797 | AMN | + | 0.230 | 0.0312424 | 1.8311E-13 | 38548728 |
|  |  |  | cg20043258 | 14 | 103468582 | CDC42BPB | - | 0.215 | 0.0306136 | 2.42932E-12 | 38548728 |
| rs6575931 | 14 | 103301574 | cg01565703 | 14 | 103245090 | TRAF3 | + | -0.011 | 0.00152453 | 2.20572E-12 | 30401456 |
|  |  |  | cg08794574 | 14 | 103292047 | TRAF3 | + | 0.008 | 0.0011262 | 2.89529E-12 | 30401456 |
|  |  |  | cg23520688 | 14 | 103294740 | TRAF3 | + | 0.004 | 0.000747906 | 6.57795E-09 | 30401456 |
|  |  |  | cg07319276 | 14 | 103348741 | TRAF3 | + | -0.015 | 0.00140231 | 2.003E-25 | 30401456 |
|  |  |  | cg09978105 | 14 | 103227646 |  | N | -0.239 | 0.0392348 | 1.104E-09 | 30514905; 29500431 |
|  |  |  | cg01565703 | 14 | 103245090 |  | N | -0.372 | 0.0388834 | 1.05195E-21 | 30514905; 29500431 |
|  |  |  | cg23520688 | 14 | 103294740 |  | N | 0.240 | 0.0392343 | 1.0178E-09 | 30514905; 29500431 |
|  |  |  | cg19711268 | 14 | 103367858 |  | N | 0.232 | 0.0393332 | 3.67834E-09 | 30514905; 29500431 |
|  |  |  | cg21920469 | 14 | 103375193 |  | N | -0.518 | 0.0379185 | 2.07213E-42 | 30514905; 29500431 |
|  |  |  | cg18924816 | 14 | 103388762 |  | N | 0.251 | 0.0391065 | 1.30527E-10 | 30514905; 29500431 |
|  |  |  | cg26103168 | 14 | 103507914 |  | N | -0.226 | 0.0376884 | 2.0308E-09 | 30514905; 29500431 |
|  |  |  | cg09978105 | 14 | 103227646 |  | N | -0.242 | 0.0282428 | 9.3292E-18 | 38548728 |
|  |  |  | cg01565703 | 14 | 103245090 |  | N | -0.365 | 0.028083 | 1.40623E-38 | 38548728 |
|  |  |  | cg18185554 | 14 | 103294574 |  | N | 0.188 | 0.0281071 | 2.32553E-11 | 38548728 |
|  |  |  | cg26115667 | 14 | 103294656 |  | N | 0.208 | 0.0281939 | 1.42844E-13 | 38548728 |
|  |  |  | cg23520688 | 14 | 103294740 |  | N | 0.154 | 0.0282189 | 4.93165E-08 | 38548728 |
|  |  |  | cg19711268 | 14 | 103367858 |  | N | 0.180 | 0.0283181 | 2.09042E-10 | 38548728 |
|  |  |  | cg21920469 | 14 | 103375193 |  | N | -0.324 | 0.0279526 | 5.30673E-31 | 38548728 |
|  |  |  | cg18924816 | 14 | 103388762 |  | N | 0.189 | 0.028162 | 1.90955E-11 | 38548728 |
|  |  |  | cg08147181 | 14 | 103394355 |  | N | -0.168 | 0.0282949 | 2.72658E-09 | 38548728 |
|  |  |  | cg19561503 | 14 | 103433986 |  | N | 0.379 | 0.0280441 | 1.62973E-41 | 38548728 |
|  |  |  | cg19782190 | 14 | 103487004 |  | N | 0.236 | 0.0282857 | 7.87492E-17 | 38548728 |
|  |  |  | cg18364858 | 14 | 103227298 | TRAF3 | + | -0.284 | 0.0306098 | 1.91714E-20 | 38548728 |
|  |  |  | cg09978105 | 14 | 103227646 | TRAF3 | + | -0.375 | 0.0302365 | 2.49243E-35 | 38548728 |
|  |  |  | cg01565703 | 14 | 103245090 | TRAF3 | + | -0.592 | 0.0290497 | 3.36941E-92 | 38548728 |
|  |  |  | cg26115667 | 14 | 103294656 | TRAF3 | + | 0.262 | 0.0306151 | 1.15599E-17 | 38548728 |
|  |  |  | cg23520688 | 14 | 103294740 | TRAF3 | + | 0.246 | 0.0306563 | 1.05674E-15 | 38548728 |
|  |  |  | cg14886849 | 14 | 103367489 | AMN | + | -0.169 | 0.0309246 | 4.93549E-08 | 38548728 |
|  |  |  | cg10920224 | 14 | 103367591 | AMN | + | -0.193 | 0.0307498 | 3.20293E-10 | 38548728 |
|  |  |  | cg18924816 | 14 | 103388762 | AMN | + | 0.222 | 0.0309274 | 6.33153E-13 | 38548728 |
|  |  |  | cg01759155 | 14 | 103388797 | AMN | + | 0.218 | 0.0309737 | 2.03077E-12 | 38548728 |
|  |  |  | cg08147181 | 14 | 103394355 | AMN | + | -0.174 | 0.0308359 | 1.82559E-08 | 38548728 |
|  |  |  | cg20043258 | 14 | 103468582 | CDC42BPB | - | 0.222 | 0.0303017 | 2.10684E-13 | 38548728 |
| rs1893592 | 21 | 43855067 | cg07374224 | 21 | 43809848 | TMPRSS3 | + | -0.016 | 0.0021628 | 2.67079E-13 | 30401456 |
|  |  |  | cg18187859 | 21 | 43880997 |  | + | -0.017 | 0.00285978 | 1.62748E-09 | 30401456 |
|  |  |  | cg25385366 | 21 | 43809360 |  | N | -0.199 | 0.0352752 | 1.67003E-08 | 30514905; 29500431 |
|  |  |  | cg07374224 | 21 | 43809848 |  | N | -0.270 | 0.0346923 | 6.99137E-15 | 30514905; 29500431 |
|  |  |  | cg25385366 | 21 | 43809360 |  | N | -0.158 | 0.0255484 | 5.87259E-10 | 38548728 |
|  |  |  | cg07374224 | 21 | 43809848 |  | N | -0.203 | 0.0255144 | 1.94309E-15 | 38548728 |
|  |  |  | cg23042151 | 21 | 43824109 | UBASH3A | + | -0.247 | 0.0358364 | 5.72162E-12 | 38548728 |
|  |  |  | cg14174221 | 21 | 43835592 | UBASH3A | + | -0.546 | 0.0346409 | 4.58818E-56 | 38548728 |
| rs116976860 | 22 | 30947033 | cg00243527 | 22 | 30640594 | LIF | - | -0.530 | 0.0817284 | 8.73633E-11 | 38548728 |
|  |  |  | cg03868770 | 22 | 30783737 | RNF215 | - | 0.492 | 0.0819174 | 1.94156E-09 | 38548728 |
|  |  |  | cg03673688 | 22 | 30818470 | MTFP1 | + | -0.765 | 0.0802291 | 1.48184E-21 | 38548728 |
|  |  |  | cg11568314 | 22 | 30947032 | SEC14L6 | - | -1.376 | 0.0728441 | 1.51982E-79 | 38548728 |
|  |  |  | cg06246950 | 22 | 31030853 | SLC35E4 | + | -0.550 | 0.0816231 | 1.55289E-11 | 38548728 |
|  |  |  | cg26842622 | 22 | 31219085 | OSBP2 | + | 0.616 | 0.0812524 | 3.50284E-14 | 38548728 |

SNP: Reference SNP identifier (rsID)

CHR: chromosome

BP: base pair position on GRCh37

Probe: methylation or metabolomic probe ID

Probe_Chr: probe’s chromosome

Probe_bp: probe’s physical position

Gene: gene name

Orientation: DNA strand direction (+ = forward strand, – = reverse strand) of the methylation probe

beta: effect size

se: standard error of beta

*P*-value: association significance

PMID: source dataset

**Table S4. Baseline demographic and clinical characteristics of the Chinese pSSNS replication cohort**

|  | **Controls** | **pSSNS** |
| --- | --- | --- |
| **Sample size** | 2506 | 501 |
| **Male** | 1226 | 373 |
| **Female** | 1280 | 128 |
| **Age** | 58.16 ± 6.01 | 4.98 ± 3.15 |

**Table S5. Replication of eight previously reported risk loci in the Chinese population**

| **SNP** | **CHR** | **BP** | **A1** | **A2** | **OR** | ***P*-value** | **Gene** |
| --- | --- | --- | --- | --- | --- | --- | --- |
| rs55730955 | 2 | 203721233 | A | T | 0.89 | 0.179 | CD28 |
| rs28862935 | 4 | 74768255 | A | G | 2.22 | 1.67E-08 | BTC |
| rs1063355 | 6 | 32659937 | T | G | 0.61 | 1.14E-09 | HLA-DQB1 |
| rs2637678 | 6 | 116466215 | C | T | 0.82 | 0.023 | CALHM6 |
| rs7759971 | 6 | 135425746 | T | C | 1.36 | 9.42E-05 | AHI1 |
| rs10817678 | 9 | 114817177 | G | A | 0.84 | 0.024 | TNFSF15 |
| rs8062322 | 16 | 10998462 | A | C | 0.76 | 0.003 | CLEC16A |
| rs412175 | 19 | 35851201 | C | T | 1.60 | 6.26E-06 | NPHS1 |

SNP: Reference SNP identifier (rsID)

CHR: Chromosome number

BP: Base pair position (GRCh37)

A1: Effect allele

A2: Non-effect allele

OR: Odds ratio for disease association (A1 vs. A2)

*P*-value: Statistical significance of the association

Gene: Gene(s) near or tagged by the SNP

**Table S6. Replication of pleiotropic loci in the Chinese population**

| **SNP** | **CHR** | **BP** | **A1** | **A2** | **OR** | ***P*-value** | **Gene** | **Cytogenetic  band** | **Annotation** |
| --- | --- | --- | --- | --- | --- | --- | --- | --- | --- |
| rs1763839 | 1 | 8413276 | A | G | 0.784117 | 0.26221 | *RERE* | 1p36.23 | new |
| rs75873622 | 1 | 18440062 | G | A | 1.16769 | 0.38275 | *KLHDC7A/IGSF21* | 1p36.13 | new |
| rs3818813 | 1 | 157748535 | G | T | 0.908673 | 0.321499 | *FCRL1/FCRL2/FCRL3* | 1q23.1 | new* |
| rs3771258 | 2 | 61537005 | C | T | 0.985903 | 0.861749 | *XPO1* | 2p15 | new |
| rs3769684 | 2 | 203720036 | C | T | 0.882602 | 0.160867 | *CD28* | 2q33.2 | known |
| rs231805 | 2 | 203844026 | G | A | 1.13323 | 0.24674 | *CTLA4* | 2q33.2 | new |
| rs13429408 | 2 | 218278137 | C | A | 1.108 | 0.197205 | *TMBIM1/PNKD* | 2q35 | new |
| rs34463936 | 5 | 35850047 | T | C | 0.886962 | 0.281529 | *IL7R* | 5p13.2 | new |
| rs9275596 | 6 | 32713854 | C | T | 0.684703 | 0.00015033 | MHC region | 6p21.32-6p21.33 | known |
| rs6478108 | 9 | 114796423 | C | T | 0.865231 | 0.050543 | *TNFSF15* | 9q32 | known |
| rs57943165 | 10 | 63603206 | T | G | 1.18829 | 0.0768107 | *NRBF2/JMJD1C* | 10q21.3 | new* |
| rs1250564 | 10 | 79287585 | C | A | 1.04175 | 0.603313 | *ZMIZ1/PPIF* | 10q22.3 | new* |
| rs7895695 | 10 | 97407498 | A | G | 1.0601 | 0.533225 | *EXOSC1/FAT/RRP12/ARHGAP19* | 10q24.1 | new |
| rs694739 | 11 | 64329761 | G | A | 0.936689 | 0.542028 | *CCDC88B* | 11q13.1 | new |
| rs12826560 | 12 | 10380366 | C | T | 1.03899 | 0.671809 | *KLRC1/KLRC2/KLRC3/KLRC4/KLRK1* | 12p13.2 | new |
| rs56083744 | 14 | 102825854 | A | G | 0.943803 | 0.479803 | *TRAF3* | 14q32.32 | new |
| rs7206753 | 16 | 10974987 | C | T | 0.763592 | 0.0337382 | *CLEC16A* | 16p13.13 | known |
| rs36001636 | 16 | 79315909 | C | T | 0.720699 | 0.00182378 | *MAF/WWOX* | 16q23.2 | new |
| rs8113704 | 19 | 35896979 | G | A | 1.36997 | 0.00258058 | *NFKBID* | 19q13.12 | known |
| rs1893592 | 21 | 42434957 | C | A | 0.904748 | 0.252886 | *UBASH3A* | 21q22.3 | new |

SNP: Reference SNP identifier (rsID)

CHR: Chromosome number

BP: Base pair position (GRCh37)

A1: Effect allele

A2: Non-effect allele

OR: Odds ratio for disease association (A1 vs. A2)

*P*-value: Statistical significance of the association

Gene：Gene(s) located near or tagged by the SNP

Cytogenetic band: Chromosomal band location

Annotation: Classification of the locus

- “new”: Newly identified loci with no prior GWAS reports in pSSNS or IgAN

- “new*”: Previously reported in IgAN GWAS but newly identified in pSSNS

- “known”: Established loci reported in both pSSNS and IgAN GWAS

**Table S7. Differential gene expression of novel pleiotropic loci in pSSNS and IgAN**

| **Locus** | **Gene** | **SteroidSensitive Post treatment VS Pre treatment** | | **IgA case VS control** | |
| --- | --- | --- | --- | --- | --- |
|  |  | ***P*-value** | **W value** | ***P*-value** | **W value** |
| 1p36.23 | RERE | 0.956 | 130 | 0.035 | 136 |
| 1p36.13 | KLHDC7A | 0.652 | 138 | 0.013 | 122 |
|  | IGSF21 | 0.621 | 138 | 9.97E-08 | 432 |
| 1q23.1 | FCRL1 | 0.468 | 148 | 0.116 | 283 |
|  | FCRL2 | 0.365 | 152.5 | 0.371 | 256 |
|  | FCRL3 | 0.065 | 177.5 | 0.002 | 344 |
| 2p15 | XPO1 | 0.136 | 168 | 0.362 | 257 |
| 2q33.2 | CD28 | 6.53E-04 | 215 | 1.57E-06 | 411 |
| 2q33.2 | CTLA4 | 0.226 | 160 | 0.232 | 172 |
| 2q35 | TMBIM1 | 0.445 | 107 | 3.44E-05 | 376 |
|  | PNKD | 0.780 | 120 | 0.047 | 299 |
| 5p13.2 | IL7R | 0.004 | 202 | 5.86E-05 | 372 |
| 9q32 | TNFSF15 | 0.980 | 127 | 2.27E-04 | 361 |
| 10q21.3 | NRBF2 | 0.012 | 62 | 0.891 | 214 |
|  | JMJD1C | 0.616 | 142 | 8.48E-07 | 24 |
| 10q22.3 | ZMIZ1 | 0.210 | 94 | 4.74E-05 | 382 |
|  | PPIF | 0.386 | 104.5 | 0.121 | 282 |
| 10q24.1 | RRP12 | 0.243 | 96.5 | 0.149 | 278 |
|  | EXOSC1 | 0.439 | 149 | 0.080 | 290 |
| 11q13.1 | CCDC88B | 0.692 | 117 | 0.541 | 245 |
| 12p13.2 | KLRC1 | 0.742 | 119 | NaN | NaN |
|  | KLRC2 | 0.158 | 157 | NaN | NaN |
|  | KLRC3 | 0.864 | 123 | 0.006 | 327 |
|  | KLRC4 | 0.078 | 174.5 | 0.911 | 215 |
|  | KLRK1 | 0.323 | 155 | NaN | NaN |
| 14q32.32 | TRAF3 | 0.207 | 94 | 0.489 | 248 |
| 16p13.13 | CLEC16A | 0.897 | 132 | 0.234 | 268 |
| 16q23.2 | MAF | 0.070 | 176.5 | 0.493 | 192 |
|  | WWOX | 0.139 | 167 | 3.31E-04 | 77 |
| 21q22.3 | UBASH3A | 0.250 | 159 | 0.072 | 292 |

SteroidSensitive: differential expression of candidate genes between pre-treatment and post-treatment groups within steroid-sensitive nephrotic syndrome patients

IgA case VS control: differential expression of candidate genes between IgAN patients and healthy controls

Locus: Cytogenetic band or chromosomal region

Gene: Gene name

SteroidSensitive *P*-value: *P*-value for differential expression between post-treatment and pre-treatment groups in steroid-sensitive nephrotic syndrome patients

SteroidSensitive W value: Wilcoxon test statistic (W value) for steroid-sensitive nephrotic syndrome comparison

IgA case VS control *P*-value: *P*-value for differential expression between IgA nephropathy cases and healthy controls

IgA case VS control W value: Wilcoxon test statistic (W value) for IgA nephropathy case vs. control comparison
